# RNA-seq analysis in simulated microgravity unveils down-regulation of the beta-rhizobial siderophore phymabactin

**DOI:** 10.1038/s41526-024-00391-7

**Published:** 2024-04-03

**Authors:** Daphné Golaz, Chad K. Papenfuhs, Paula Bellés-Sancho, Leo Eberl, Marcel Egli, Gabriella Pessi

**Affiliations:** 1 Department of Plant and Microbial biology, University of Zurich, Zurich, Switzerland; 2 School of Engineering and Architecture, Institute of Medical Engineering, Space Biology Group, Lucerne University of Applied Sciences and Arts, Hergiswil, Switzerland; 3 National Center for Biomedical Research in Space, Innovation Cluster Space and Aviation, University of Zurich, Zurich, Switzerland

**Keywords:** Microbiology, Molecular biology

## Abstract

Exploiting the symbiotic interaction between crops and nitrogen-fixing bacteria is a simple and ecological method to promote plant growth in prospective extraterrestrial human outposts. In this study, we performed an RNA-seq analysis to investigate the adaptation of the legume symbiont *Paraburkholderia phymatum* STM815^T^ to simulated microgravity (s0-g) at the transcriptome level. The results revealed a drastic effect on gene expression, with roughly 23% of *P. phymatum* genes being differentially regulated in s0-g. Among those, 951 genes were upregulated and 858 downregulated in the cells grown in s0-g compared to terrestrial gravity (1 g). Several genes involved in posttranslational modification, protein turnover or chaperones encoding were upregulated in s0-g, while those involved in translation, ribosomal structure and biosynthesis, motility or inorganic ions transport were downregulated. Specifically, the whole *phm* gene cluster, previously bioinformatically predicted to be involved in the production of a hypothetical malleobactin-like siderophore, phymabactin, was 20-fold downregulated in microgravity. By constructing a mutant strain (Δ*phmJK*) we confirmed that the *phm* gene cluster codes for the only siderophore secreted by *P. phymatum* as assessed by the complete lack of iron chelating activity of the *P. phymatum* Δ*phmJK* mutant on chrome azurol S (CAS) agar plates. These results not only provide a deeper understanding of the physiology of symbiotic organisms exposed to space-like conditions, but also increase our knowledge of iron acquisition mechanisms in rhizobia.

## Introduction

Nitrogen is the most growth-limiting nutrient in plants that are unable to metabolize atmospheric nitrogen (N_2_) and instead require nitrogen in a mineral form, such as NH_4_, to thrive^1,2^. The common solution in agriculture is to provide nitrogen fertilizers produced through the Haber-Bosch process directly to the crops^3^. This practice is economically and ecologically expensive, as the production of synthetic nitrogen fertilizers represents up to 2% of the worldwide yearly energy utilization^3,4^. Yet, it is possible to utilize the naturally occurring mutualistic relationship between rhizobia and plants to provide nitrogen to crops without using synthetic fertilizers, alleviating the global dependence on agrochemicals^5–8^. In fact, 88% of the members of the Leguminosae family, composed of 19,500 known species, are able to form a symbiotic relationship with rhizobia, which are responsible for up to 60% of worldwide yearly biological nitrogen fixation^9,10^. N_2_-fixing rhizobia are already in use in farming practices to support food crops like soybean (*Glycine* max) and common bean (*Phaseolus vulgaris*) for example^11–13^. In addition, these bacteria are known to produce phytohormones such as auxin and brassinosteroids that stimulate plant growth and rhizogenesis^14–16^. Some rhizobia can also solubilize phosphorus that is immobilized in the soil and is not available for uptake by crops^14^. Furthermore, certain strains of rhizobia can protect plants against diseases, either by stimulating the legumes’ immune system or by inhibiting growth of pathogens^17–19^. Among the well-established methods by which rhizobia can antagonize plants pathogens are the production of antibiotics, other toxins or the secretion of siderophores^14^. Siderophores are iron-chelating compounds secreted by bacteria to sequester iron from their environment, therefore preventing plant pathogenic bacteria from obtaining it^20^. Additionally, siderophores can reduce Fe^3+^ into Fe^2+^ that plants can absorb and metabolize, thereby stimulating their growth^8,21^.

Symbiosis between legumes and rhizobia can occur and facilitate crops growth in extreme environments, such as hypersaline or highly polluted soils^22–24^. Another extreme environment where the interaction between legumes and their symbionts could be used for promoting crop growth is space. The establishment of permanent extraterrestrial colonies will require the development and implementation of space farming, as future settlers will demand access to vitamin and fiber-rich fresh food to ensure their physical and psychological well-being^25–27^. Moreover, upcoming human dwellings on celestial bodies will need to be as self-sufficient as possible to minimize costs and energy spent on importing supplies to promote the fertility of lunar regolith, for example^26^. This self-reliance is not compatible with being dependent on regular shipments of synthetic fertilizers^27,28^. However, reduced gravity encountered at the surface of the Moon or Mars has a negative effect on plants growth, immune system and yield^29,30^. For example, exposure to artificial weightlessness induces direct morphological changes in plants, such as an increased shoot dry mass and root length and a decrease in root dry mass, as it is the case for the legume model *Medicago truncatula*^31^. Moreover, the fungal pathogen *Phytophthora sojae* was more efficient in infecting roots of the legume soybean during the Space Shuttle Mission STS-87, suggesting that the plant immune system is weaker under space-like conditions^32^. Taking advantage of the symbiosis between legumes and rhizobia could hence be a solution to enable farming in extraterrestrial environments^25^. However, very little is currently known about the impact of space conditions on mutualistic plant-bacteria interactions, despite their importance in terrestrial ecosystems and their potential as biofertilizers and biocontrol agents^33,34^. A good rhizobial model to study symbiosis is the betaproteobacterium *Paraburkholderia phymatum* STM815^T^, a versatile symbiont that can nodulate the roots of more than 50 legumes, including crops of high human and economic importance like common bean or cowpea^16,35–37^. *P. phymatum* is also highly competitive for root nodulation and resistant to abiotic stresses^16^.

In order to acquire a better understanding of the effect of weightlessness on symbiotic microorganisms, we used the N_2_-fixing and symbiotic rhizobium *P. phymatum* grown on a random positioning machine (RPM), which generates a microgravity-like environment (s0-g) and performed an RNA-sequencing (RNA-seq) analysis. Our RNA-seq analysis showed that almost one fourth of the annotated 7899 *P. phymatum* genes were differentially regulated. Among those, several genes involved in stress response were upregulated in s0-g compared to terrestrial gravity (1 g), while those responsible for cell replication, protein synthesis, motility, inorganic ion transport or defense mechanisms were downregulated. Interestingly, we observed that all the genes belonging to a cluster encoding a putative siderophore named phymabactin (*phm*)^38^ were downregulated in s0-g. Mutants, in which *phmJ* or *phmK* were inactivated, did not produce any siderophores, suggesting that the *phm* cluster is indeed responsible for the production of the only siderophore produced by *P. phymatum*.

## Results

### Microgravity-triggered changes in *P. phymatum* STM815^T^ transcriptome

**Fig. 1:**
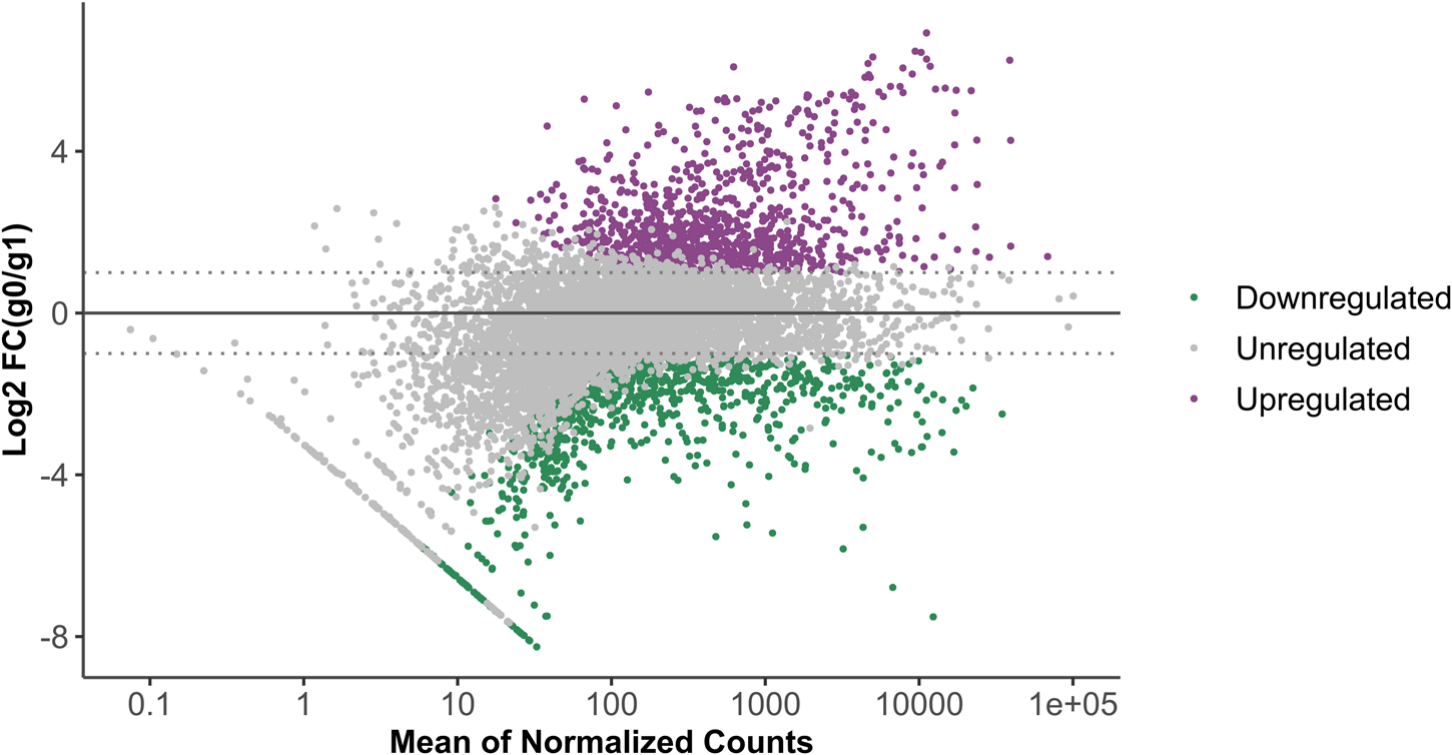
Differential gene expression under simulated microgravity and terrestrial gravity. The MA plot (M=log ratios; A=averages) shows the log_2_ fold changes (FC) in transcript expression in *P. phymatum* STM815^T^ grown in simulated microgravity (g0) versus terrestrial gravity (g1) conditions. The genes with statistically significant increased expression in simulated microgravity are shown in purple, while the ones with decreased expression are displayed in green (*p* ≤ 0.01).

RNA-seq was performed to investigate changes in gene expression in *P. phymatum* cells subjected to s0-g. To do so, wild-type cells were grown in minimal medium cultures either in s0-g or in terrestrial gravity until towards the end of the exponential phase (OD_600_ = 0.7). A growth profile of *P. phymatum* cells incubated in 1 g and in s0-g was conducted prior to sample collection to confirm that exposure to microgravity does not affect the cell replication (Supplementary Fig. 1). We processed three biological replicates per condition and obtained on average 19.2 million reads per sample. Using a threshold *p* ≤ 0.01 and an absolute log_2_ fold change (FC) ≥ 1, we identified 1809 *P. phymatum* genes differentially expressed, among which 858 were downregulated and 951 were upregulated (Fig. 1).

**Fig. 2:**
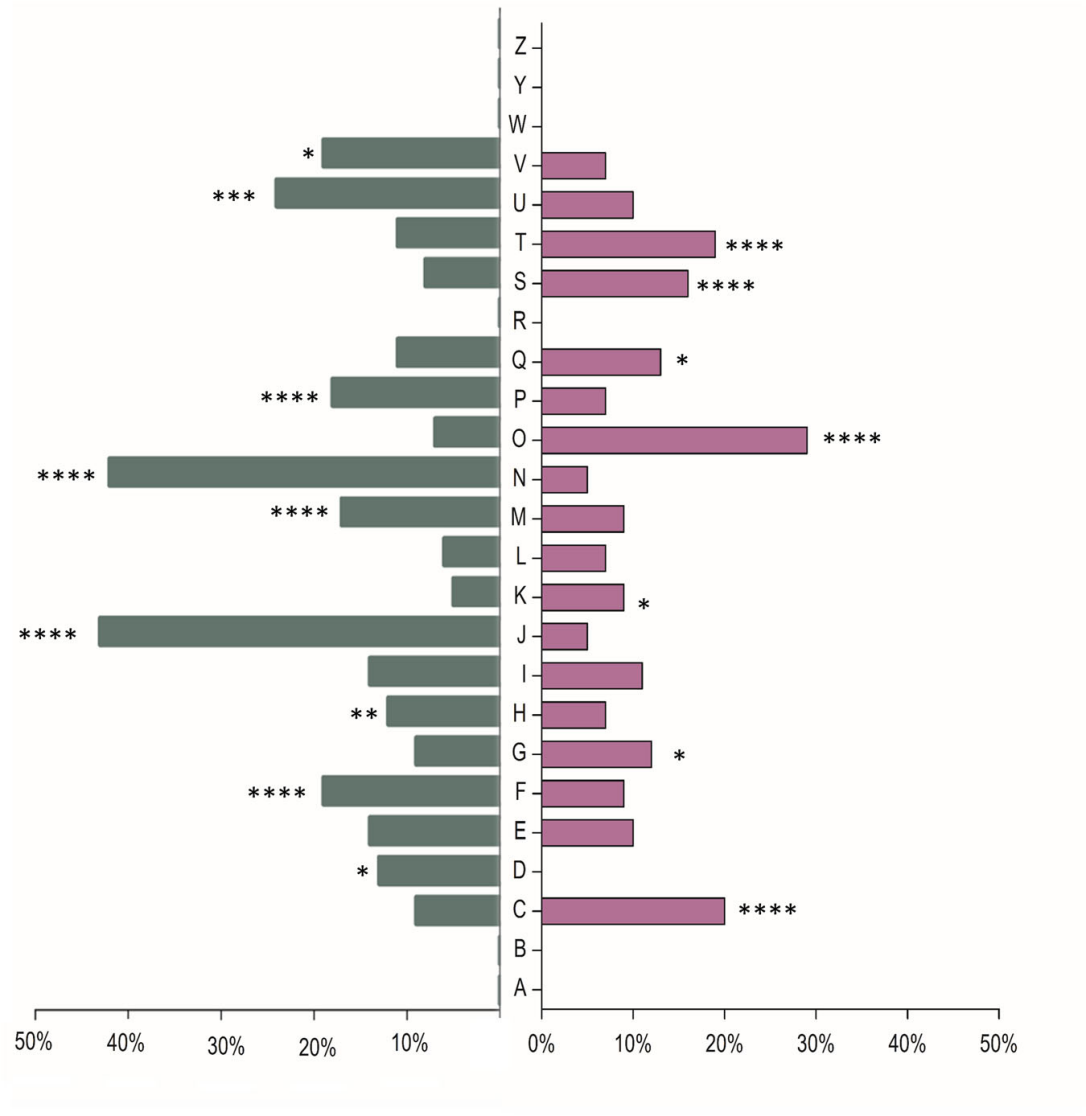
Classification in Cluster of Orthologous Genes (COG) functional categories of the differentially expressed genes (DEG) in s0-g compared to 1 g. The percentage of genes downregulated in s0-g in each category is shown in green, while the percentage of upregulated genes is indicated in purple. The different categories are indicated by the following letters: A, RNA processing and modification; B, chromatin structure and dynamics; C, energy production and conversion; D, cell cycle control, cell division, chromosome partitioning; E, amino acid transport and metabolism; F, nucleotide transport and metabolism; G, carbohydrate transport and metabolism; H, coenzyme transport and metabolism; I, lipid transport and metabolism; J, translation, ribosomal structure and biogenesis; K, transcription; L, replication, recombination and repair; M, cell wall/membrane/envelope biogenesis; N, cell motility; O, posttranslational modification, protein turnover, chaperone; P, inorganic ion transport and metabolism; Q, secondary metabolites biosynthesis, transport and catabolism; R, general function prediction only; S, function unknown; T, signal transduction mechanisms; U, intracellular trafficking, secretion, and vesicular transport; V, defense mechanisms; W, extracellular structures. Statistical analysis was performed through Fisher’s exact test (*p < 0.05, **p < 0.01, ***p < 0.0001, ****p < 0.00001).

The differentially expressed genes (DEG) were assigned to functional categories according to the eggNOG classification system (Fig. 2).

The category O (posttranslational modification, protein turnover, chaperone) is the most over-represented among the upregulated genes in cells grown in s0-g compared to 1 g (*p* < 0.0001). For example, Bphy_2334 and Bphy_1113, coding for the chaperones GroES and GroEL, showed a 23-fold upregulation in s0-g. Genes classified in the category T (signal transduction mechanisms) such as Bphy_6643, which codes for an osmolarity response regulator, and Bphy_5818 encoding a universal stress protein were also significantly upregulated in s0-g compared to 1 g (*p* < 0.0001). Additionally, a high number of genes showing increased expression in s0-g are coding for proteins of unknown function (category S, Fig. 2) (*p* < 0.0001). Furthermore, genes of the category G (carbohydrate transport and metabolism) like Bphy_4389 and Bphy_4390, which are involved in trehalose production and code for a maltooligosyl trehalose trehalohydrolase and the glycogen debranching enzyme GlgX respectively, were also more expressed in s0-g than in 1 g. Several genes belonging to the category K (transcription) also displayed a significantly higher expression in s0-g compared to 1 g. For instance, Bphy_0301, which encodes the heat shock sigma factor RpoH and Bphy_0962 that codes for the stationary phase sigma factor RpoS were both upregulated in s0-g. We also found several genes attributed to the category C (energy production and conversion), such as Bphy_3647 encoding the cytochrome ubiquinol oxidase subunit I CyoB, or Bphy_7754 coding for the nitrogenase molybdenum-iron protein alpha chain, upregulated in microgravity compared to terrestrial gravity (*p* < 0.0001). Moreover, several genes assigned to the category Q (secondary metabolites biosynthesis, transport and catabolism), such as Bphy_7747 and Bphy_7777, both coding for nitrogen fixation proteins FixT, were upregulated in cells grown in s0-g compared to 1 g.

Among the highly downregulated genes in microgravity, we found several genes of the category J (translation, ribosomal structure and biogenesis). For instance, Bphy_2818, encoding the translation initiation factor IF-1, or Bphy_2843, coding for the elongation factor G, showed decreased expression in the cells grown in s0-g compared to 1 g (*p* < 0.0001). Also, genes attributed to the category N (cell motility) such as Bphy_2963, responsible to produce the flagellar biosynthesis protein FlhA, and Bphy_2932, coding for the flagellar motor switch protein FliG were downregulated in s0-g (*p* < 0.0001). Moreover, several genes classified in the category U (intracellular trafficking, secretion and vesicular transport) were downregulated in s0-g (*p* < 0.0004). For instance, Bphy_1947, coding for the biopolymer transporter ExbD and Bphy_2819, coding for the preprotein translocase subunit SecY. Furthermore, genes of the category F (nucleotide transport and metabolism) such as Bphy_2553 that encodes the bifunctional purine biosynthesis protein PurH, or Bphy_0570 that codes for the dUTP diphosphatase Dut were less expressed in s0-g (*p* < 0.0001). The genes belonging to the category V (defense mechanisms), like Bphy_3538 and Bphy_3488, both encoding a putative acriflavine resistance protein, were downregulated in the cells grown in artificial weightlessness compared to the ones grown in terrestrial gravity. Furthermore, several genes of the category M (cell wall/ membrane/ envelope biogenesis) such as Bphy_0051 that codes for the cell rod shape-determining protein MreC, were downregulated in cells grown in s0-g compared to the ground controls (*p* < 0.0001). Several genes attributed to the category D (cell cycle control, cell division, chromosome partitioning) such as Bphy_2457 coding for the cell division coordinator protein CpoB, or Bphy_2089, encoding the cell division protein FtsK were downregulated in cells grown in s0-g compared to the ones in 1 g. One of the strongest downregulated gene in artificial weightlessness was Bphy_4445 (FC = 59.9) that encodes a sugar ABC transporter permease and belongs to category P (inorganic ion transport and metabolism). Interestingly, several downregulated genes in category P are potentially involved in iron transport. Among others, Bphy_1957 that codes for an iron transporter and, Bphy_5030, Bphy_5373 and Bphy_6693, which encode TonB-dependent receptors. In addition, the genes cluster Bphy_4047 to Bphy_4035 that encode proteins involved in the production of a hypothetical siderophore were all downregulated in cells grown in s0-g compared to 1 g (average FC of -20). A complete list of all *P. phymatum* DEG in s0-g compared to terrestrial gravity is listed in Supplementary Table 1.

### Siderophore production is reduced in s0-g

**Fig. 3:**
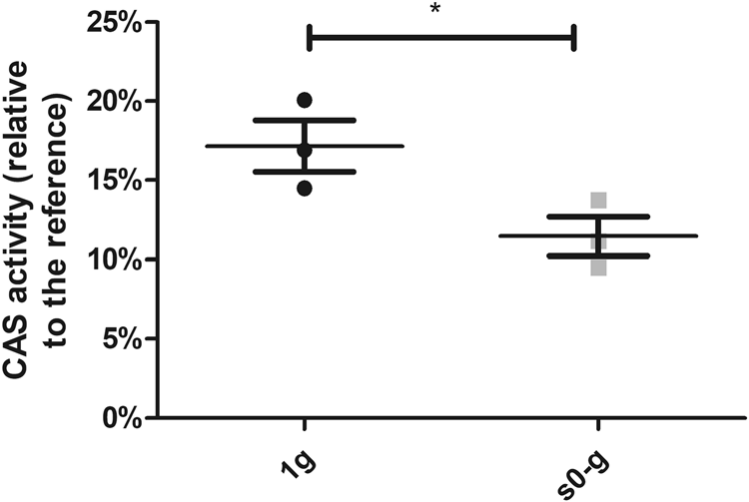
Less siderophore production in s0-g compared to 1 g. The supernatant of *P. phymatum* cells incubated overnight in 1 g and s0-g in ABS minimal medium buffered with PIPES was collected and used for siderophores quantifications using a liquid CAS assay. The CAS activity was calculated using the absorbance at 630 nm of a reference (Ar) and of the sample (As) in the formula Ar-As/As/OD_600_ x100. OD_600_ is the absorbance at 600 nm of the cultures before cells precipitation. The standard deviations are given, and three (*n* = 3) biological replicates were tested. A Student *t* test was performed (*p < 0.05).

To further investigate down-regulation of genes involved in iron uptake under simulated microgravity conditions, siderophore production in terrestrial gravity and artificial weightlessness was quantified through liquid chrome azurol S (CAS) assay. For this, the supernatant of *P. phymatum* cells grown in 1 g and s0-g for 17 h was filtered and mixed with CAS dye. Iron chelation resulted in a colorimetric change that was measured spectrophotometrically. The amount of siderophores produced in 1 g versus s0-g is expressed as the CAS activity relative to the reference (sterile minimal medium with CAS dye), as described in the methods. As shown in Fig. 3, cells grown in s0-g displayed a 66.8% lower CAS activity than when incubated in terrestrial gravity, suggesting that exposure to simulated microgravity impacts *P. phymatum* ability to produce iron chelators.

### The *phm* cluster is downregulated in iron replete conditions and in simulated microgravity

**Fig. 4:**
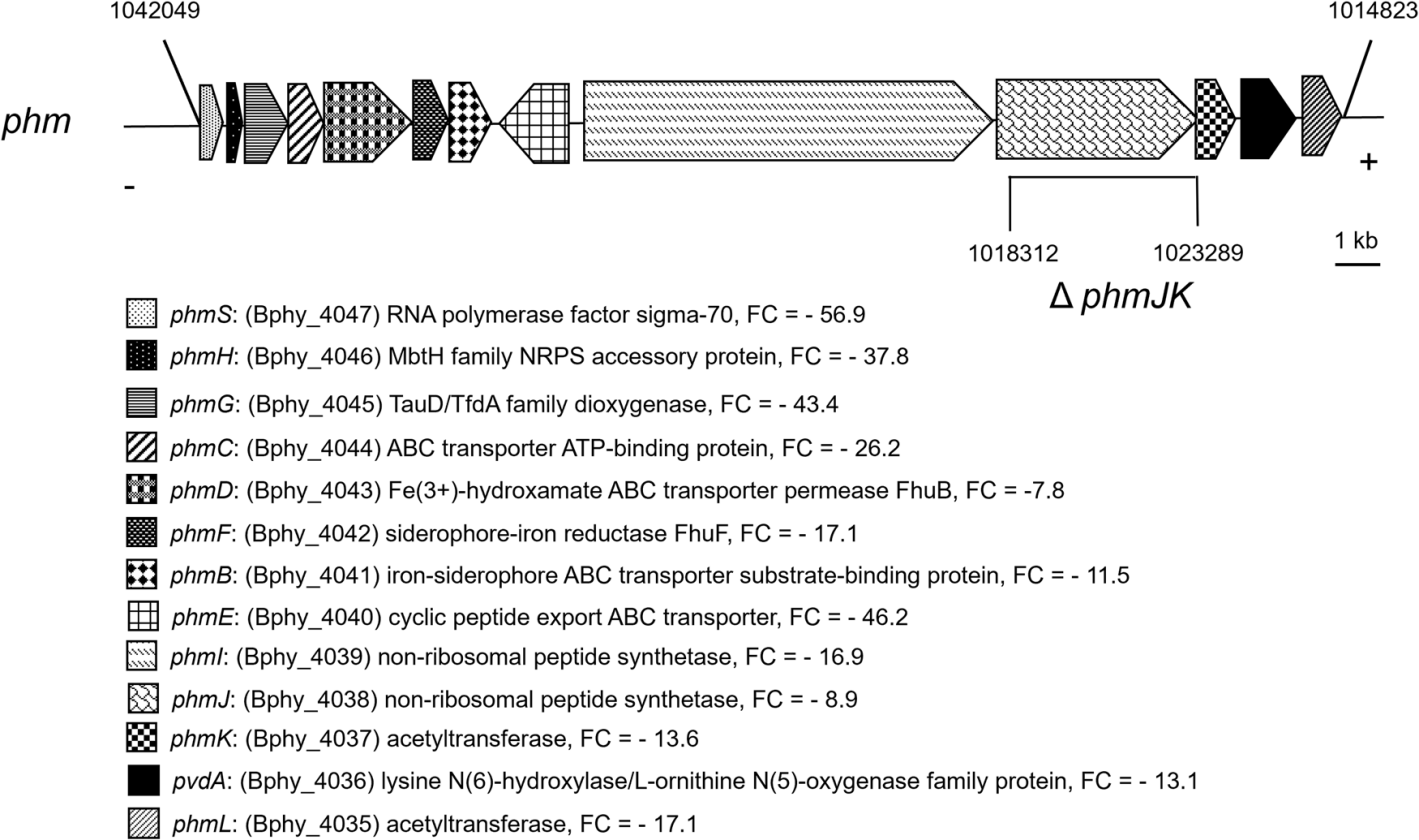
Physical map of the *phm* gene cluster. The numbers on top refer to the nucleotides position according to the Burkholderia database (https://www.burkholderia.com). The deletion in the phymabactin deficient mutant is shown with a Δ*phmJK*. The description and fold change (FC) of expression s0-g versus 1 g of each gene is also indicated.

Our RNA-seq analysis showed that a *P. phymatum* gene cluster previously bioinformatically predicted to be involved in the production of a siderophore was downregulated in cells grown in s0-g^38^ (Fig. 4). The predicted iron chelator was named phymabactin because of its similarity with the siderophores ornibactin and malleobactin, which are synthesized by many *Burkholderia cenocepacia* (*orb* gene cluster) and *Burkholderia pseudomallei* strains (*mba* gene cluster), respectively^38^. The first gene in all three clusters is the regulatory gene *phmS* (Bphy_4047) that encodes an extracytoplasmic sigma 70 factor (ECF), which is roughly 69% similar to OrbS in *B. cenocepacia*. Moreover, *phmC* (Bphy_4044) codes for an ABC transporter ATP-binding protein that shows 75.4% similarity with OrbC. Another gene, *phmD* (Bphy_4043), encodes the iron hydroxamate ABC transporter permease FhuB, which is 66.9% similar to OrbD. Also, *phmB* (Bphy_4041) encodes an iron-siderophore ABC transporter substrate-binding protein that displays 62.9% similarity with OrbB and is followed by a gene coding for a cyclic peptide export ABC transporter PhmE (Bphy_4040), that is 77% similar to OrbE. In average, *phm* displays about 70% and 66% of similarity with the *mba* and *orb* gene clusters, respectively. The similarity of each *P. phymatum phm* gene in the cluster to their homologs in the *mba* and *orb* clusters are listed in the Supplementary Table 2.

A closer look at the *phmS* promoter region revealed the presence of a potential ferric uptake regulator (Fur) binding sequence (GTAAACGCGAATCATTCTC) that is almost identical (18/19 matches; underlined) with the Fur box identified in *B. cenocepacia* 715J^39,40^.

### The *phmJK* deletion mutant showed no siderophore production

**Fig. 5:**
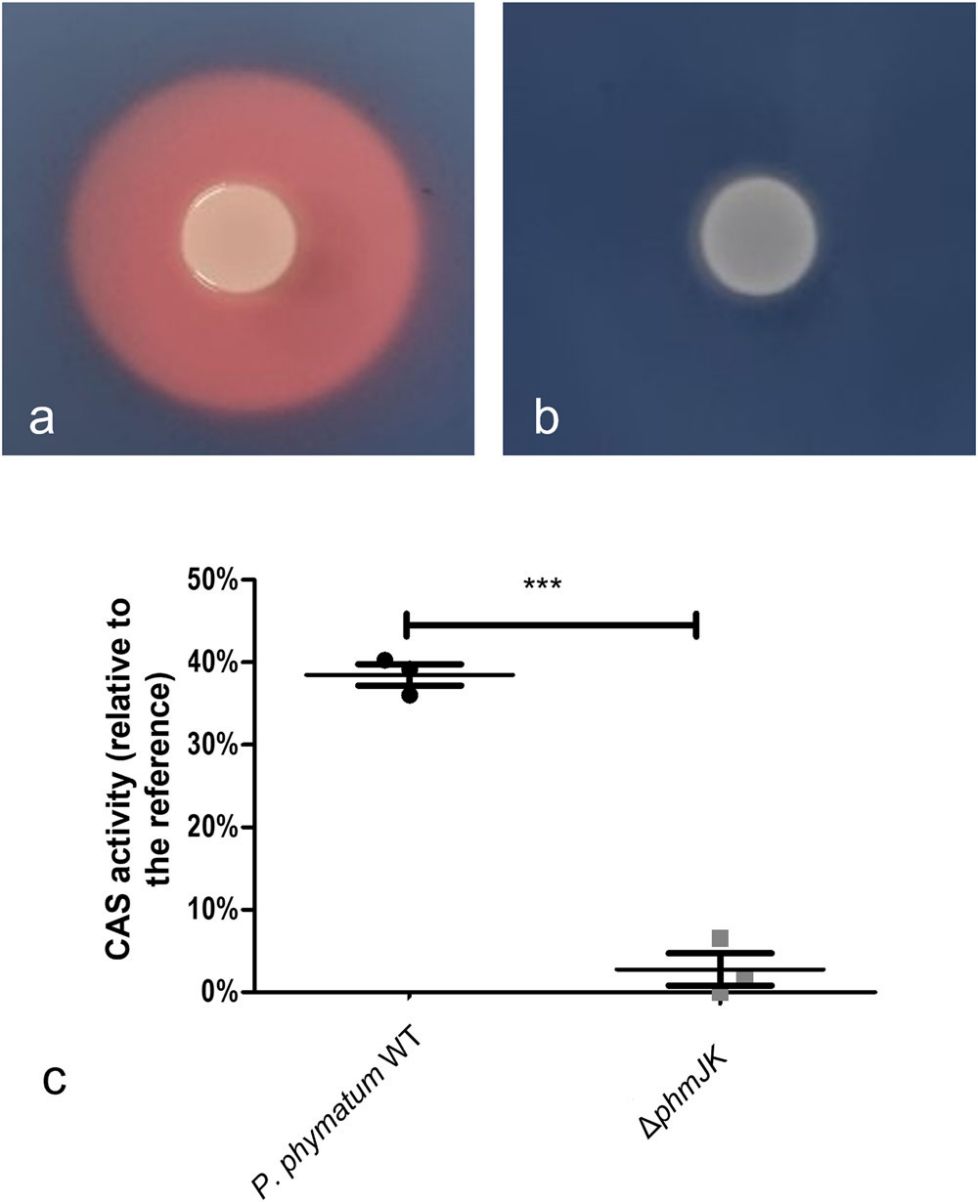
*P. phymatum* Δ*phmJK* does not produce siderophores. CAS assays were performed on *P. phymatum* wild-type (**a**) and Δ*phmJK* deletion mutant (**b**) grown on ABS agar plates. The cells were spotted and incubated for 48 h. Three (*n* = 3) biological replicates were performed and the results of one replicate are shown here. **c** quantification of siderophore production in the supernatant of *P. phymatum* wild-type and Δ*phmJK* grown in buffered liquid ABS minimal medium for 17 h at 28 °C, 180 rpm. The data show the mean value of three independent biological replicates. The standard deviations are shown as bars. Statistical analysis was performed through Student’s *t-*test (***p < 0.001).

A *phm* mutant, in which part of the non-ribosomal peptide-synthetize enzyme (NRPS) encoding gene *phmJ* and the acetyltransferase *phmK* were deleted, was generated and assessed for siderophore production on CAS-agar plates. While an orange halo was observed on the plates where the *P. phymatum* wild-type strain was spotted (Fig. 5a) no change in color was seen on the plates with the *phmJK* mutant (Fig. 5b), suggesting that *phm* codes for the only siderophore in *P. phymatum*. The amount of the siderophore in the supernatant of overnight cultures grown in minimal media was quantified using liquid CAS assay and showed that *P. phymatum* wild-type has a CAS activity relative to the reference (sterile growth media incubated with CAS dye) of 38.5 ± 1.3%, which is approximately 13.8 times higher than the values measured for the Δ*phmJK* mutant (CAS activity of 2.8 ± 1.96%; *p* < 0.0001).

## Discussion

In this study, we performed a transcriptome analysis of the rhizobial strain *P. phymatum* grown in s0-g and assessed for the first time the impact of artificial microgravity on genome expression of a N_2_-fixing legume symbiont using a high throughput method. The RNA-seq analysis revealed that several genes involved in translation, metabolism, biosynthesis, nucleic acids, and inorganic ions pathways were downregulated, while genes coding for chaperones production and proteins turnover were upregulated in artificial weightlessness relative to terrestrial gravity (Fig. 2). For instance, expression of genes coding for the four subunits of the cytochrome o complex ubiquinol in *P. phymatum*, Bphy_3649 to Bphy_3646 (*cyo*), was strongly increased in cells grown in s0-g compared to the ones grown in 1 g. Interestingly, this gene cluster has also been observed to be upregulated in *P. phymatum* grown in microoxic conditions^41^. Moreover, in the endophyte *Paraburkholderia phytofirmans* PsJN, homologous *cyo* genes showed increased expression when exposed to drought stress^37,42,43^. The observed upregulation of the genes encoding the RpoH and RpoS transcription factors, which are involved in heat shock and stationary phase/stress response, respectively, suggest that growth in s0-g induces a similar effect as drought or osmotic stress in *P. phymatum* cells^44^. Yet, growth of *P. phymatum* in microgravity was not impaired compared to terrestrial gravity (Supplementary Fig. 1), which might be explained by the fact that high energy consuming functions such as cell defense mechanisms are downregulated in microgravity, allowing *P. phymatum* to direct its energy towards cell proliferation. This observation is in line with other studies conducted in *Escherichia coli*, *Deinococcus radiodurans*, *Pseudomonas aeruginosa* or *Bacillus subtilis*, where exposure to real or simulated microgravity induced an oxidative and osmotic stress response, but did not impact their viability^45,46^. Our preliminary data also suggest that *P. phymatum* cells grown in s0-g are slightly more resistant to oxidative stress than the ones in 1 g (Supplementary Fig. 2). These results indicate a generalized effect induced in microgravity that could be due to an accumulation of cellular waste in the proximity of the cells^47,48^. In fact molecules in this microenvironment can only be exchanged through Brownian motion (diffusion) because of the lack of shear forces^45,46,49^. It is also interesting to note that most of the genes coding for *P. phymatum* type VI secretion system 3 (T6SS-3) were upregulated in cells grown in simulated microgravity. However, *P. phymatum* second T6SS (T6SS-b) showed the opposite behavior and was downregulated when *P. phymatum* was grown in artificial weightlessness (Supplementary Table 1)^36^. As both *P. phymatum* T6SS have been shown to be important for interbacterial competition in vitro and *in planta*, prospective competition experiments performed in 1 g and s0-g might clarify whether exposure to artificial microgravity has an influence *on P. phymatum* ability to outcompete against other soil bacteria^37^. Moreover, genes involved in motility and chemotaxis were found to be downregulated. All the genes involved in flagella formation, for example, showed a significantly lower expression in cells grown in s0-g compared to 1 g. Downregulation of genes involved in motility has also been observed in other transcriptomic studies conducted in *E*. *coli* or *Salmonella typhimurium*, in both true and simulated weightlessness. In both cases this effect was explained by an upregulation of the transcriptional regulatory gene *hfq*^50^. We did not observe any change in Bphy_1711 (*hfq)* expression in *P. phymatum* grown in s0-g compared to 1 g. The RNA-seq data also revealed that genes coding for proteins that are key for ensuring bacterial cells shape, such as the rod shape determining proteins MreC and RodA, are downregulated in *P. phymatum* grown in s0-g compared to 1 g (Supplementary Table 1). Another strongly downregulated gene cluster appeared to code for a hydroxamate siderophore. This *P. phymatum* gene cluster was previously predicted to code for a siderophore named phymabactin^38,51^, we show here for the first time that it indeed codes for the only siderophore produced by *P. phymatum*. Deletion of the NRPS encoding gene *phmJ* and the acetyltransferase coding gene *phmK* resulted in a mutant unable to synthetize siderophores (Fig. 5). In addition, we confirmed that less siderophores were produced in space-like conditions relative to 1 g (Fig. 3). Yet, the *P. phymatum phmJK* deletion mutant displayed a similar growth profile as the wild-type strain when cultured in minimal media and was able to grow in iron limited conditions (Supplementary Fig. 3), suggesting that *P. phymatum* has other iron importers that permit its survival even in absence of phymabactin. In fact, a bioinformatic search revealed that *P. phymatum* also possesses the genes necessary to produce the Ftr siderophore-alternative iron uptake system (Bphy_1954 – Bphy_1957)^52^. Interestingly, these genes were also downregulated in cells grown in s0-g compared to 1 g according to the RNA-seq data. A possible explanation for the reduced iron uptake in microgravity is that the already stressed cells limit their iron import to minimize the level of oxidative stress. This effect has already been observed in transcriptomic analyses conducted in *E. coli* strain Nissle 1917 grown in simulated microgravity. In full agreement with our data, this study reported a downregulation in iron transporters and an upregulation in genes involved in the cellular response against oxidative stress and suggested that microbial cells in s0-g displayed a limited iron uptake as a protection against hydroxyl radicals^53^. Finally, the downregulation of a common good such as phymabactin may have an impact in the context of a diverse microbial community in space-like conditions by affecting the community structure.

A concern that must be addressed when designing space bases is that maintaining a full nitrogen balance through N_2_ production will be required to ensure a stable atmospheric pressure in the closed habitat and compensate gases leaks^54^. In addition, it is possible that plants in extraterrestrial habitats will be grown using a nutrient solution prepared from nitrogen rich human wastes instead of a fully synthetic growth medium^54^. If it is the case, mineral nitrogen in the plants growth medium has to be controlled, as excessive amount could be detrimental for N_2_ fixation^54,55^. For instance, denitrification of the wastes coupled with the utilization of rhizobia could be the solution for ensuring a good amount of mineral nitrogen available for plants without fearing the depletion of gaseous N_2_ for the crew^54,55^.

To summarize, *P. phymatum* cells exposed to simulated microgravity displayed drastic changes in gene expression when grown in s0-g. A gene cluster that was strongly downregulated in s0-g was shown to encode the siderophore phymabactin. The importance of this siderophore in *P. phymatum* physiology and in its adaptation to low gravity needs to be investigated further. Importantly, the fact that this promiscuous and stress resistant beta-rhizobial strain *P. phymatum* grew in simulated microgravity as well as in normal gravity suggests that it also adapts very well to space-like conditions, makes it an excellent candidate for an inoculant for crops grown in future lunar or Martian colonies.

## Methods

### Bacterial strains, media and cultivation

Bacterial strains, plasmids and primers as well as the antibiotics used in this study are listed in Supplementary Tables 2 and 3. *E. coli* was grown in Luria-Bertani (LB) rich medium. *P. phymatum* was cultured in a modified version of this medium without salt (LB-NaCl) or in AB minimal medium supplemented with 15 mM succinate (Sigma-Aldrich, St. Louis, MO, USA) as the carbon source^56^. *P. phymatum* cells were grown in 70 mL flasks (Sarstedt, Germany) at 28 °C and cultured in simulated microgravity using a random positioning machine (RPM) at a rotation rate of 60°/s until towards the end of the exponential phase (OD_600_ of 0.7). The RPM was built at the Lucerne School of Engineering and Architecture (HSLU) and was used to induce simulated microgravity throughout the study. RPMs were introduced decades ago as a ground-based tool for simulating microgravity, alongside other techniques implemented in instruments like Clinostats and Rotating Wall Vessels^29^. The fundamental concept behind the RPMs involves the slow and random rotation of biological samples (Supplementary Video 1). By controlling the movement to ensure that the gravity vector continually repositions itself in all directions for an equal duration, it effectively averages out gravity to zero from a mathematical standpoint^57^. In addition, a specific orientation of the gravity vector must be maintained for a minimal amount of time to enable biological systems, such as cells, to perceive gravity. With a sufficiently rapid rotation speed, typically around ten revolutions per minute, and a well-distributed gravity vector, cells experience an environment closely resembling microgravity. This phenomenon has been substantiated through numerous experiments conducted on Earth and in actual microgravity conditions in space^58^. The 1 g control cultures were grown on a shaker at 60 rpm in the same incubator. For each biological replicate, an overnight culture derived from one *P. phymatum* single colony was washed and inoculated both in s0-g and in 1 g.

### RNA-sequencing and data processing

Three biological replicates of *P. phymatum* cells were grown in s0-g and in 1 g until towards the end of the exponential growth phase. Fifty mL of each culture was then pelleted, stop solution was added and the samples were flash-frozen in liquid nitrogen and stored at −80 °C. RNA extraction was performed using a modified version of the hot acid phenol method^41^. Genomic DNA (gDNA) removal was performed with RQ1 DNase treatment (PROMEGA, Madison WI, USA), and verification of the RNA quality was evaluated using the Agilent RNA ScreenTape System (Agilent, Santa Clara, CA, USA)^41^. One hundred fifty ng of high-quality RNA was used for cDNA synthesis using the Ovation® RNA-seq System V2 (TECAN, Switzerland) for libraries preparation and purification. The cDNA libraries were quantified by capillary electrophoresis with the Agilent D1000 Screen Tape System before performing Illumina single-end sequencing with the Illumina Novaseq 6000 (100 bp single reads; Illumina, San Diego, CA, USA). The reads were mapped according to the *P. phymatum* STM815^T^ genome (accession numbers: chromosome 1: NC_010622, chromosome 2: NC_010623, plasmid pBPHY01: NC_010625, plasmid pBPHY02: NC_010627) with CLC Genomics Workbench (version 22.0.2, QIAGEN CLC bio, Aarhus, Denmark), allowing up to two mismatches per read. The statistical analysis for the differential expression of the uniquely mapped reads was performed using DESeq 2 package in R v4.2.0^59^. A fold-change threshold of log_2_ ≥ 1or ≤ −1 was applied with a *p* ≤ 0.01 to consider a gene as differentially expressed. The functional classification of the differentially expressed genes was assessed using eggNOG-Mapper v2 5.0 (Computational Biology Group, EMBL, Heidelberg, Germany) and the statistical analysis was performed through Fisher test^60,61^.

### Construction of *P. phymatum* STM815^T^ mutant strains

The gDNA of *P. phymatum* STM815^T^ wild-type strain was isolated through the GenEluteTM Bacterial Genome Dna kit (Sigma-Aldrich, St. Louis, MO, USA) whereas plasmid DNA from *E. coli* strains was extracted using the QIAprep Spin Miniprep kit (Qiagen, Hilden, Germany). A *phmJK* deletional mutant was constructed by cloning the flanking upstream (528 bp) and downstream (562 bp), in a suicide plasmid (see below). *P. phymatum* gDNA was used as a template to amplify the upstream fragment with the primers Bphy_4038_up_F_EcoRI and Bphy_4038_up_R_XhoI, while the primers Bphy_4038_dn_F_NdeI and Bphy_4038_dn_R_EcoRI were used for the downstream fragment. A trimethoprim cassette (Trim) was cut out of a p34E-Tp-Tr plasmid using *Nde*I and *Xho*I restriction enzymes. The downstream fragment was digested with *Nde*I and ligated with the Trim cassette before PCR amplification using Bphy_4038_dn_R_EcoRI and the Trim_stop_R_XhoI primers before digestion with *Xho*I. The upstream fragment was digested with *Xho*I before ligation to the downstream fragment. The DNA was then amplified with the Bphy_4038_up_F_EcoRI and Bphy_4038_dn_R_EcoRI primers and inserted into a pSHAFT2 suicide vector. *E. coli* cc118-λ pir containing the resulting plasmid was afterwards used in a triparental mating with *P. phymatum* wild-type and the helper strain *E. coli* pRK2013^62,63^. Transconjugants were isolated through double purification on plates containing Trim and chloramphenicol, and the correct mutant strain was confirmed using the Bphy_4038_veri_F and Bphy_4038_veri_R primers. *P. phymatum* was confirmed by amplifying *nifH* with the Bphy_7808_nifH_F and Bphy_7808_NifH_R primers, and the absence of *E. coli* was confirmed with the rpoD_F and rpoD_R primers.

### Quantification of the siderophore production in s0-g

Siderophore-producing ability was measured quantitatively through a liquid CAS assay^64^. For this, 70 mL of PIPES (Sigma-Aldrich, St. Louis, MO, USA) buffered ABS medium were inoculated with *P. phymatum* cells (OD_600_ of 0.05) and incubated in 1 g and s0-g at 28 °C for 17 h. Bacterial cultures were then centrifuged for 10 min at 10,000 rpm, cell pellets were discarded, and the supernatant was filter sterilized using a syringe and a 0.2 µm-pore filter (Filtropur 0.2, Sarstedt, Germany). Five-hundred µL of each culture’s supernatant was mixed with 0.5 mL of previously prepared CAS reagent (for 100 mL of dye: 0.06 g CAS, 0.073 g hexadecyltrimethylammonium bromide (HDTMA), 0.027 g FeCl_3_-6H_2_O (Sigma-Aldrich, St. Louis, MO, USA)) prepared according to the standard recipe^65^. The mixture was incubated for 20 min at room temperature and the color change of the medium from deep blue to orange, due to the chelating action of the siderophore that removes the iron from the CAS/HDTMA of the dye, density was measured at 630 nm with the Ultrospec 2100 *pro* spectrophotometer (Amersham Biosciences, Amersham, United Kingdom). The siderophore production was measured in three biological replicates and expressed as the CAS activity relative to the reference according to the following formula^66^:

$${CAS}\,{activity}=\frac{\frac{\left({Ar}-{As}\right)x100}{{Ar}}}{{OD}600}$$

With Ar being the absorbance of the reference (CAS solution with fresh medium) and As the absorbance of sample at OD_630_ (CAS solution and culture supernatant). The OD_600_ is the absorbance at an OD_600_ of the culture prior to starting the quantification.

### Qualitative detection of siderophore production

A modified version of the CAS-agar assay was used to assess *P. phymatum* cells production of siderophores in three biological replicates^64^. AB medium and 15 mM succinic acid as a carbon source was used as a base, before addition of PIPES buffer and CAS dye. Washed overnight cultures of *P. phymatum* wild-type strain and *phmJK* deletion mutant were then normalized to an OD_600_ of 0.5 and spotted on CAS-agar plates before incubation for 48 h at 28 °C.

### Quantitative detection of siderophore production

To determine quantitatively the siderophore production of *P. phymatum* wild-type and the *phmJK* mutant, glass tubes filled with three mL of PIPES buffered ABS were inoculated to an OD_600_ of 0.05 with *phmJK* and *P. phymatum* wild-type cells, and incubated overnight at 180 rpm and 28 °C. Next, the cells were pelleted, and the supernatant was mixed with CAS dye for determination of the CAS activity as described above.

### Statistical information

Fischer tests on genes belonging to eggNOG categories were conducted using Microsoft Excel 2023 Student’s *t* tests were performed for all the phenotypical assays (quantitative siderophore production, RFU) using GraphPad Prism 7.00.

### Supplementary information

Supplemental material

Supplementary table 1

Supplementary video 1

## Data Availability

The RNA-sequencing raw data files obtained in this work are accessible through the GEO series accession number GSE255155.
