# Supplementary material for: RNA-seq analysis in simulated microgravity unveils down-regulation of the beta-rhizobial siderophore phymabactin
Source: NPJ Microgravity. 2024 Apr 3;10:44. doi: 10.1038/s41526-024-00391-7 (PMC10991261; doi:10.1038/s41526-024-00391-7)
Supplement: Supplementary file 1 — Supplemental material [file 41526_2024_391_MOESM1_ESM.docx]

# **RNA-seq analysis in simulated microgravity unveils downregulation of the beta-rhizobial siderophore phymabactin**

Golaz *et al*.,

**Supplementary information**

Supplementary Figures 1 to 3

Supplementary Tables 2 to 4

Supplementary references

**Supplementary information**

**Supplementary Figures**

**
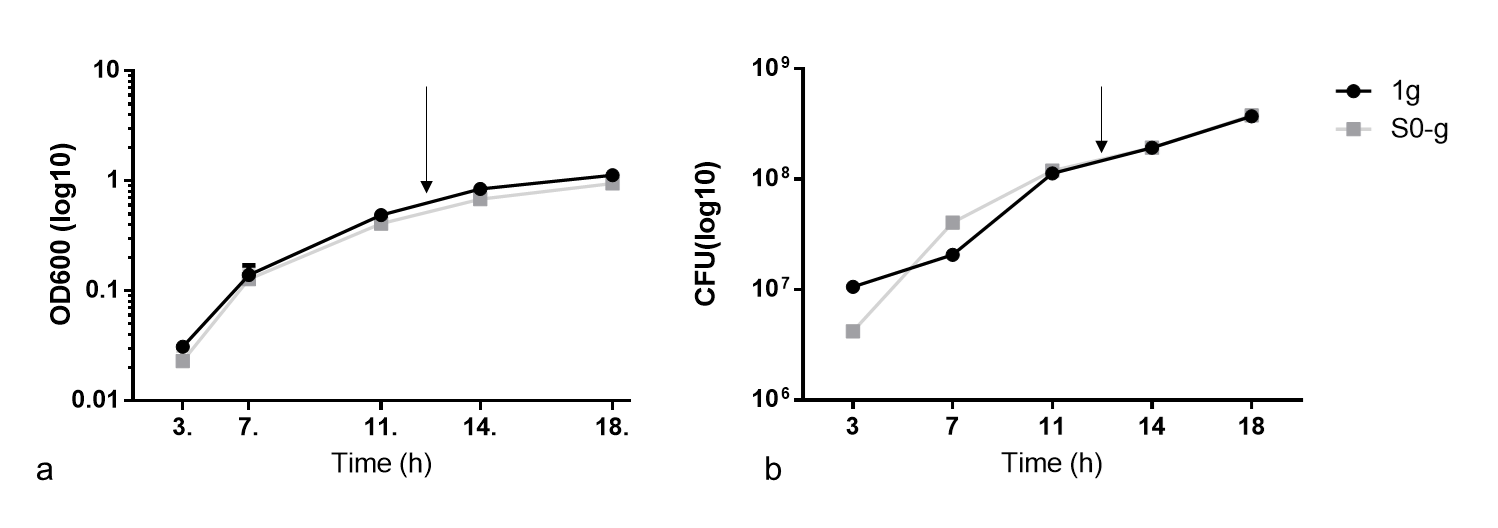
**

**Supplementary Figure 1. Growth profile of *P. phymatum* wild-type grown in 1g and in s0-g according to its absorbance at OD_600_ (a) or the counted colony forming units (CFU) (b).** No difference in growth was observed between the cells grown in 1g and s0-g. Three (n=3) biological replicates were performed. The standard deviation is indicated as bars. The arrows indicate the sampling point.


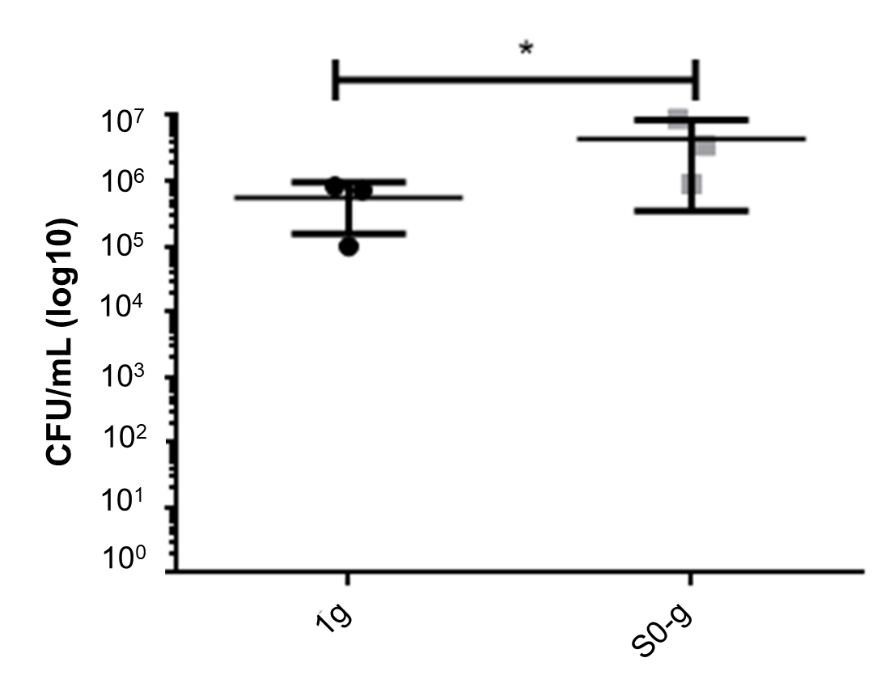


**Supplementary Figure 2. Colony forming units (CFU) of *P. phymatum* wild-type cells subjected to H_2_O_2_ induced oxidative stress in 1g and s0-g.** The cells grown in s0-g were more resistant to oxidative stress than the ones cultivated in 1g (*: *p*-value < 0.05). The standard deviation is shown as bars, and three (n = 3) biological replicates were tested. Analysis was performed using Student t-test.


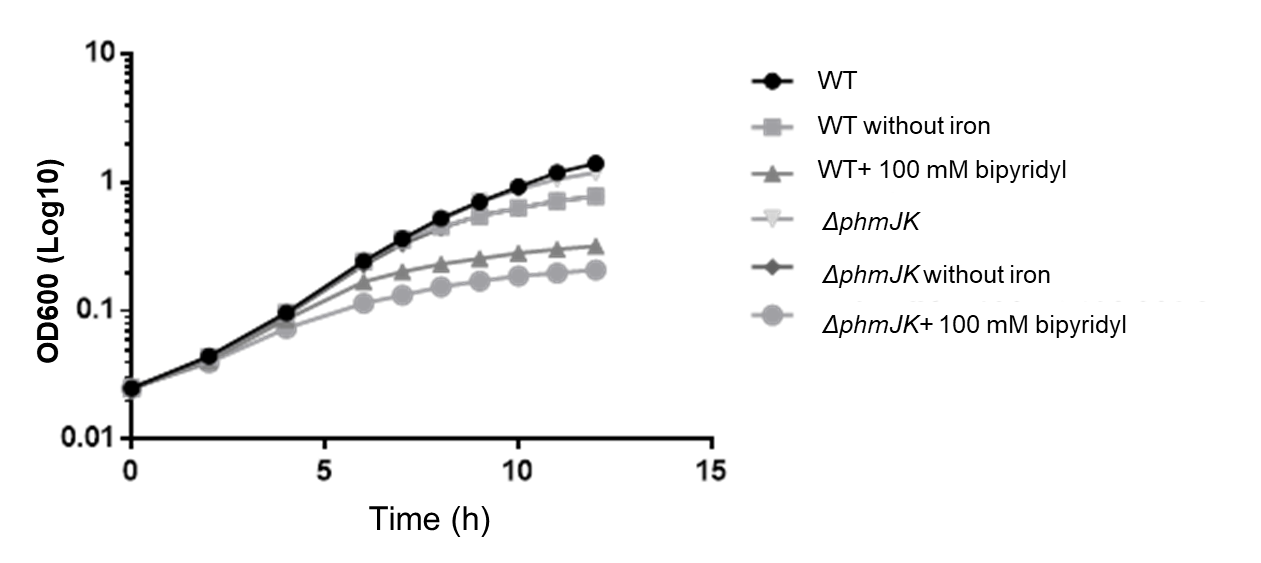


**Supplementary Figure 3. Growth profile of *P. phymatum* wild-type (WT) and *phmJK* mutant (Δ*phmJK*) in ABS minimal medium, ABS minimal medium prepared without iron and ABS minimal medium without iron supplemented with 100 mM of the iron chelator bipyridyl.** The wild-type and the mutant strain grown without iron grew alike. Data are mean ± standard deviation of three (n=3) biological replicates.

**Supplementary Tables**

**Supplementary Table 1:** see separate PDF file

**Supplementary Table 2.** Homologies of all the genes constituting the phymabactin gene cluster (in *P. phymatum* STM815) with the ornibactin gene cluster (in *B. cenocepacia* H111) and the malleobactin gene cluster (in *B. pseudomallei* 1026b).

|  | ***P. phymatum* STM815** | | ***B. cenocepacia* H111** | | | ***B. pseudomallei* 1026b** | | |
| --- | --- | --- | --- | --- | --- | --- | --- | --- |
| ID in *phm* | gene name | similarity (a.a) | gene name | Similarity (a.a) | E. value | gene name | similarity (a.a) | E. value |
| Bphy_ 4047 | *phmS* | 100% | *orbS* | 69.19% | 4,00E-92 | *mbaF* | 67.57% | 1,00E-93 |
| Bphy_4046 | *phmH* | 100% | *orbH* | 74.36% | 4,00E-41 | *mbaG* | 90.74% | 2,00E-38 |
| Bphy_4045 | *phmG* | 100% | *orbG* | 81.99% | 0.0 | *mbaH* | 82.72% | 0.0 |
| Bphy_4044 | *phmC* | 100% | *orbC* | 75.37% | 2,00E-154 | *mbaI* | 79.09% | 2,00E-156 |
| Bphy_4043 | *phmD* | 100% | *orbD* | 66.95% | 0.0 | *mbaJ* | 67.71% | 0.0 |
| Bphy_4042 | *phmF* | 100% | *orbF* | 55.77% | 9,00E-94 | *mbaK* | 55.91% | 5,00E-85 |
| Bphy_4041 | *phmB* | 100% | *orbB* | 62.93% | 8,00E-135 | *mbaL* | 63.75% | 1,00E-134 |
|  |  |  |  |  |  | *mbaM* |  |  |
| Bphy_4040 | *phmE* | 100% | *orbE* | 77.03% | 0.0 | *mbaN* | 75.48% | 0.0 |
| Bphy_4039 | *phmI* | 100% | *orbI* | 62.58% | 0.0 | *mbaA* | 61.46% | 0.0 |
| Bphy_4038 | *phmJ* | 100% | *orbJ* | 58.79% | 0.0 | *mbaB* | 59.54% | 0.0 |
| Bphy_4037 | *phmK* | 100% | *orbK* | 45.57% | 2,00E-75 |  |  |  |
| Bphy_4036 | *pvdA* | 100% | *pvdA* | 67.45% | 0.0 | *mbaC* | 68.74% | 0.0 |
|  |  |  | *orbA* |  |  | *mbaD* |  |  |
|  |  |  | *pvdF* |  |  | *mbaE* |  |  |
| Bphy_4035 | *phmL* | 100% | *orbL* | 60.40% | 3,00E-140 |  |  |  |

The analysis was performed using the Burkholderia database (<https://www.burkholderia.com/>) and BlastP (https://blast.ncbi.nlm.nih.gov/Blast.cgi). For each cluster, there is the name of the present genes, the similarity with the orthologue gene in *P. phymatum* and the E. value obtained from the analysis. The gaps indicate that this gene does not have an orthologue that can be compared to any *P. phymatum* genes.

**Supplementary Table 3.** List of strains and plasmids used during the study.

| Strain or plasmid | Description | Reference |
| --- | --- | --- |
| ***E. coli*** |  |  |
| Cc18λ-pir | Δ(*ara-leu*) *araD ΔlacX74 galE galK phoA20 thil rpsE rpoB argE(Am) recAlλpir*, Strep^R^ | (Herrero et al., 1990) |
| Top10 | *ΔlacX74 araΔ139Δ (ara-leu*), Amp^R^ | Invitrogen |
| ***P. phymatum*** |  |  |
| STM 815 | Wild-type | (Moulin et al., 2001) |
| Δ*phmJ*K | Deletion mutant for the *phm*J and *phm*K genes, Trim^R^ | This study |
| p*phm* | *phm*-*gfp* reporter fusion strain, Kan^R^ | This study |
| pPROBE | Wild-type strain carrying the pPROBE-NT plasmid, Kan^R^ | This study |
| **Plasmids** |  |  |
| pRK2013 | Helper plasmid, Kan^R^ | (Figurski and Helinski, 1979) |
| pSHAFT2 | Suicide plasmid, Cm^R^ | (Shastri et al., 2017) |
| pSHAFT2-*phmJK* | Suicide plasmid containing 1969 bps external fragment of Bphy_4038 for mutagenesis, Cm^R^, Trim^R^ | This study |
| pPROBE-NT | Broad-hoast-range promotor-probe vector, Kan^R^ | (Liu et al., 2020) |
| pPROBE-*phm* | pPROBE with Bphy_4047 promoter probe vector, Kan^R^ | This study |

**Supplementary Table 4.** List of primers used during this study

| Oligonucleotide | Sequence | Reference |
| --- | --- | --- |
| Bphy_4038_up_F_EcoRI | CGCGgaattcGAACTCTTCTTGCGCGCG | This study |
| Bphy_4038_up_R_XhoI | CGCGctcgagTCCTGTCGTTCCTTATTGCG | This study |
| Bphy_4038_dn_F_NdeI | GCGCcatatgATATCGTCCCGTGTGAACTC | This study |
| Bphy_4038_dn_R_EcoRI | GCGCgaattcAACGTGAAGCGATGTCCGAA | This study |
| Bphy_4038_veri_F | GCGCCTCGTTCATCCATG | This study |
| Bphy_4038_veri_R | GTCGTGATCGTGCGAGAC | This study |
| Trim_stop_R_NdeI | GCGCCATATGGTAGATATGACGACAGGA | This study |
| pSHAFT_F | CTTCAGCTGATGTGTGATAACATACT | (Lardi et al., 2017) |
| Bphy_7808_F | GGCGTGGACTATGTGTCGTA | (Lardi et al., 2017) |
| Bphy_7808_R | GATGCCCTTCGAGATGTTGT | (Lardi et al., 2017; Liu et al., 2020) |
| rpoD_F | CAATGCTCCGTTGCTGAATA | (Lardi et al., 2020) |
| rpoD_R | TTCATCTTCGTCATCGTCCA | (Lardi et al., 2020) |

**Supplementary References**

1. Figurski, D.H. and Helinski, D.R., Replication of an origin-containing derivative of plasmid RK2 dependent on a plasmid function provided in trans. *Proceedings of the National Academy of Sciences of the United States of America*, 1979, vol. 76, pp. 1648–1652. doi:10.1073/pnas.76.4.1648.
2. Herrero, M., Lorenzo, V. de and Timmis, K.N., Transposon vectors containing non-antibiotic resistance selection markers for cloning and stable chromosomal insertion of foreign genes in gram-negative bacteria. *Journal of bacteriology*, 1990, vol. 172, pp. 6557–6567. doi:10.1128/jb.172.11.6557-6567.1990.
3. Lardi, M., Liu, Y., Hug, S., Bolzan de Campos, S., Eberl, L. and Pessi, G., *Paraburkholderia phymatum* STM815 σ54 Controls Utilization of Dicarboxylates, Motility, and T6SS-b Expression. *Nitrogen*, 2020, vol. 1, pp. 81–98. doi:10.3390/nitrogen1020008.
4. Lardi, M., Liu, Y., Purtschert, G., Bolzan de Campos, S. and Pessi, G., Transcriptome Analysis of *Paraburkholderia phymatum* under Nitrogen Starvation and during Symbiosis with Phaseolus Vulgaris. *Genes*, 2017, vol. 8. doi:10.3390/genes8120389.
5. Liu, Y., Bellich, B., Hug, S., Eberl, L., Cescutti, P. and Pessi, G., The Exopolysaccharide Cepacian Plays a Role in the Establishment of the *Paraburkholderia phymatum* - *Phaseolus vulgaris* Symbiosis. *Frontiers in microbiology*, 2020, vol. 11, p. 1600. doi:10.3389/fmicb.2020.01600.
6. Moulin, L., Munive, A., Dreyfus, B. and Boivin-Masson, C., Nodulation of legumes by members of the beta-subclass of Proteobacteria. *Nature*, 2001, vol. 411, pp. 948–950. doi:10.1038/35082070.
7. Shastri, S., Spiewak, H.L., Sofoluwe, A., Eidsvaag, V.A., Asghar, A.H., Pereira, T., Bull, E.H., Butt, A.T. and Thomas, M.S., An efficient system for the generation of marked genetic mutants in members of the genus *Burkholderia*. *Plasmid*, 2017, vol. 89, pp. 49–56. doi:10.1016/j.plasmid.2016.11.002.
