# Supplementary material for: RNA-seq analysis in simulated microgravity unveils down-regulation of the beta-rhizobial siderophore phymabactin
Source: NPJ Microgravity. 2024 Apr 3;10:44. doi: 10.1038/s41526-024-00391-7 (PMC10991261; doi:10.1038/s41526-024-00391-7)
Supplement: Supplementary file 2 — Supplementary table 1 [file 41526_2024_391_MOESM2_ESM.pdf]

Table S1: List of *P. phymatum* DEG, the logarithm (base two) of the fold changes in expression and the p-values in microgravity (s0-g) and terrestrial gravity (1g)

ID[1]	g0/g1 (log2FoldChange)	pvalue	name	old_locus_tag[2]	description[1]
BPHY_RS00065	-2,143	1,07E-06	dbpA	Bphy_0013	ATP-dependent RNA helicase DbpA
BPHY_RS00100	-6,364	0,000745		Bphy_0020	DUF4148 domain-containing protein
BPHY_RS00110	-1,591	0,004034	tssF	Bphy_0022	type VI secretion system baseplate subunit TssF
BPHY_RS00145	1,207	0,00127		Bphy_0030	phosphoenolpyruvate carboxykinase (GTP)
BPHY_RS00150	1,147	0,0008		Bphy_0031	LysR family transcriptional regulator
BPHY_RS00185	1,060	0,005001	htpX	Bphy_0038	zinc metalloprotease HtpX
BPHY_RS00225	-1,609	0,003633	queD	Bphy_0045	6-carboxytetrahydropterin synthase QueD
BPHY_RS00240	-1,579	0,001202	rodA	Bphy_0048	rod shape-determining protein RodA
BPHY_RS00245	-1,430	9,14E-06	mrdA	Bphy_0049	penicillin-binding protein 2
BPHY_RS00255	-1,504	0,000201	mreC	Bphy_0051	rod shape-determining protein MreC
BPHY_RS00260	-1,194	0,008779		Bphy_0052	rod shape-determining protein
BPHY_RS00335	1,561	0,000381		Bphy_0068	response regulator transcription factor
BPHY_RS00340	2,263	5,03E-08	hslU	Bphy_0069	ATP-dependent protease ATPase subunit HslU
BPHY_RS00345	1,994	6,9E-06	hslV	Bphy_0070	ATP-dependent protease subunit HslV
BPHY_RS00360	-1,283	0,004287		Bphy_0073	class I SAM-dependent rRNA methyltransferase
BPHY_RS00380	-1,571	0,000107		Bphy_0077	lauroyl acyltransferase
BPHY_RS00415	-1,814	0,000215		Bphy_0084	MFS transporter
BPHY_RS00420	-3,757	0,008185		Bphy_0085	hypothetical protein
BPHY_RS00465	1,294	0,001384		Bphy_0094	y proteinmolybdopterin-binding subunit
BPHY_RS00470	1,620	5,85E-06		Bphy_0095	(2Fe-2S)-binding protein
BPHY_RS00475	-1,518	0,000588		Bphy_0096	amino acid permease
BPHY_RS00485	1,640	4,42E-06		Bphy_0098	histidine kinase
BPHY_RS00490	-1,240	0,008653		Bphy_0099	ferredoxin--NADP reductase
BPHY_RS00500	1,456	0,007585		Bphy_0101	aldose 1-epimerase
BPHY_RS00505	1,694	1,6E-05		Bphy_0102	endonuclease/exonuclease/phosphatase familyprotein
BPHY_RS00510	1,142	0,005234		Bphy_0103	hypothetical protein
BPHY_RS00575	-1,508	0,002669		Bphy_0117	cation diffusion facilitator family transporter
BPHY_RS00615	-1,376	0,00357		Bphy_0125	BON domain-containing protein
BPHY_RS00665	1,718	2,94E-06		Bphy_0134	response regulator
BPHY_RS00690	-1,107	0,003693		Bphy_0139	Na+/H+ antiporter
BPHY_RS00725	1,997	4,78E-06		Bphy_0146	hypothetical protein
BPHY_RS00750	-1,813	0,004113		Bphy_0151	phosphodiesterase
BPHY_RS00840	-1,010	0,007813		Bphy_0169	sulfate ABC transporter substrate-bindingprotein
BPHY_RS00910	1,426	8,23E-05		Bphy_0183	ABC transporter substrate-binding protein
BPHY_RS00940	1,426	0,000281		Bphy_0189	DUF1840 domain-containing protein
BPHY_RS00960	3,042	2,86E-06		Bphy_0192	DUF3567 domain-containing protein
BPHY_RS00965	3,451	1,06E-25	pcaD	Bphy_0193	3-oxoadipate enol-lactonase
BPHY_RS00995	1,075	0,002166		Bphy_0199	hypothetical protein
BPHY_RS01060	-1,827	5,13E-06		Bphy_0212	D-alanyl-D-alanine carboxypeptidase

BPHY_RS01105	1,084	0,002909		Bphy_0221	ABC transporter substrate-binding protein
BPHY_RS01125	-2,212	5,91E-11		Bphy_0225	dicarboxylate/amino acid:cation symporter
BPHY_RS01130	-2,042	1,64E-07		Bphy_0226	sensor histidine kinase
BPHY_RS01155	2,169	5,13E-11		Bphy_0231	hypothetical protein
BPHY_RS01160	1,463	0,000111		Bphy_0232	hypothetical protein
BPHY_RS01235	-1,951	4,45E-05		Bphy_0247	hypothetical protein
BPHY_RS01240	-1,948	0,006104		Bphy_0248	hypothetical protein
BPHY_RS01305	-1,218	0,009162	moeB	Bphy_0263	molybdopterin-synthase adenylyltransferase MoeB
BPHY_RS01335	-1,915	7,2E-05		Bphy_0270	NAD(P)-dependent glycerol-3-phosphatedehydrogenase
BPHY_RS01405	1,790	2,68E-08		Bphy_0285	COX15/CtaA family protein
BPHY_RS01420	1,697	0,001792		Bphy_0288	YciI family protein
BPHY_RS01430	-1,979	4,04E-06		Bphy_0290	MetQ/NlpA family lipoprotein
BPHY_RS01460	1,998	2,02E-06		Bphy_0296	N-acetylglucosamine-specific PTS transportersubunit IIBC
BPHY_RS01470	3,527	7,28E-24	cydB	Bphy_0298	cytochrome d ubiquinol oxidase subunit II
BPHY_RS01475	3,099	9,5E-14		Bphy_0299	cytochrome ubiquinol oxidase subunit I
BPHY_RS01480	2,470	1,1E-09		Bphy_0300	hypothetical protein
BPHY_RS01485	1,323	0,000146	rpoH	Bphy_0301	RNA polymerase sigma factor RpoH
BPHY_RS01535	1,065	0,008599		Bphy_0311	YfhL family 4Fe-4S dicluster ferredoxin
BPHY_RS01540	-1,197	0,002703		Bphy_0312	histidinol-phosphate transaminase
BPHY_RS01545	-1,386	0,003646	pth	Bphy_0313	aminoacyl-tRNA hydrolase
BPHY_RS01550	-1,854	0,000303		Bphy_0314	50S ribosomal protein L25/general stress proteinCtc
BPHY_RS01555	-2,071	2,69E-05		Bphy_0315	ribose-phosphate pyrophosphokinase
BPHY_RS01590	1,975	2,19E-10		Bphy_0321	LON peptidase substrate-bindingdomain-containing protein
BPHY_RS01595	1,266	0,001076	rapZ	Bphy_0322	RNase adapter RapZ
BPHY_RS01615	3,098	2,88E-21	raiA	Bphy_0325	ribosome-associated translation inhibitor RaiA
BPHY_RS01675	-2,792	3,23E-08		Bphy_0337	PepSY domain-containing protein
BPHY_RS01680	-1,379	0,008084		Bphy_0338	AI-2E family transporter
BPHY_RS01720	1,723	0,000675		Bphy_0346	c-type cytochrome
BPHY_RS01730	2,001	0,002001		Bphy_0348	cytochrome c oxidase subunit II
BPHY_RS01735	1,987	0,003558		Bphy_0349	hypothetical protein
BPHY_RS01740	2,192	2,21E-07		Bphy_0350	cytochrome c
BPHY_RS01745	1,507	0,000345		Bphy_0351	c-type cytochrome
BPHY_RS01785	1,264	0,001021		Bphy_0359	CHRD domain-containing protein
BPHY_RS01810	1,394	5,42E-05		Bphy_0364	amino acid deaminase
BPHY_RS01815	1,182	0,006077		Bphy_0365	MurR/RpiR family transcriptional regulator
BPHY_RS01865	1,301	0,002801		Bphy_0375	39S ribosomal protein L45
BPHY_RS01945	-1,064	0,009185		Bphy_0391	EAL domain-containing protein
BPHY_RS01965	-1,213	0,004919		Bphy_0394	nucleotidyltransferase family protein
BPHY_RS01980	-1,051	0,008907		Bphy_0397	peptidylprolyl isomerase
BPHY_RS02015	-1,791	0,001547	gmhB	Bphy_0404	D-glycero-beta-D-manno-heptose 1,7-bisphosphate7-phosphatase
BPHY_RS02020	-1,603	0,000687		Bphy_0405	glycine--tRNA ligase subunit beta
BPHY_RS02040	-2,704	0,001059		Bphy_0409	gamma-glutamylcyclotransferase
BPHY_RS02045	-2,534	2,64E-07	ybeY	Bphy_0410	rRNA maturation RNase YbeY

BPHY_RS02050	-1,432	0,00374		Bphy_0411	PhoH family protein
BPHY_RS02115	1,845	2,49E-06		Bphy_0424	ferritin-like domain-containing protein
BPHY_RS02125	-1,272	0,003344		Bphy_0426	DEAD/DEAH box helicase
BPHY_RS02215	-1,057	0,002347	recX	Bphy_0441	recombination regulator RecX
BPHY_RS02235	-1,695	0,000937		Bphy_0445	TerC family protein
BPHY_RS02320	-1,184	0,007737		Bphy_0462	phosphoribosylaminoimidazolesuccinocarboxamidesynthase
BPHY_RS02325	-1,674	0,006073	purE	Bphy_0463	5-(carboxyamino)imidazole ribonucleotide mutase
BPHY_RS02330	-1,454	0,000166		Bphy_0464	5-(carboxyamino)imidazole ribonucleotidesynthase
BPHY_RS02335	-1,675	0,000426		Bphy_0465	threonylcarbamoyl-AMP synthase
BPHY_RS02340	-2,021	0,000605		Bphy_0466	SGNH/GDSL hydrolase family protein
BPHY_RS02345	-2,014	0,005462		Bphy_0467	sterol desaturase family protein
BPHY_RS02400	-1,384	0,002021		Bphy_0478	efflux RND transporter periplasmic adaptorsubunit
BPHY_RS02405	-1,640	2,94E-05		Bphy_0479	efflux RND transporter permease subunit
BPHY_RS02410	-1,162	0,004341		Bphy_0480	efflux transporter outer membrane subunit
BPHY_RS02435	-1,419	0,006221		Bphy_0485	metal ABC transporter permease
BPHY_RS02440	-1,646	0,000246		Bphy_0486	ABC transporter ATP-binding protein
BPHY_RS02485	-3,213	0,00011		Bphy_0495	tetratricopeptide repeat protein
BPHY_RS02570	1,121	0,001902	msrA	Bphy_0511	peptide-methionine (S)-S-oxide reductase MsrA
BPHY_RS02575	1,082	0,006577		Bphy_0512	DUF72 domain-containing protein
BPHY_RS02580	3,178	8,71E-19		Bphy_0513	cyclopropane-fatty-acyl-phospholipid synthasefamily protein
BPHY_RS02585	1,148	0,000157	pdxH	Bphy_0514	pyridoxamine 5'-phosphate oxidase
BPHY_RS02595	1,218	0,00251	trxA	Bphy_0516	thioredoxin
BPHY_RS02600	1,319	7,31E-05		Bphy_0517	pirin family protein
BPHY_RS02640	-1,932	0,000245	ybaK	Bphy_0525	Cys-tRNA(Pro) deacylase
BPHY_RS02645	-1,617	0,000337	plsY	Bphy_0526	glycerol-3-phosphate 1-O-acyltransferase PlsY
BPHY_RS02650	-1,148	0,007231		Bphy_0527	YajQ family cyclic di-GMP-binding protein
BPHY_RS02655	-3,336	0,000156	murB	Bphy_0528	UDP-N-acetylmuramate dehydrogenase
BPHY_RS02670	-1,965	0,000583	rpsT	Bphy_0531	30S ribosomal protein S20
BPHY_RS02750	-1,680	0,001259	mdtD	Bphy_0547	multidrug transporter subunit MdtD
BPHY_RS02815	1,406	0,000912	fusA	Bphy_0561	elongation factor G
BPHY_RS02825	-1,538	0,008356			hypothetical protein
BPHY_RS02830	-1,604	0,001367		Bphy_0564	pseudouridine synthase
BPHY_RS02855	1,568	5,58E-05	clpA	Bphy_0569	ATP-dependent Clp protease ATP-binding subunitClpA
BPHY_RS02860	-2,255	1,26E-05	dut	Bphy_0570	dUTP diphosphatase
BPHY_RS02875	-1,521	0,004314		Bphy_0573	signal peptidase II
BPHY_RS02920	-1,183	0,004323	nadB	Bphy_0582	L-aspartate oxidase
BPHY_RS02930	-2,062	0,000466	rpmB	Bphy_0584	50S ribosomal protein L28
BPHY_RS02950	-1,965	2,4E-05		Bphy_0588	;C transportersubstrate-binding protein
BPHY_RS02955	-2,608	7,37E-07		Bphy_0589	branched-chain amino acid ABC transporterpermease
BPHY_RS02960	-2,586	2,1E-07		Bphy_0590	ABC transporter ATP-binding protein
BPHY_RS02965	-1,593	0,006258		Bphy_0591	ABC transporter ATP-binding protein
BPHY_RS02970	-1,419	0,003524		Bphy_0592	ABC transporter ATP-binding protein
BPHY_RS02975	-1,692	0,001577		Bphy_0593	GNAT family acetyltransferase

BPHY_RS02980	-1,255	0,006846		Bphy_0594	acetylornithine transaminase
BPHY_RS03010	-1,432	0,00055		Bphy_0601	amino acid permease
BPHY_RS03020	3,098	1,12E-10	hemN	Bphy_0602	oxygen-independent coproporphyrinogen IIIoxidase
BPHY_RS03040	-1,356	0,003087	trmB	Bphy_0606	tRNA (guanosine(46)-N7)-methyltransferase TrmB
BPHY_RS03055	1,122	0,003021		Bphy_0608	EthD family reductase
BPHY_RS03070	-2,810	5,71E-05		Bphy_0613	LysE family transporter
BPHY_RS03085	-1,228	0,001856		Bphy_0616	pyridoxal phosphate-dependent aminotransferase
BPHY_RS03130	-1,041	0,00605		Bphy_0625	bifunctional transcriptionalregulator/glucokinase
BPHY_RS03145	-1,802	1,2E-05		Bphy_0628	ABC transporter substrate-binding protein
BPHY_RS03150	-1,911	0,002464		Bphy_0629	sugar ABC transporter permease
BPHY_RS03155	-1,342	0,007903		Bphy_0630	carbohydrate ABC transporter permease
BPHY_RS03225	1,585	0,000751		Bphy_0644	PAS domain S-box protein
BPHY_RS03230	2,600	9,26E-07		Bphy_0645	response regulator
BPHY_RS03235	2,307	0,000966		Bphy_0646	response regulator
BPHY_RS03250	2,901	1,39E-05		Bphy_0649	efflux transporter outer membrane subunit
BPHY_RS03325	-2,534	1,96E-08		Bphy_0663	MHS family MFS transporter
BPHY_RS03375	-1,602	0,005178		Bphy_0672	DUF2591 family protein
BPHY_RS03435	-2,866	0,004805		Bphy_0682	LysR family transcriptional regulator
BPHY_RS03445	-4,259	2,22E-06		Bphy_0684	acyl-CoA dehydrogenase family protein
BPHY_RS03470	-2,097	0,003192		Bphy_0688	TMEM165/GDT1 family protein
BPHY_RS03480	-1,163	0,005078		Bphy_0690	1-acyl-sn-glycerol-3-phosphate acyltransferase
BPHY_RS03490	-6,499	0,000346		Bphy_0692	acyl carrier protein
BPHY_RS03500	-1,710	9,57E-05		Bphy_0694	AMP-binding protein
BPHY_RS03505	-1,668	1,91E-05		Bphy_0695	glycosyltransferase family 2 protein
BPHY_RS03510	-1,744	0,000112		Bphy_0696	aromatic amino acid ammonia-lyase
BPHY_RS03520	-1,657	0,00819		Bphy_0698	outer membrane lipoprotein carrier protein LolA
BPHY_RS03525	-1,224	0,001098		Bphy_0699	MMPL family transporter
BPHY_RS03530	-1,314	0,001668		Bphy_0700	beta-ketoacyl-[acyl-carrier-protein] synthasefamily protein
BPHY_RS03540	-1,654	0,009098		Bphy_0702	3-ketoacyl-ACP reductase FabG2
BPHY_RS03625	-2,071	0,002341		Bphy_0719	hypothetical protein
BPHY_RS03655	1,373	2,36E-05		Bphy_0725	FAD-dependent monooxygenase
BPHY_RS03660	1,781	0,008229		Bphy_0726	formate dehydrogenase subunit delta
BPHY_RS03725	-1,487	0,000167		Bphy_0739	DUF2059 domain-containing protein
BPHY_RS03735	-1,128	0,008125	pheA	Bphy_0741	prephenate dehydratase
BPHY_RS03740	-1,343	0,001063		Bphy_0742	histidinol-phosphate transaminase
BPHY_RS03745	-1,501	0,005482		Bphy_0743	prephenate dehydrogenase/arogenate dehydrogenasefamily protein
BPHY_RS03750	-1,288	0,004992	aroA	Bphy_0744	3-phosphoshikimate 1-carboxyvinyltransferase
BPHY_RS03755	-1,529	0,006883		Bphy_0745	(d)CMP kinase
BPHY_RS03760	-2,356	4,71E-07	rpsA	Bphy_0746	30S ribosomal protein S1
BPHY_RS03830	-1,686	0,000571		Bphy_0759	methionine ABC transporter ATP-binding protein
BPHY_RS03840	-2,247	3,01E-06		Bphy_0761	MetQ/NlpA family ABC transportersubstrate-binding protein
BPHY_RS03855	1,190	0,001506		Bphy_0764	acyl-CoA dehydrogenase
BPHY_RS03860	4,529	3,74E-39		Bphy_0765	D-amino acid dehydrogenase

BPHY_RS03865	2,895	1,05E-10		Bphy_0766	Lrp/AsnC ligand binding domain-containingprotein
BPHY_RS03880	-1,991	0,008868	rpsP	Bphy_0769	30S ribosomal protein S16
BPHY_RS03885	-1,757	0,002664	rimM	Bphy_0770	ribosome maturation factor RimM
BPHY_RS03890	-1,737	0,000208	trmD	Bphy_0771	tRNA (guanosine(37)-N1)-methyltransferase TrmD
BPHY_RS03895	-3,095	3,49E-10	rplS	Bphy_0772	50S ribosomal protein L19
BPHY_RS03945	-1,120	0,004426		Bphy_0782	dihydrofolate reductase
BPHY_RS03950	-4,352	0,000305		Bphy_0783	sigma-54 dependent transcriptional regulator
BPHY_RS03975	1,246	0,000102		Bphy_0787	sigma-54 dependent transcriptional regulator
BPHY_RS04010	1,327	0,000121	fumC	Bphy_0795	class II fumarate hydratase
BPHY_RS04135	-1,694	0,000694		Bphy_0818	Rne/Rng family ribonuclease
BPHY_RS04180	-1,758	0,000306	plsX	Bphy_0827	phosphate acyltransferase PlsX
BPHY_RS04185	-1,347	0,004827		Bphy_0828	ketoacyl-ACP synthase III
BPHY_RS04190	-1,285	0,004337	fabD	Bphy_0829	ACP S-malonyltransferase
BPHY_RS04195	-1,582	0,002073	fabG	Bphy_0830	3-oxoacyl-ACP reductase FabG
BPHY_RS04240	-1,331	0,002885	lepA	Bphy_0839	translation elongation factor 4
BPHY_RS04245	-1,653	0,001115	lepB	Bphy_0840	signal peptidase I
BPHY_RS04275	-1,401	0,000726	nagZ	Bphy_0846	beta-N-acetylhexosaminidase
BPHY_RS04290	-2,311	7,96E-05	efp	Bphy_0849	elongation factor P
BPHY_RS04420	-1,884	5,48E-06		Bphy_0872	MFS transporter
BPHY_RS04425	-1,325	0,002436	carA	Bphy_0873	ıoyl-phosphatesynthase small subunit
BPHY_RS04430	-1,655	0,000221	carB	Bphy_0874	carbamoyl-phosphate synthase large subunit
BPHY_RS04470	-2,011	0,000177	pstS	Bphy_0882	phosphate ABC transporter substrate-bindingprotein PstS
BPHY_RS04580	1,413	0,004901		Bphy_0904	hypothetical protein
BPHY_RS04615	-2,160	5,16E-05	rpsF	Bphy_0911	30S ribosomal protein S6
BPHY_RS04620	-1,981	0,000233	priB	Bphy_0912	primosomal replication protein N
BPHY_RS04625	-1,892	1,62E-05		Bphy_0913	30S ribosomal protein S18
BPHY_RS04630	-2,424	1,11E-07	rplI	Bphy_0914	50S ribosomal protein L9
BPHY_RS04640	-1,311	0,009379		Bphy_0916	DUF47 family protein
BPHY_RS04650	1,181	0,001219		Bphy_0918	SDR family oxidoreductase
BPHY_RS04655	1,402	0,001422		Bphy_0919	C40 family peptidase
BPHY_RS04660	1,844	2,3E-07		Bphy_0920	PhoH family protein
BPHY_RS04685	-1,319	0,005881		Bphy_0924	formyltransferase
BPHY_RS04700	-1,734	0,000788		Bphy_0927	4-amino-4-deoxy-L-arabinose transferase
BPHY_RS04705	-1,499	0,001654		Bphy_0928	glycosyltransferase family 39 protein
BPHY_RS04715	-1,376	0,002898		Bphy_0930	pyridoxal phosphate-dependent aminotransferase
BPHY_RS04720	-2,142	3,71E-06		Bphy_0931	homoserine dehydrogenase
BPHY_RS04725	-1,725	6,94E-05		Bphy_0932	threonine synthase
BPHY_RS04755	1,781	3,49E-07	clpB	Bphy_0938	ATP-dependent chaperone ClpB
BPHY_RS04765	3,081	1,88E-15		Bphy_0940	helix-hairpin-helix domain-containing protein
BPHY_RS04790	-1,672	6,87E-05		Bphy_0945	MATE family efflux transporter
BPHY_RS04800	-1,923	0,0005		Bphy_0947	type B 50S ribosomal protein L31
BPHY_RS04810	-1,256	0,007199	rho	Bphy_0949	transcription termination factor Rho
BPHY_RS04840	-1,314	0,00234		Bphy_0955	ABC transporter substrate-binding protein

BPHY_RS04855	1,100	0,007077		Bphy_0958	NADPH:quinone oxidoreductase family protein
BPHY_RS04875	1,018	0,00739	rpoS	Bphy_0962	RNA polymerase sigma factor RpoS
BPHY_RS04890	-1,300	0,005556	rlmD	Bphy_0965	23S rRNA (uracil(1939)-C(5))-methyltransferaseRlmD
BPHY_RS04915	1,911	1,83E-06		Bphy_0970	acetyl-CoA C-acetyltransferase
BPHY_RS04955	-1,176	0,005219	serB	Bphy_0978	phosphoserine phosphatase SerB
BPHY_RS04990	2,047	2,1E-08		Bphy_0985	DUF6013 family protein
BPHY_RS05010	1,718	6,01E-06		Bphy_0989	hypothetical protein
BPHY_RS05055	-1,396	0,001772		Bphy_0998	2-C-methyl-D-erythritol 4-phosphatecytidylyltransferase
BPHY_RS05070	1,745	6,45E-06		Bphy_1001	peroxiredoxin
BPHY_RS05120	-1,380	0,009366		Bphy_1010	trigger factor
BPHY_RS05135	1,653	3,56E-05	lon	Bphy_1013	endopeptidase La
BPHY_RS05155	-1,319	0,004101		Bphy_1015	SurA N-terminal domain-containing protein
BPHY_RS05190	-1,283	0,005219	purL	Bphy_1022	phosphoribosylformylglycinamidine synthase
BPHY_RS05195	-1,353	0,000938		Bphy_1023	peptidylprolyl isomerase
BPHY_RS05220	1,330	0,000343		Bphy_1028	enoyl-CoA hydratase/isomerase family protein
BPHY_RS05250	-1,939	0,005643		Bphy_1034	TRIC cation channel family protein
BPHY_RS05320	1,306	0,000104	otsA	Bphy_1048	alpha,alpha-trehalose-phosphate synthase(UDP-forming)
BPHY_RS05335	2,911	9,36E-16		Bphy_1051	hypothetical protein
BPHY_RS05350	2,164	6,42E-07		Bphy_1054	YciI family protein
BPHY_RS05405	1,324	0,009814		Bphy_1065	glycosyltransferase family 4 protein
BPHY_RS05410	1,743	0,009731		Bphy_1066	glycosyltransferase family 4 protein
BPHY_RS05420	-1,183	0,006725		Bphy_1068	HipA domain-containing protein
BPHY_RS05425	1,726	0,001249	gmd	Bphy_1069	GDP-mannose 4,6-dehydratase
BPHY_RS05460	1,247	0,007756	galU	Bphy_1077	UTP--glucose-1-phosphate uridylyltransferaseGalU
BPHY_RS05500	2,647	3,67E-12		Bphy_1085	hypothetical protein
BPHY_RS05505	2,454	0,003327		Bphy_1086	hypothetical protein
BPHY_RS05510	4,211	3,26E-09			hypothetical protein
BPHY_RS05515	2,481	0,001038		Bphy_1087	hypothetical protein
BPHY_RS05525	2,856	1,54E-11	arcD	Bphy_1089	arginine-ornithine antiporter
BPHY_RS05530	3,318	9,12E-12	arcA	Bphy_1090	arginine deiminase
BPHY_RS05535	3,705	6,27E-23		Bphy_1091	hypothetical protein
BPHY_RS05540	3,995	1,28E-28		Bphy_1092	PAS domain S-box protein
BPHY_RS05545	5,087	3,06E-25		Bphy_1093	hypothetical protein
BPHY_RS05550	5,372	1,28E-36		Bphy_1094	CBS domain-containing protein
BPHY_RS05555	3,553	6,51E-12		Bphy_1095	TIGR00730 family Rossman fold protein
BPHY_RS05565	1,775	9,74E-08	mgtA	Bphy_1097	magnesium-translocating P-type ATPase
BPHY_RS05570	3,736	1,32E-18		Bphy_1098	1-phosphofructokinase family hexose kinase
BPHY_RS05575	2,687	2,26E-10	ppsA	Bphy_1099	phosphoenolpyruvate synthase
BPHY_RS05580	4,357	2,88E-26		Bphy_1100	hypothetical protein
BPHY_RS05585	4,823	3,85E-41		Bphy_1101	BON domain-containing protein
BPHY_RS05590	2,845	7,87E-13		Bphy_1102	universal stress protein
BPHY_RS05595	3,200	5,79E-15		Bphy_1103	cation-translocating P-type ATPase
BPHY_RS05600	3,557	1,2E-12		Bphy_1104	carboxypeptidase regulatory-likedomain-containing protein

BPHY_RS05605	2,978	6,61E-09		Bphy_1105	MBL fold metallo-hydrolase
BPHY_RS05610	5,354	8,24E-39		Bphy_1106	alpha/beta fold hydrolase
BPHY_RS05615	5,227	8,8E-18		Bphy_1107	hypothetical protein
BPHY_RS05620	5,044	2,74E-34		Bphy_1108	BON domain-containing protein
BPHY_RS05625	4,641	1,18E-33		Bphy_1109	SagB/ThcOx family dehydrogenase
BPHY_RS05630	4,589	2,8E-24		Bphy_1110	Hsp20/alpha crystallin family protein
BPHY_RS05635	4,995	1,44E-39		Bphy_1111	BON domain-containing protein
BPHY_RS05640	2,917	1,77E-05	ftsH	Bphy_1112	ATP-dependent zinc metalloprotease FtsH
BPHY_RS05645	3,931	3,05E-16	groL	Bphy_1113	chaperonin GroEL
BPHY_RS05650	3,421	2,95E-25	phaZ	Bphy_1114	polyhydroxyalkanoate depolymerase
BPHY_RS05655	3,603	4,05E-19		Bphy_1115	osphotransferase/ATP-binding protein
BPHY_RS05660	5,090	2E-24		Bphy_1116	DUF1488 domain-containing protein
BPHY_RS05665	4,913	9,1E-60		Bphy_1117	universal stress protein
BPHY_RS05690	2,229	0,007285		Bphy_1122	F0F1 ATP synthase subunit alpha
BPHY_RS05695	3,279	7,73E-10		Bphy_1123	F0F1 ATP synthase subunit delta
BPHY_RS05700	2,893	0,000284		Bphy_1124	F0F1 ATP synthase subunit C
BPHY_RS05705	3,666	8,94E-10		Bphy_1125	F0F1 ATP synthase subunit A
BPHY_RS05715	3,061	3,12E-05		Bphy_1127	AtpZ/AtpI family protein
BPHY_RS05720	3,708	1,82E-06		Bphy_1128	F0F1 ATP synthase subunit epsilon
BPHY_RS05725	3,491	5,39E-10		Bphy_1129	F0F1 ATP synthase subunit beta
BPHY_RS05730	4,822	1,96E-27		Bphy_1130	LemA family protein
BPHY_RS05735	3,527	1,78E-11		Bphy_1131	beta-ketoacyl-ACP reductase
BPHY_RS05740	3,037	0,000501		Bphy_1132	hypothetical protein
BPHY_RS05745	3,893	9,35E-29		Bphy_1133	universal stress protein
BPHY_RS05750	4,778	5,12E-28		Bphy_1134	universal stress protein
BPHY_RS05755	4,972	1,02E-29		Bphy_1135	universal stress protein
BPHY_RS05775	4,186	4,13E-13		Bphy_1139	nitroreductase family protein
BPHY_RS05780	3,560	8,86E-06		Bphy_1140	c-type cytochrome
BPHY_RS05785	4,050	4,82E-23		Bphy_1141	DUF3564 domain-containing protein
BPHY_RS05790	4,483	5,99E-26		Bphy_1142	hypothetical protein
BPHY_RS05795	3,101	1,36E-25		Bphy_1143	PAS domain S-box protein
BPHY_RS05800	3,194	1,22E-17		Bphy_1144	response regulator
BPHY_RS05805	2,783	3,52E-13		Bphy_1145	PAS domain-containing protein
BPHY_RS05810	3,350	4,86E-07		Bphy_1146	CreA family protein
BPHY_RS05815	3,743	6,11E-07		Bphy_1147	hypothetical protein
BPHY_RS05845	-2,500	0,008354		Bphy_1153	C45 family autoproteolyticacyltransferase/hydolase
BPHY_RS05850	-3,499	0,001213	glnQ	Bphy_1154	glutamine ABC transporter ATP-binding proteinGlnQ
BPHY_RS05895	1,445	0,000113	ppk2	Bphy_1162	polyphosphate kinase 2
BPHY_RS05900	3,805	1,74E-11		Bphy_1163	YdcH family protein
BPHY_RS06040	1,285	0,004929		Bphy_1190	response regulator
BPHY_RS06120	1,643	0,009389		Bphy_1203	hypothetical protein
BPHY_RS06125	1,983	3,27E-08		Bphy_1204	oxidoreductase
BPHY_RS06140	1,904	1,43E-05		Bphy_1208	hypothetical protein

BPHY_RS06145	1,199	0,004851		Bphy_1209	DnaJ domain-containing protein
BPHY_RS06235	3,421	1,96E-23		Bphy_1227	SpoVR family protein
BPHY_RS06240	3,334	2,86E-20		Bphy_1228	YeaH/YhbH family protein
BPHY_RS06245	3,631	1,17E-21		Bphy_1229	PrkA family serine protein kinase
BPHY_RS06255	1,496	3,33E-05		Bphy_1231	hydratase
BPHY_RS06260	-2,586	2,52E-05		Bphy_1232	methyl-accepting chemotaxis protein
BPHY_RS06325	1,844	2,99E-05		Bphy_1246	Spy/CpxP family protein refolding chaperone
BPHY_RS06350	1,002	0,000706		Bphy_1251	D-glycerate dehydrogenase
BPHY_RS06380	2,348	1,08E-10		Bphy_1257	glutathione S-transferase
BPHY_RS06395	2,282	0,00401		Bphy_1260	hypothetical protein
BPHY_RS06400	1,821	0,001284		Bphy_1261	DUF2795 domain-containing protein
BPHY_RS06405	1,495	0,000129		Bphy_1262	AraC family transcriptional regulator
BPHY_RS06440	1,979	1,34E-07		Bphy_1269	hypothetical protein
BPHY_RS06470	-1,935	0,001479		Bphy_1275	methyl-accepting chemotaxis protein
BPHY_RS06480	-1,850	0,000468		Bphy_1277	ABC transporter permease subunit
BPHY_RS06485	-1,310	0,006826	potA	Bphy_1278	polyamine ABC transporter ATP-binding protein
BPHY_RS06505	3,893	1,22E-29		Bphy_1282	outer membrane beta-barrel protein
BPHY_RS06510	4,952	1,94E-46		Bphy_1283	nitronate monooxygenase family protein
BPHY_RS06515	1,232	0,000761		Bphy_1284	aldehyde dehydrogenase family protein
BPHY_RS06520	-1,286	0,008853		Bphy_1285	ABC-F family ATPase
BPHY_RS06540	1,209	0,008786		Bphy_1289	ABC transporter permease
BPHY_RS06570	1,799	0,000675	uraH	Bphy_1294	hydroxyisourate hydrolase
BPHY_RS06590	1,971	3,74E-07	alc	Bphy_1298	allantoicase
BPHY_RS06595	1,658	1,79E-06	uraD	Bphy_1299	2-oxo-4-hydroxy-4-carboxy-5-ureidoimidazolinedecarboxylase
BPHY_RS06600	1,522	7,29E-05	puuE	Bphy_1300	allantoinase PuuE
BPHY_RS06730	-1,389	0,0006	lpxA	Bphy_1326	acyl-ACP--UDP-N-acetylglucosamineO-acyltransferase
BPHY_RS06735	-1,353	0,00295	fabZ	Bphy_1327	3-hydroxyacyl-ACP dehydratase FabZ
BPHY_RS06780	-2,024	4,52E-05		Bphy_1336	UMP kinase
BPHY_RS06785	-1,982	0,000661		Bphy_1337	translation elongation factor Ts
BPHY_RS06790	-1,825	0,000259	rpsB	Bphy_1338	30S ribosomal protein S2
BPHY_RS06845	-1,511	0,005405	dapD	Bphy_1349	2,3,4,5-tetrahydropyridine-2,6-dicarboxylateN-succinyltransferase
BPHY_RS06850	-1,777	0,008401		Bphy_1350	ArsC family reductase
BPHY_RS06855	-1,235	0,007588	dapE	Bphy_1351	succinyl-diaminopimelate desuccinylase
BPHY_RS06870	1,390	0,000214		Bphy_1354	glutathione peroxidase
BPHY_RS06935	-2,246	1,52E-06		Bphy_1367	DEAD/DEAH box helicase
BPHY_RS06940	2,177	1,99E-09	aceA	Bphy_1368	isocitrate lyase
BPHY_RS07060	-2,312	2,23E-06		Bphy_1386	aspartate kinase
BPHY_RS07155	2,316	1,09E-12		Bphy_1404	DUF465 domain-containing protein
BPHY_RS07160	1,880	1,4E-07		Bphy_1405	hypothetical protein
BPHY_RS07165	-1,632	0,000205		Bphy_1406	RNA-binding transcriptional accessory protein
BPHY_RS07175	-1,259	0,00605		Bphy_1408	phosphoribosyltransferase
BPHY_RS07185	-1,271	0,002146		Bphy_1410	ATP phosphoribosyltransferase regulatorysubunit
BPHY_RS07225	-1,044	0,004171		Bphy_1418	tetratricopeptide repeat protein

BPHY_RS07230	-1,897	2,01E-05	hisS	Bphy_1419	histidine--tRNA ligase
BPHY_RS07235	-1,755	8,11E-05	ispG	Bphy_1420	[illegible]-3-methylbut-2-enyl-diphosphate synthase
BPHY_RS07250	-1,595	0,001176	ndk	Bphy_1423	nucleoside-diphosphate kinase
BPHY_RS07255	1,326	0,002732		Bphy_1424	Bax inhibitor-1/YccA family protein
BPHY_RS07265	-1,066	0,007041	bamC	Bphy_1426	outer membrane protein assembly factor BamC
BPHY_RS07320	1,618	0,000736		Bphy_1437	hypothetical protein
BPHY_RS07385	-1,260	0,0088	prfB	Bphy_1449	peptide chain release factor 2
BPHY_RS07390	-1,485	0,00286	lysS	Bphy_1450	lysine--tRNA ligase
BPHY_RS07395	1,313	0,005484		Bphy_1451	glycine zipper 2TM domain-containing protein
BPHY_RS07400	1,420	0,003081	iscX	Bphy_1452	Fe-S cluster assembly protein IscX
BPHY_RS07405	1,320	0,000752	fdx	Bphy_1453	ISC system 2Fe-2S type ferredoxin
BPHY_RS07410	-1,624	0,000293	hscA	Bphy_1454	Fe-S protein assembly chaperone HscA
BPHY_RS07435	1,391	2,55E-05	iscR	Bphy_1459	Fe-S cluster assembly transcriptional regulatorIscR
BPHY_RS07455	1,297	0,000101		Bphy_1463	lactate utilization protein
BPHY_RS07485	1,841	9,48E-06	aceF	Bphy_1469	dihydrolipoyllysine-residue acetyltransferase
BPHY_RS07490	1,381	6,19E-05	aceE	Bphy_1470	pyruvate dehydrogenase (acetyl-transferring),homodimeric type
BPHY_RS07505	2,293	5,53E-10		Bphy_1473	DUF3300 domain-containing protein
BPHY_RS07510	2,183	6,09E-08		Bphy_1474	DUF2950 domain-containing protein
BPHY_RS07525	1,424	0,000339		Bphy_1476	M3 family metallopeptidase
BPHY_RS07560	1,168	0,000899		Bphy_1482	rhodanese-like domain-containing protein
BPHY_RS07580	2,130	7,52E-11		Bphy_1486	beta-propeller fold lactonase family protein
BPHY_RS07605	-1,664	0,000139	proP	Bphy_1491	glycine betaine/L-proline transporter ProP
BPHY_RS07610	-1,868	0,000154		Bphy_1492	metallophosphatase family protein
BPHY_RS07615	-1,966	4E-07		Bphy_1493	hypothetical protein
BPHY_RS07630	1,437	0,000234		Bphy_1496	FAD-containing oxidoreductase
BPHY_RS07640	1,705	1,92E-05		Bphy_1498	glutathione S-transferase
BPHY_RS07715	1,326	0,001903		Bphy_1512	sensor histidine kinase
BPHY_RS07725	-3,387	0,004664		Bphy_1514	DUF4397 domain-containing protein
BPHY_RS07755	1,321	0,008823		Bphy_1521	L-lactate permease
BPHY_RS07785	1,493	0,000686		Bphy_1527	hypothetical protein
BPHY_RS07810	1,391	0,000525	hydA	Bphy_1532	dihydropyrimidinase
BPHY_RS07815	1,618	0,000756		Bphy_1533	NCS1 family nucleobase:cation symporter-1
BPHY_RS07825	2,213	2,88E-06		Bphy_1535	NAD(P)-dependent oxidoreductase
BPHY_RS07830	3,688	7,62E-19		Bphy_1536	Zn-dependent hydrolase
BPHY_RS07925	-1,458	0,000563	pheS	Bphy_1554	phenylalanine--tRNA ligase subunit alpha
BPHY_RS07930	-1,747	0,000394	rplT	Bphy_1555	50S ribosomal protein L20
BPHY_RS07935	-2,144	6,89E-05	rpmI	Bphy_1556	50S ribosomal protein L35
BPHY_RS07965	1,314	0,00032		Bphy_1561	alpha/beta hydrolase
BPHY_RS07970	1,435	0,000444		Bphy_1562	CoA transferase subunit B
BPHY_RS07975	1,341	0,003049		Bphy_1563	CoA transferase subunit A
BPHY_RS07980	-4,595	0,000258		Bphy_1564	PAS and helix-turn-helix domain-containingprotein
BPHY_RS07990	1,421	0,001303		Bphy_1566	SDR family oxidoreductase
BPHY_RS08020	1,572	3,83E-07		Bphy_1572	O-acetylhomoserineaminocarboxypropyltransferase

BPHY_RS08025	1,134	0,006142		Bphy_1573	alpha/beta hydrolase
BPHY_RS08055	1,093	0,005037		Bphy_1579	metallophosphoesterase
BPHY_RS08090	1,631	2,13E-05		Bphy_1587	MFS transporter
BPHY_RS08095	1,912	2,86E-08		Bphy_1588	propionate--CoA ligase
BPHY_RS08210	-2,038	0,0088		Bphy_1611	YbfB/YjiJ family MFS transporter
BPHY_RS08285	-1,296	0,00607		Bphy_1626	sulfate ABC transporter ATP-binding protein
BPHY_RS08300	-1,443	0,00084		Bphy_1629	sulfate ABC transporter substrate-bindingprotein
BPHY_RS08320	2,067	0,00286		Bphy_1633	universal stress protein
BPHY_RS08325	1,305	0,000655	nodI	Bphy_1634	nodulation factor ABC transporter ATP-bindingprotein NodI
BPHY_RS08340	-2,033	0,00051		Bphy_1637	permease
BPHY_RS08370	1,602	0,000211		Bphy_1642	hypothetical protein
BPHY_RS08430	1,603	0,001624		Bphy_1655	DUF1488 domain-containing protein
BPHY_RS08475	1,822	8,3E-08		Bphy_1663	arginyltransferase
BPHY_RS08480	-1,714	0,000723		Bphy_1664	quinone-dependent dihydroorotate dehydrogenase
BPHY_RS08500	1,853	2,39E-05		Bphy_1669	Crp/Fnr family transcriptional regulator
BPHY_RS08580	-2,183	0,00876		Bphy_1685	glycosyltransferase family 2 protein
BPHY_RS08600	-2,409	0,000125		Bphy_1689	AAA family ATPase
BPHY_RS08610	-5,753	0,001056		Bphy_1691	sugar transferase
BPHY_RS08670	1,681	0,000311		Bphy_1701	3-oxoacyl-ACP reductase
BPHY_RS08675	1,889	2,26E-06		Bphy_1702	acyl dehydratase
BPHY_RS08680	1,946	4,86E-06		Bphy_1703	acetyl-CoA C-acetyltransferase
BPHY_RS08770	-1,860	0,000626	typA	Bphy_1722	translational GTPase TypA
BPHY_RS08775	-1,690	0,000235		Bphy_1723	MarR family winged helix-turn-helixtranscriptional regulator
BPHY_RS08780	-1,841	2,83E-05		Bphy_1724	efflux transporter outer membrane subunit
BPHY_RS08785	-1,969	1,03E-06		Bphy_1725	ux transporterperiplasmic adaptor subunit
BPHY_RS08790	-2,165	4,36E-07		Bphy_1726	DHA2 family efflux MFS transporter permeasesubunit
BPHY_RS08795	-1,940	0,001548	truB	Bphy_1727	tRNA pseudouridine(55) synthase TruB
BPHY_RS08800	-1,964	0,000227	rbfA	Bphy_1728	30S ribosome-binding factor RbfA
BPHY_RS08805	-1,466	0,00217	infB	Bphy_1729	translation initiation factor IF-2
BPHY_RS08860	2,610	2,53E-07		Bphy_1741	hypothetical protein
BPHY_RS08880	1,374	2,98E-05	arfB	Bphy_1745	aminoacyl-tRNA hydrolase
BPHY_RS08920	2,337	5,26E-08		Bphy_1753	cysteine dioxygenase family protein
BPHY_RS08940	1,365	0,000407		Bphy_1757	hypothetical protein
BPHY_RS08945	3,856	2,51E-27		Bphy_1758	DJ-1/PfpI family protein
BPHY_RS08985	2,315	4,98E-07		Bphy_1766	CHAD domain-containing protein
BPHY_RS09010	-1,240	0,002695		Bphy_1771	RNDtransporter periplasmic adaptor subunit
BPHY_RS09015	-1,267	0,001736		Bphy_1772	flux RNDtransporter permease subunit
BPHY_RS09030	1,755	4,46E-06		Bphy_1775	peroxiredoxin
BPHY_RS09055	-3,151	1,22E-11		Bphy_1780	ABC transporter substrate-binding protein
BPHY_RS09090	1,301	0,000156		Bphy_1787	aspartate aminotransferase family protein
BPHY_RS09100	1,239	0,000422		Bphy_1789	ABC transporter ATP-binding protein
BPHY_RS09145	1,692	3,04E-05	glgC	Bphy_1797	glucose-1-phosphate adenylyltransferase
BPHY_RS09160	-3,198	0,000211		Bphy_1800	hypothetical protein

BPHY_RS09195	1,875	2,38E-06		Bphy_1807	hypothetical protein
BPHY_RS09200	1,370	2,76E-05	hutG	Bphy_1808	N-formylglutamate deformylase
BPHY_RS09215	1,733	5,25E-06		Bphy_1811	HutD family protein
BPHY_RS09220	1,977	3,94E-07		Bphy_1812	urocanate hydratase
BPHY_RS09225	1,737	0,000192	hutC	Bphy_1813	histidine utilization repressor
BPHY_RS09230	1,613	4,85E-05	hutH	Bphy_1814	histidine ammonia-lyase
BPHY_RS09245	2,164	6,07E-06		Bphy_1818	DUF899 domain-containing protein
BPHY_RS09290	1,114	0,007642		Bphy_1827	LysR family transcriptional regulator
BPHY_RS09295	6,445	5,77E-67		Bphy_1828	NAD(P)/FAD-dependent oxidoreductase
BPHY_RS09300	5,396	1,44E-22		Bphy_1829	LysR family transcriptional regulator ArgP
BPHY_RS09325	-4,357	0,001156		Bphy_1834	hypothetical protein
BPHY_RS09330	-3,937	0,005102		Bphy_1835	GAF domain-containing protein
BPHY_RS09340	-2,833	4,34E-05		Bphy_1837	tetratricopeptide repeat protein
BPHY_RS09345	-1,927	0,004912		Bphy_1839	sugar ABC transporter
BPHY_RS09365	1,710	0,009744		Bphy_1843	hypothetical protein
BPHY_RS09455	1,271	0,001316			hypothetical protein
BPHY_RS09545	-3,806	0,000556		Bphy_1874	DNA circularization N-terminal domain-containingprotein
BPHY_RS09595	-2,643	0,005399		Bphy_1885	S49 family peptidase
BPHY_RS09600	-7,923	1,62E-06		Bphy_1886	GIY-YIG nuclease family protein
BPHY_RS09605	-2,538	0,004006		Bphy_1887	phage portal protein
BPHY_RS09625	-6,746	0,001791		Bphy_1891	phage tail protein
BPHY_RS09685	-7,105	1,64E-05		Bphy_1903	DUF1064 domain-containing protein
BPHY_RS09765	-2,014	0,006747		Bphy_1917	DNA cytosine methyltransferase
BPHY_RS09795	1,112	0,00403		Bphy_1924	phage Gp37/Gp68 family protein
BPHY_RS09865	-1,812	0,008005		Bphy_1939	DMT family transporter
BPHY_RS09870	-1,198	0,007075		Bphy_1940	TIGR00645 family protein
BPHY_RS09875	-1,705	8,27E-05		Bphy_1941	fumarate hydratase
BPHY_RS09880	1,440	0,000309	bfr	Bphy_1942	bacterioferritin
BPHY_RS09895	-4,245	6,08E-20		Bphy_1945	energy transducer TonB
BPHY_RS09900	-4,040	5,51E-16		Bphy_1946	MotA/TolQ/ExbB proton channel family protein
BPHY_RS09905	-3,140	1,21E-07		Bphy_1947	biopolymer transporter ExbD
BPHY_RS09930	-2,327	0,000428		Bphy_1952	hemin uptake protein HemP
BPHY_RS09940	-1,936	0,000112		Bphy_1954	4Fe-4S binding protein
BPHY_RS09945	-2,286	1,17E-05		Bphy_1955	FTR1 family protein
BPHY_RS09950	-2,541	1,05E-05		Bphy_1956	cupredoxin domain-containing protein
BPHY_RS09955	-2,579	1,58E-06		Bphy_1957	iron transporter
BPHY_RS09970	-1,808	8,31E-05		Bphy_1959	aspartate/tyrosine/aromatic aminotransferase
BPHY_RS09975	1,095	0,00237		Bphy_1960	patatin-like phospholipase family protein
BPHY_RS09980	1,510	0,000179		Bphy_1961	3-hydroxybutyrate dehydrogenase
BPHY_RS09985	1,707	3,1E-05		Bphy_1962	aldo/keto reductase
BPHY_RS10005	1,452	0,000109		Bphy_1965	hypothetical protein
BPHY_RS10065	1,681	1,06E-05		Bphy_1973	AI-2E family transporter
BPHY_RS10080	1,703	3,5E-05		Bphy_1976	energy-coupling factor ABC transporter permease

BPHY_RS10135	1,910	9,08E-07		Bphy_1987	acyl-CoA dehydrogenase family protein
BPHY_RS10140	1,974	3,01E-06		Bphy_1988	glutathione S-transferase N-terminaldomain-containing protein
BPHY_RS10150	1,380	0,003722		Bphy_1990	MaoC family dehydratase
BPHY_RS10195	-1,699	0,002584	nuoK	Bphy_1999	NADH-quinone oxidoreductase subunit NuoK
BPHY_RS10225	-1,216	0,004795	nuoE	Bphy_2005	NADH-quinone oxidoreductase subunit NuoE
BPHY_RS10270	-1,909	4,58E-05	pnp	Bphy_2013	polyribonucleotide nucleotidyltransferase
BPHY_RS10275	-2,195	1,3E-06	rpsO	Bphy_2014	30S ribosomal protein S15
BPHY_RS10300	-1,345	0,008184	pssA	Bphy_2018	CDP-diacylglycerol--serineO-phosphatidyltransferase
BPHY_RS10310	-1,698	0,000118	ilvC	Bphy_2020	ketol-acid reductoisomerase
BPHY_RS10315	-2,303	3,39E-06	ilvN	Bphy_2021	acetolactate synthase small subunit
BPHY_RS10320	-2,014	3,35E-05		Bphy_2022	acetolactate synthase 3 catalytic subunit
BPHY_RS10375	-1,381	0,009994		Bphy_2033	glycosyl transferase
BPHY_RS10405	-2,290	0,001755		Bphy_2040	copper oxidase
BPHY_RS10410	-2,243	0,001584		Bphy_2041	TolC family protein
BPHY_RS10425	1,592	9,01E-06		Bphy_2044	permease
BPHY_RS10430	1,263	0,004104		Bphy_2045	DUF305 domain-containing protein
BPHY_RS10435	3,066	4,32E-14		Bphy_2046	LemA family protein
BPHY_RS10440	3,488	2,59E-09		Bphy_2047	YgcG family protein
BPHY_RS10445	2,519	1,48E-10		Bphy_2048	TPM domain-containing protein
BPHY_RS10460	1,355	2,67E-05		Bphy_2051	DUF4126 domain-containing protein
BPHY_RS10485	-2,638	0,000597	kdpB	Bphy_2056	potassium-transporting ATPase subunit KdpB
BPHY_RS10490	-3,579	0,003558	kdpA	Bphy_2057	potassium-transporting ATPase subunit KdpA
BPHY_RS10505	1,156	0,002973		Bphy_2060	quinone oxidoreductase
BPHY_RS10530	-1,684	0,000737	purD	Bphy_2065	phosphoribosylamine--glycine ligase
BPHY_RS10535	-1,228	0,009971	hemF	Bphy_2066	oxygen-dependent coproporphyrinogen oxidase
BPHY_RS10540	-1,188	0,007491		Bphy_2067	nicotinate-nucleotide adenylyltransferase
BPHY_RS10550	-1,502	0,000532	rlmH	Bphy_2069	23S rRNA(pseudouridine(1915)-N(3))-methyltransferase RlmH
BPHY_RS10570	1,768	0,002473		Bphy_2074	hypothetical protein
BPHY_RS10615	1,680	7,74E-05		Bphy_2083	aldehyde dehydrogenase family protein
BPHY_RS10620	1,517	5,31E-06		Bphy_2084	acetolactate synthase large subunit
BPHY_RS10640	-1,275	0,003209		Bphy_2088	META domain-containing protein
BPHY_RS10645	-1,108	0,002609		Bphy_2089	hypothetical protein
BPHY_RS10650	1,516	5,5E-05		Bphy_2090	lactonase family protein
BPHY_RS10670	1,238	0,002376		Bphy_2095	TetR family transcriptional regulator
BPHY_RS10710	-1,240	0,005502		Bphy_2103	benzoate/H(+) symporter BenE family transporter
BPHY_RS10720	1,347	3,43E-05		Bphy_2105	VOC family protein
BPHY_RS10730	-2,085	7,47E-07		Bphy_2107	sodium:solute symporter
BPHY_RS10735	-6,212	0,001364		Bphy_2108	DUF3311 domain-containing protein
BPHY_RS10740	-2,204	1,05E-06		Bphy_2109	spermidine synthase
BPHY_RS10745	-1,721	0,000804		Bphy_2110	DNA-deoxyinosine glycosylase
BPHY_RS10760	1,570	0,000108	htpG	Bphy_2113	molecular chaperone HtpG
BPHY_RS10820	1,490	0,000429		Bphy_2126	DUF4399 domain-containing protein
BPHY_RS10905	2,508	2,37E-19		Bphy_2142	YbhB/YbcL family Raf kinase inhibitor-likeprotein

BPHY_RS10920	1,432	0,001493	otsA	Bphy_2145	alpha,alpha-trehalose-phosphate synthase(UDP-forming)
BPHY_RS10940	1,984	0,002234		Bphy_2149	hypothetical protein
BPHY_RS10945	2,321	0,000167		Bphy_2150	DUF883 family protein
BPHY_RS11010	-2,336	6,52E-05		Bphy_2163	ABC transporter substrate-binding protein
BPHY_RS11015	-2,316	1,24E-05	hisQ	Bphy_2164	histidine ABC transporter permease HisQ
BPHY_RS11020	-2,389	4,05E-06		Bphy_2165	ABC transporter permease
BPHY_RS11025	-2,767	3,67E-08		Bphy_2166	ATP-binding cassette domain-containing protein
BPHY_RS11070	2,304	3,61E-12		Bphy_2174	aldehyde dehydrogenase family protein
BPHY_RS11075	1,794	0,000371		Bphy_2175	SDR family oxidoreductase
BPHY_RS11105	-1,053	0,009608		Bphy_2181	class I SAM-dependent methyltransferase
BPHY_RS11120	1,183	0,00113		Bphy_2184	hypothetical protein
BPHY_RS11160	-2,464	6,99E-06		Bphy_2192	adhesin
BPHY_RS11185	-1,348	0,00973		Bphy_2197	pantoate--beta-alanine ligase
BPHY_RS11190	-1,539	0,003859		Bphy_2198	aspartate 1-decarboxylase
BPHY_RS11205	-1,736	1,21E-05		Bphy_2201	tetratricopeptide repeat protein
BPHY_RS11215	-1,006	0,008408		Bphy_2203	cobyric acid synthase
BPHY_RS11330	-1,841	4,39E-05	lptG	Bphy_2227	LPS export ABC transporter permease LptG
BPHY_RS11345	-2,097	8,86E-06	cobA	Bphy_2230	uroporphyrinogen-III C-methyltransferase
BPHY_RS11350	-1,592	4,15E-05		Bphy_2231	sulfate adenylyltransferase subunit 1
BPHY_RS11355	-1,456	0,000275	cysD	Bphy_2232	sulfate adenylyltransferase subunit CysD
BPHY_RS11360	-1,841	2,1E-05		Bphy_2233	phosphoadenylyl-sulfate reductase
BPHY_RS11365	-1,963	5,96E-05		Bphy_2234	DUF934 domain-containing protein
BPHY_RS11370	-1,625	3,61E-05		Bphy_2235	nitrite/sulfite reductase
BPHY_RS11385	-1,676	4,78E-05		Bphy_2238	hypothetical protein
BPHY_RS11440	-4,578	1,46E-06		Bphy_2249	DUF3443 family protein
BPHY_RS11470	1,035	0,003966	urtE	Bphy_2255	urea ABC transporter ATP-binding subunit UrtE
BPHY_RS11535	-1,425	0,001513		Bphy_2268	oligosaccharide flippase family protein
BPHY_RS11540	-1,763	0,000274		Bphy_2269	glycosyltransferase
BPHY_RS11625	-1,969	0,001993		Bphy_2287	methyl-accepting chemotaxis protein
BPHY_RS11645	-1,924	0,001719		Bphy_2291	SDR family oxidoreductase
BPHY_RS11665	-1,929	5,74E-05		Bphy_2295	glycosyltransferase family 2 protein
BPHY_RS11670	-1,585	0,00048		Bphy_2296	glycosyltransferase family 2 protein
BPHY_RS11675	-1,335	0,004178		Bphy_2297	NAD-dependent epimerase/dehydratase familyprotein
BPHY_RS11685	-1,821	0,000423		Bphy_2299	glycosyltransferase
BPHY_RS11690	-1,534	0,001371		Bphy_2300	acyltransferase
BPHY_RS11695	-1,542	0,002769		Bphy_2301	glycosyltransferase family 39 protein
BPHY_RS11760	-2,137	7,67E-05		Bphy_2314	ABC transporter permease
BPHY_RS11765	-1,209	0,001543		Bphy_2315	glycosyltransferase family 2 protein
BPHY_RS11775	-1,640	0,001792	rfbC	Bphy_2317	dTDP-4-dehydrorhamnose 3,5-epimerase
BPHY_RS11785	-1,320	0,006416	rfbB	Bphy_2319	dTDP-glucose 4,6-dehydratase
BPHY_RS11830	1,079	0,001493		Bphy_2328	hypothetical protein
BPHY_RS11850	1,397	0,003232	groL	Bphy_2333	chaperonin GroEL
BPHY_RS11855	1,608	0,005756	groES	Bphy_2334	co-chaperone GroES

BPHY_RS11865	-5,861	0,005923			hypothetical protein
BPHY_RS11870	-4,121	0,000405		Bphy_2336	hypothetical protein
BPHY_RS11875	-4,059	0,000146		Bphy_2337	MFS transporter
BPHY_RS11890	-3,140	0,009649	xylG	Bphy_2340	D-xylose ABC transporter ATP-binding protein
BPHY_RS11895	-3,207	0,001943	xylF	Bphy_2341	D-xylose ABC transporter substrate-bindingprotein
BPHY_RS11900	-1,905	0,006902	xylA	Bphy_2342	xylose isomerase
BPHY_RS11915	-2,635	0,00026		Bphy_2345	PepSY-associated TM helix domain-containingprotein
BPHY_RS11920	-1,678	0,003615		Bphy_2346	SDR family oxidoreductase
BPHY_RS11945	-3,536	0,001277		Bphy_2351	extracellular solute-binding protein
BPHY_RS11965	1,087	0,003269		Bphy_2355	MerR family transcriptional regulator
BPHY_RS11980	1,434	0,000223		Bphy_2358	aldo/keto reductase
BPHY_RS11995	3,586	3,64E-17		Bphy_2360	pyridoxamine 5'-phosphate oxidase familyprotein
BPHY_RS12030	-4,170	0,000596		Bphy_2367	amino acid ABC transporter ATP-binding protein
BPHY_RS12035	-7,366	6,22E-06		Bphy_2368	amino acid ABC transporter permease
BPHY_RS12105	-2,474	0,004393		Bphy_2382	polyamine ABC transporter substrate-bindingprotein
BPHY_RS12125	-2,421	0,007247		Bphy_2386	tyrosine-type recombinase/integrase
BPHY_RS12165	3,037	1,66E-25		Bphy_2395	hypothetical protein
BPHY_RS12170	3,193	7,59E-16		Bphy_2396	DUF2252 domain-containing protein
BPHY_RS12175	1,622	7,12E-07		Bphy_2397	SulP family inorganic anion transporter
BPHY_RS12180	1,262	0,008655		Bphy_2399	ROK family protein
BPHY_RS12185	1,874	3,62E-07		Bphy_2400	glucose-6-phosphate dehydrogenase
BPHY_RS12190	1,762	7,45E-10		Bphy_2401	glucosidase
BPHY_RS12220	1,500	0,00176		Bphy_2408	bacterioferritin
BPHY_RS12315	3,653	9,69E-37		Bphy_2428	LysR family transcriptional regulator
BPHY_RS12320	2,829	6,69E-14		Bphy_2429	2-hydroxyacid dehydrogenase
BPHY_RS12335	-3,804	7,69E-05		Bphy_2432	MFS transporter
BPHY_RS12400	-8,103	2,71E-06		Bphy_2445	PilW family protein
BPHY_RS12415	-1,735	1,74E-05	nrdR	Bphy_2448	transcriptional regulator NrdR
BPHY_RS12425	-1,153	0,009188	ydfG	Bphy_2450	[illegible] dehydrogenase/3-hydroxypropionate dehydrogenase YdfG
BPHY_RS12450	-1,494	0,000965	tolB	Bphy_2455	Tol-Pal system beta propeller repeat proteinTolB
BPHY_RS12460	-1,653	0,000637	ybgF	Bphy_2457	tol-pal system protein YbgF
BPHY_RS12475	-2,288	0,003927		Bphy_2458	glycosyltransferase
BPHY_RS12480	-3,112	0,000983		Bphy_2459	oligosaccharide flippase family protein
BPHY_RS12490	-4,110	0,000101		Bphy_2461	acyltransferase
BPHY_RS12505	-3,654	0,00237		Bphy_2464	glycosyltransferase
BPHY_RS12525	-7,496	2,03E-05		Bphy_2468	glycosyltransferase WbuB
BPHY_RS12555	-3,134	0,00448		Bphy_2474	undecaprenyl-phosphate glucosephosphotransferase
BPHY_RS12590	-4,046	9,44E-11		Bphy_2481	MFS transporter
BPHY_RS12595	-7,050	3,9E-05		Bphy_2482	hypothetical protein
BPHY_RS12705	1,357	0,000147	hrcA	Bphy_2503	heat-inducible transcriptional repressor HrcA
BPHY_RS12825	-1,657	0,001059	yajC	Bphy_2526	preprotein translocase subunit YajC
BPHY_RS12830	-1,807	0,000113	secD	Bphy_2527	protein translocase subunit SecD

BPHY_RS12835	-1,589	0,000981	secF	Bphy_2528	protein translocase subunit SecF
BPHY_RS12840	-1,484	0,008109		Bphy_2529	MFS transporter
BPHY_RS12895	1,666	2,56E-06		Bphy_2541	NAD(P)(+) transhydrogenase (Re/Si-specific)subunit beta
BPHY_RS12905	1,750	7,67E-08		Bphy_2543	Re/Si-specific NAD(P)(+) transhydrogenasesubunit alpha
BPHY_RS12955	-1,292	0,000671	purH	Bphy_2553	arboxamideformyltransferase/IMP cyclohydrolase
BPHY_RS12965	-1,253	0,003298		Bphy_2555	LysE family translocator
BPHY_RS12980	-1,195	0,008348		Bphy_2558	histidine phosphatase family protein
BPHY_RS13005	-2,268	8,02E-05	rpsI	Bphy_2563	30S ribosomal protein S9
BPHY_RS13010	-2,104	1,87E-05	rplM	Bphy_2564	50S ribosomal protein L13
BPHY_RS13045	-1,730	0,00039	gltK	Bphy_2571	glutamate/aspartate ABC transporter permeaseGltK
BPHY_RS13050	-1,714	0,000342		Bphy_2572	amino acid ABC transporter permease
BPHY_RS13065	-1,074	0,009408		Bphy_2575	LysR family transcriptional regulator
BPHY_RS13085	1,955	2,34E-07	eda	Bphy_2579	ase/2-dehydro-3-deoxy-phosphogluconate aldolase
BPHY_RS13095	-2,455	0,002658		Bphy_2581	MurR/RpiR family transcriptional regulator
BPHY_RS13105	-2,422	0,000565		Bphy_2583	YadA-like family protein
BPHY_RS13120	-1,735	4,89E-05		Bphy_2586	DNA polymerase III subunit delta
BPHY_RS13165	-2,235	0,003788	alc	Bphy_2595	allantoicase
BPHY_RS13225	-1,440	0,002504		Bphy_2607	CinA family protein
BPHY_RS13235	-1,304	0,004096	pyrF	Bphy_2609	orotidine-5'-phosphate decarboxylase
BPHY_RS13245	-3,128	6,11E-05		Bphy_2611	SMP-30/gluconolactonase/LRE family protein
BPHY_RS13255	-2,914	2,55E-06	araH	Bphy_2613	L-arabinose ABC transporter permease AraH
BPHY_RS13260	-1,728	0,00091	araG	Bphy_2614	L-arabinose ABC transporter ATP-binding proteinAraG
BPHY_RS13305	-1,085	0,009288		Bphy_2623	RNB domain-containing ribonuclease
BPHY_RS13335	-1,278	0,007788		Bphy_2629	acetyl-CoA carboxylase biotin carboxyl carrierprotein
BPHY_RS13340	-1,749	0,000133	accC	Bphy_2630	acetyl-CoA carboxylase biotin carboxylasesubunit
BPHY_RS13345	-1,516	3,28E-05	prmA	Bphy_2631	50S ribosomal protein L11 methyltransferase
BPHY_RS13410	-2,288	0,001122		Bphy_2645	MarC family protein
BPHY_RS13430	-1,115	0,002284		Bphy_2649	glutamate 5-kinase
BPHY_RS13440	-2,007	0,000692	rpmA	Bphy_2651	50S ribosomal protein L27
BPHY_RS13445	-2,200	2,23E-05	rplU	Bphy_2652	50S ribosomal protein L21
BPHY_RS13465	-1,675	0,004578	tadA	Bphy_2655	Flp pilus assembly complex ATPase componentTadA
BPHY_RS13490	1,302	0,003787		Bphy_2660	DNA gyrase inhibitor YacG
BPHY_RS13610	-1,809	9,07E-05		Bphy_2684	porin
BPHY_RS13625	2,203	3,48E-10		Bphy_2687	M20 family metallopeptidase
BPHY_RS13740	-1,246	0,0013		Bphy_2703	uracil-DNA glycosylase
BPHY_RS13795	-1,376	0,001305		Bphy_2714	DUF748 domain-containing protein
BPHY_RS13835	-1,979	8,18E-05	prfA	Bphy_2721	peptide chain release factor 1
BPHY_RS13840	-1,720	0,000712	prmC	Bphy_2722	peptide chain release factor N(5)-glutaminemethyltransferase
BPHY_RS13860	-2,487	0,003528		Bphy_2726	APC family permease
BPHY_RS13865	-2,131	6,54E-05		Bphy_2727	cold-shock protein
BPHY_RS13870	-1,913	2,5E-05		Bphy_2728	Hsp70 family protein
BPHY_RS13885	-2,299	5,33E-06		Bphy_2731	MFS transporter
BPHY_RS13895	1,070	0,001826		Bphy_2733	DUF1415 domain-containing protein

BPHY_RS13945	1,623	1,36E-05		Bphy_2742	cytochrome bc complex cytochrome b subunit
BPHY_RS14025	-2,173	2,3E-05		Bphy_2758	histidinol-phosphate transaminase
BPHY_RS14040	-1,396	0,0047	murA	Bphy_2761	UDP-N-acetylglucosamine1-carboxyvinyltransferase
BPHY_RS14045	-1,383	0,00641		Bphy_2762	BolA family transcriptional regulator
BPHY_RS14050	-1,873	0,00119		Bphy_2763	ABC transporter permease
BPHY_RS14055	-1,631	8,37E-05		Bphy_2764	ABC transporter ATP-binding protein
BPHY_RS14130	-4,113	0,00122		Bphy_2779	flagellar basal body L-ring protein FlgH
BPHY_RS14155	-2,711	9,56E-05		Bphy_2784	HAMP domain-containing protein
BPHY_RS14170	-2,254	3,1E-08		Bphy_2787	glutamate synthase subunit beta
BPHY_RS14175	-2,154	1,65E-05		Bphy_2788	glutamate synthase subunit alpha
BPHY_RS14185	1,152	0,006787		Bphy_2790	outer membrane beta-barrel protein
BPHY_RS14190	2,215	2,54E-05		Bphy_2791	DUF883 family protein
BPHY_RS14225	-5,993	1,93E-06	pilM	Bphy_2798	pilus assembly protein PilM
BPHY_RS14240	-1,248	0,002425		Bphy_2801	lipoprotein
BPHY_RS14245	-1,654	0,005287	lysA	Bphy_2802	diaminopimelate decarboxylase
BPHY_RS14255	1,160	0,001648	msrP	Bphy_2804	protein-methionine-sulfoxide reductase catalyticsubunit MsrP
BPHY_RS14295	-2,745	5,76E-10	rplQ	Bphy_2812	50S ribosomal protein L17
BPHY_RS14300	-2,693	5,48E-07	rpoA	Bphy_2813	DNA-directed RNA polymerase subunit alpha
BPHY_RS14305	-2,898	4,18E-10	rpsD	Bphy_2814	30S ribosomal protein S4
BPHY_RS14310	-2,767	1,08E-07	rpsK	Bphy_2815	30S ribosomal protein S11
BPHY_RS14315	-2,649	1,01E-08	rpsM	Bphy_2816	30S ribosomal protein S13
BPHY_RS14320	-2,740	5,12E-06	rpmJ	Bphy_2817	50S ribosomal protein L36
BPHY_RS14325	-3,143	3,06E-07	infA	Bphy_2818	translation initiation factor IF-1
BPHY_RS14330	-3,568	9,54E-11	secY	Bphy_2819	preprotein translocase subunit SecY
BPHY_RS14335	-3,490	5,41E-09	rplO	Bphy_2820	50S ribosomal protein L15
BPHY_RS14340	-3,638	6,09E-10	rpmD	Bphy_2821	50S ribosomal protein L30
BPHY_RS14345	-2,892	6,75E-10	rpsE	Bphy_2822	30S ribosomal protein S5
BPHY_RS14350	-3,537	1,71E-11	rplR	Bphy_2823	50S ribosomal protein L18
BPHY_RS14355	-3,365	4,46E-10	rplF	Bphy_2824	50S ribosomal protein L6
BPHY_RS14360	-3,897	7,4E-11	rpsH	Bphy_2825	30S ribosomal protein S8
BPHY_RS14365	-3,861	1,35E-11	rpsN	Bphy_2826	30S ribosomal protein S14
BPHY_RS14370	-3,449	1,68E-10	rplE	Bphy_2827	50S ribosomal protein L5
BPHY_RS14375	-3,770	8,4E-10	rplX	Bphy_2828	50S ribosomal protein L24
BPHY_RS14380	-3,728	1,2E-08	rplN	Bphy_2829	50S ribosomal protein L14
BPHY_RS14385	-3,092	8,04E-13	rpsQ	Bphy_2831	30S ribosomal protein S17
BPHY_RS14390	-3,755	1,31E-18	rpmC	Bphy_2832	50S ribosomal protein L29
BPHY_RS14395	-3,224	1,02E-11	rplP	Bphy_2833	50S ribosomal protein L16
BPHY_RS14400	-3,053	3,16E-09	rpsC	Bphy_2834	30S ribosomal protein S3
BPHY_RS14405	-3,334	3,23E-09	rplV	Bphy_2835	50S ribosomal protein L22
BPHY_RS14410	-2,767	5,43E-09	rpsS	Bphy_2836	30S ribosomal protein S19
BPHY_RS14415	-3,313	1,47E-09	rplB	Bphy_2837	50S ribosomal protein L2
BPHY_RS14420	-3,233	3,4E-11	rplW	Bphy_2838	50S ribosomal protein L23
BPHY_RS14425	-3,310	1,3E-08	rplD	Bphy_2839	50S ribosomal protein L4

BPHY_RS14430	-3,437	2,69E-15	rplC	Bphy_2840	50S ribosomal protein L3
BPHY_RS14435	-2,511	2,29E-14	rpsJ	Bphy_2841	30S ribosomal protein S10
BPHY_RS14440	-2,389	4,6E-05	tuf	Bphy_2842	elongation factor Tu
BPHY_RS14445	-2,408	1,64E-07	fusA	Bphy_2843	elongation factor G
BPHY_RS14450	-2,316	6,27E-06	rpsG	Bphy_2844	30S ribosomal protein S7
BPHY_RS14455	-1,884	0,000219	rpsL	Bphy_2845	30S ribosomal protein S12
BPHY_RS14475	-2,421	1,02E-05	rplL	Bphy_2849	50S ribosomal protein L7/L12
BPHY_RS14480	-2,929	1,46E-08	rplJ	Bphy_2850	50S ribosomal protein L10
BPHY_RS14485	-2,408	4,2E-06	rplA	Bphy_2851	50S ribosomal protein L1
BPHY_RS14490	-3,094	7,06E-10	rplK	Bphy_2852	50S ribosomal protein L11
BPHY_RS14495	-2,112	4,07E-05	nusG	Bphy_2853	transcription termination/antiterminationprotein NusG
BPHY_RS14500	-1,643	0,006091	secE	Bphy_2854	preprotein translocase subunit SecE
BPHY_RS14595	1,544	0,004394		Bphy_2864	GNAT family N-acetyltransferase
BPHY_RS14610	-5,775	0,006058		Bphy_2867	hypothetical protein
BPHY_RS14615	2,128	1,42E-11		Bphy_2868	indolepyruvate ferredoxin oxidoreductase familyprotein
BPHY_RS14625	-1,614	0,005335		Bphy_2870	MFS transporter
BPHY_RS14640	1,641	3E-07		Bphy_2873	YbhB/YbcL family Raf kinase inhibitor-likeprotein
BPHY_RS14645	1,399	0,001062		Bphy_2874	flavodoxin family protein
BPHY_RS14660	-1,166	0,005517		Bphy_2877	NAD(P)-dependent oxidoreductase
BPHY_RS14675	-1,947	0,000756		Bphy_2880	hypothetical protein
BPHY_RS14685	1,679	0,001014		Bphy_2882	hypothetical protein
BPHY_RS14690	1,866	0,000209			hypothetical protein
BPHY_RS14720	1,427	0,001252		Bphy_2889	methyltransferase domain-containing protein
BPHY_RS14770	1,998	0,001842		Bphy_2899	complex I NDUFA9 subunit family protein
BPHY_RS14795	-1,722	0,006799		Bphy_2904	biotin-dependent carboxyltransferase familyprotein
BPHY_RS14820	-1,455	0,001426		Bphy_2909	TraB/GumN family protein
BPHY_RS14865	1,384	0,001064		Bphy_2918	adenosylhomocysteinase
BPHY_RS14875	2,340	9,44E-10		Bphy_2920	LrgB family protein
BPHY_RS14910	-3,184	0,000992		Bphy_2927	flagellar hook-length control protein FliK
BPHY_RS14935	-1,273	0,006074	fliF	Bphy_2931	flagellar M-ring protein FliF
BPHY_RS14940	-1,444	0,000439	fliG	Bphy_2932	flagellar motor switch protein FliG
BPHY_RS14950	-1,232	0,001908	fliI	Bphy_2934	flagellar protein export ATPase FliI
BPHY_RS14965	-1,447	0,005267	fliL	Bphy_2937	flagellar basal body-associated protein FliL
BPHY_RS14985	-2,041	0,000618	fliP	Bphy_2941	flagellar type III secretion system pore proteinFliP
BPHY_RS15015	-3,291	1,42E-09	flgA	Bphy_2947	flagellar basal body P-ring formation proteinFlgA
BPHY_RS15025	-1,838	0,00041	flgC	Bphy_2949	flagellar basal body rod protein FlgC
BPHY_RS15030	-1,851	0,000116	flgD	Bphy_2950	flagellar hook assembly protein FlgD
BPHY_RS15035	-1,692	0,001232		Bphy_2951	flagellar hook-basal body complex protein
BPHY_RS15040	-1,691	0,001118	flgF	Bphy_2952	flagellar basal-body rod protein FlgF
BPHY_RS15045	-1,603	0,006005	flgG	Bphy_2953	flagellar basal-body rod protein FlgG
BPHY_RS15050	-1,944	7,29E-05	flgH	Bphy_2954	flagellar basal body L-ring protein FlgH
BPHY_RS15055	-2,482	7,19E-08		Bphy_2955	flagellar basal body P-ring protein FlgI
BPHY_RS15060	-3,280	0,000638	flgJ	Bphy_2956	flagellar assembly peptidoglycan hydrolase FlgJ

BPHY_RS15065	-1,562	0,002557		Bphy_2957	flagellar brake protein
BPHY_RS15070	-1,491	0,000239	flgK	Bphy_2958	flagellar hook-associated protein FlgK
BPHY_RS15075	-1,229	0,006164	flgL	Bphy_2959	flagellar hook-associated protein FlgL
BPHY_RS15085	-2,751	2,5E-07		Bphy_2961	AAA family ATPase
BPHY_RS15090	-1,953	1,35E-06	flhF	Bphy_2962	flagellar biosynthesis protein FlhF
BPHY_RS15095	-1,974	2,93E-05	flhA	Bphy_2963	flagellar biosynthesis protein FlhA
BPHY_RS15100	-1,968	0,004569	flhB	Bphy_2964	flagellar type III secretion system proteinFlhB
BPHY_RS15115	-2,294	9,68E-06	cheZ	Bphy_2967	protein phosphatase CheZ
BPHY_RS15120	-1,565	0,004738	cheY	Bphy_2968	chemotaxis response regulator CheY
BPHY_RS15125	-1,548	0,001791		Bphy_2969	chemotaxis response regulator protein-glutamatemethylesterase
BPHY_RS15130	-3,560	0,001644	cheD	Bphy_2970	chemoreceptor glutamine deamidase CheD
BPHY_RS15170	-2,299	0,002349	flhC	Bphy_2978	flagellar transcriptional regulator FlhC
BPHY_RS15180	1,831	2,07E-05		Bphy_2980	glycosyltransferase family 4 protein
BPHY_RS15215	-1,804	0,002005		Bphy_2987	30S ribosomal protein S21
BPHY_RS15220	1,061	0,002068		Bphy_2988	aldo/keto reductase
BPHY_RS15230	-1,135	0,00167	fliD	Bphy_2990	flagellar filament capping protein FliD
BPHY_RS15240	-1,838	2,13E-06		Bphy_2992	glycosyltransferase family 41 protein
BPHY_RS15245	-1,931	4,2E-07		Bphy_2993	glycosyltransferase family 41 protein
BPHY_RS15250	-1,555	0,003092		Bphy_2994	class I SAM-dependent methyltransferase
BPHY_RS15255	-2,248	1,29E-05		Bphy_2995	SDR family oxidoreductase
BPHY_RS15260	-2,088	5,25E-06		Bphy_2996	tetratricopeptide repeat protein
BPHY_RS15265	-1,472	0,000828		Bphy_2997	tetratricopeptide repeat protein
BPHY_RS15285	-1,423	6,55E-05		Bphy_3000	UvrD-helicase domain-containing protein
BPHY_RS15320	-1,261	0,008405		Bphy_3006	class I SAM-dependent methyltransferase
BPHY_RS15325	-1,764	0,000324		Bphy_3007	class I SAM-dependent methyltransferase
BPHY_RS15330	-1,674	0,000167	pseI	Bphy_3008	pseudaminic acid synthase
BPHY_RS15370	1,260	0,00123		Bphy_3016	thiamine pyrophosphate-binding protein
BPHY_RS15380	-1,729	2,55E-06	putA	Bphy_3018	trifunctional transcriptional regulator/proline dehydrogenase/L-glutamate gamma-semialdehyde dehydrogenase
BPHY_RS15390	-1,471	0,003995		Bphy_3021	putative sulfate exporter family transporter
BPHY_RS15395	-1,636	0,001942		Bphy_3022	primosomal protein N'
BPHY_RS15415	-1,989	0,000447		Bphy_3026	F0F1 ATP synthase subunit epsilon
BPHY_RS15420	-2,046	0,000139	atpD	Bphy_3027	F0F1 ATP synthase subunit beta
BPHY_RS15425	-2,146	6,47E-06	atpG	Bphy_3028	F0F1 ATP synthase subunit gamma
BPHY_RS15430	-2,957	3,9E-07	atpA	Bphy_3029	F0F1 ATP synthase subunit alpha
BPHY_RS15435	-2,711	7,3E-06		Bphy_3030	F0F1 ATP synthase subunit delta
BPHY_RS15440	-2,691	2,67E-08		Bphy_3031	F0F1 ATP synthase subunit B
BPHY_RS15445	-2,498	5,57E-08	atpE	Bphy_3032	F0F1 ATP synthase subunit C
BPHY_RS15450	-2,156	4,78E-05	atpB	Bphy_3033	F0F1 ATP synthase subunit A
BPHY_RS15500	-2,072	0,000186		Bphy_3043	ABC transporter substrate-binding protein
BPHY_RS15515	-2,580	0,001188		Bphy_3046	branched-chain amino acid ABC transporterpermease
BPHY_RS15550	1,650	0,000216		Bphy_3053	class IV adenylate cyclase

BPHY_RS15590	-1,464	0,002148		Bphy_3061	MOSC domain-containing protein
BPHY_RS15605	-1,629	0,001331		Bphy_3064	glutamate--cysteine ligase
BPHY_RS15610	-1,672	5,77E-05		Bphy_3065	cation:proton antiporter
BPHY_RS15690	-1,279	0,001181		Bphy_3081	type II secretion system protein N
BPHY_RS15700	-1,243	0,004874		Bphy_3084	OmpA family protein
BPHY_RS15720	1,294	0,000145	ada	Bphy_3088	bifunctional DNA-binding transcriptional regulator/O6-methylguanine-DNA methyltransferase Ada
BPHY_RS15750	2,550	4,15E-12		Bphy_3094	hypothetical protein
BPHY_RS15770	-1,848	1,65E-05	yidC	Bphy_3098	membrane protein insertase YidC
BPHY_RS15775	-2,206	9,12E-05	rnpA	Bphy_3100	ribonuclease P protein component
BPHY_RS15780	-2,395	3,63E-05	rpmH	Bphy_3101	50S ribosomal protein L34
BPHY_RS15920	-2,285	0,000611		Bphy_3127	glycoside hydrolase family 31 protein
BPHY_RS15925	-3,517	0,00025		Bphy_3128	MFS transporter
BPHY_RS16255	-7,261	7,37E-06		Bphy_3200	PDR/VanB family oxidoreductase
BPHY_RS16285	-4,437	0,00919	ligA	Bphy_3205	protocatechuate 4,5-dioxygenase subunit alpha
BPHY_RS16300	-6,414	0,004748		Bphy_3208	amidohydrolase
BPHY_RS16385	-2,624	0,001176		Bphy_3226	Nramp family divalent metal transporter
BPHY_RS16455	3,605	4,25E-17		Bphy_3241	CBS domain-containing protein
BPHY_RS16460	2,843	1,27E-09		Bphy_3242	DUF4148 domain-containing protein
BPHY_RS16470	2,779	1,16E-12		Bphy_3244	BON domain-containing protein
BPHY_RS16490	-3,427	0,0026		Bphy_3248	amino acid ABC transporter permease
BPHY_RS16505	1,597	5,29E-07		Bphy_3251	xanthine dehydrogenase family proteinmolybdopterin-binding subunit
BPHY_RS16510	1,537	0,00027		Bphy_3252	(2Fe-2S)-binding protein
BPHY_RS16520	-1,531	0,003876	bcsZ	Bphy_3254	cellulase
BPHY_RS16525	-1,162	0,001971	bcsB	Bphy_3255	cellulose biosynthesis cyclic di-GMP-bindingregulatory protein BcsB
BPHY_RS16530	-1,358	0,001288	bcsA	Bphy_3256	UDP-forming cellulose synthase catalyticsubunit
BPHY_RS16550	1,548	0,000339		Bphy_3260	hypothetical protein
BPHY_RS16555	1,049	0,009788		Bphy_3261	alpha/beta hydrolase
BPHY_RS16690	1,164	0,005331		Bphy_3291	LysR family transcriptional regulator
BPHY_RS16705	1,801	6,89E-05		Bphy_3294	glycine betaine ABC transportersubstrate-binding protein
BPHY_RS16760	3,422	1,34E-22		Bphy_3305	adenosylcobalamin-dependentribonucleoside-diphosphate reductase
BPHY_RS16835	1,634	2,52E-06		Bphy_3320	alpha/beta hydrolase
BPHY_RS16865	-7,827	1,15E-06		Bphy_3326	LysR family transcriptional regulator
BPHY_RS16895	-5,962	0,00321		Bphy_3332	sarcosine oxidase subunit delta
BPHY_RS16900	-2,134	0,002623		Bphy_3333	sarcosine oxidase subunit beta family protein
BPHY_RS16915	-3,047	0,001558		Bphy_3336	DMT family transporter
BPHY_RS16940	1,123	0,008549	zwf	Bphy_3342	glucose-6-phosphate dehydrogenase
BPHY_RS17030	-2,647	0,009368		Bphy_3360	efflux transporter outer membrane subunit
BPHY_RS17060	1,748	0,001224		Bphy_3366	putative glycolipid-binding domain-containingprotein
BPHY_RS17130	-5,822	0,007174		Bphy_3379	hypothetical protein
BPHY_RS17145	-2,060	0,008799		Bphy_3382	tail protein
BPHY_RS17160	-6,336	0,000938		Bphy_3385	phage minor tail protein L
BPHY_RS17170	-4,441	0,000913		Bphy_3387	hypothetical protein

BPHY_RS17355	-1,916	0,000795		Bphy_3423	site-specific DNA-methyltransferase
BPHY_RS17365	1,588	0,002052		Bphy_3425	DUF1488 domain-containing protein
BPHY_RS17455	-2,079	0,003576		Bphy_3444	cyanate transporter
BPHY_RS17465	-1,935	0,000963		Bphy_3446	ABC transporter substrate-binding protein
BPHY_RS17515	-2,148	0,005504		Bphy_3456	hypothetical protein
BPHY_RS17545	-4,286	8,57E-05		Bphy_3463	sugar ABC transporter ATP-binding protein
BPHY_RS17635	-2,002	0,00887		Bphy_3482	LPS-assembly protein LptD
BPHY_RS17640	-6,212	0,001423		Bphy_3483	hypothetical protein
BPHY_RS17655	-2,208	0,009462		Bphy_3486	efflux transporter outer membrane subunit
BPHY_RS17665	-2,267	0,003905		Bphy_3488	efflux RND transporter permease subunit
BPHY_RS17675	-3,082	0,000813		Bphy_3490	efflux transporter outer membrane subunit
BPHY_RS17680	-3,387	0,000145		Bphy_3491	efflux RND transporter permease subunit
BPHY_RS17685	-5,004	9,01E-05		Bphy_3492	efflux RND transporter periplasmic adaptorsubunit
BPHY_RS17700	1,704	0,005001		Bphy_3495	HAMP domain-containing protein
BPHY_RS17705	3,903	1,42E-08		Bphy_3496	hypothetical protein
BPHY_RS17710	2,639	9,95E-08		Bphy_3497	ATP-binding cassette domain-containing protein
BPHY_RS17715	2,806	5,87E-08		Bphy_3498	ABC transporter permease
BPHY_RS17720	2,424	7,5E-09		Bphy_3499	MlaD family protein
BPHY_RS17725	2,549	6,24E-07		Bphy_3500	PqiC family protein
BPHY_RS17730	1,583	0,000181		Bphy_3501	amino acid permease
BPHY_RS17735	2,449	6,79E-08	ppk2	Bphy_3502	polyphosphate kinase 2
BPHY_RS17740	2,466	1,37E-09		Bphy_3503	NAD-binding protein
BPHY_RS17745	1,386	8E-05		Bphy_3504	SulP family inorganic anion transporter
BPHY_RS17760	1,767	0,000585		Bphy_3507	TolC family protein
BPHY_RS17770	2,281	5,84E-05		Bphy_3509	outer membrane beta-barrel protein
BPHY_RS17840	-2,378	0,001683		Bphy_3525	oxidoreductase
BPHY_RS17870	-2,134	0,003833		Bphy_3532	VOC family protein
BPHY_RS17895	-2,413	0,000395		Bphy_3537	efflux transporter outer membrane subunit
BPHY_RS17900	-2,181	0,000538		Bphy_3538	efflux RND transporter permease subunit
BPHY_RS17940	-2,051	9,22E-05		Bphy_3547	efflux RND transporter periplasmic adaptorsubunit
BPHY_RS17950	-1,632	0,002289		Bphy_3549	TolC family protein
BPHY_RS18090	3,089	8,74E-14		Bphy_3578	fructose bisphosphate aldolase
BPHY_RS18100	1,469	0,002099		Bphy_3580	DUF2970 domain-containing protein
BPHY_RS18150	-1,795	0,004775		Bphy_3590	MCP four helix bundle domain-containing protein
BPHY_RS18175	-2,065	0,002836		Bphy_3595	ROK family transcriptional regulator
BPHY_RS18190	1,241	0,006106		Bphy_3598	TetR family transcriptional regulator
BPHY_RS18195	-2,070	0,001638		Bphy_3599	EAL domain-containing protein
BPHY_RS18200	-5,144	0,001103		Bphy_3600	cupin domain-containing protein
BPHY_RS18215	-1,912	0,001248		Bphy_3603	ABC transporter substrate-binding protein
BPHY_RS18240	1,433	0,009934		Bphy_3608	hypothetical protein
BPHY_RS18245	1,709	1,38E-07		Bphy_3609	DUF72 domain-containing protein
BPHY_RS18250	1,974	1,26E-08		Bphy_3610	SDR family oxidoreductase
BPHY_RS18255	1,540	0,000435		Bphy_3611	porin

BPHY_RS18435	5,330	1,41E-31	cyoD	Bphy_3646	cytochrome o ubiquinol oxidase subunit IV
BPHY_RS18440	5,830	2,17E-53	cyoC	Bphy_3647	cytochrome o ubiquinol oxidase subunit III
BPHY_RS18445	5,561	1,41E-82	cyoB	Bphy_3648	cytochrome o ubiquinol oxidase subunit I
BPHY_RS18450	4,493	5,52E-38	cyoA	Bphy_3649	ubiquinol oxidase subunit II
BPHY_RS18470	-6,598	0,00278		Bphy_3653	septal ring lytic transglycosylase RlpA familyprotein
BPHY_RS18490	1,084	0,00219	ahpC	Bphy_3657	peroxiredoxin
BPHY_RS18515	1,889	1,95E-06		Bphy_3664	SulP family inorganic anion transporter
BPHY_RS18520	2,134	9,91E-07		Bphy_3665	carbonic anhydrase
BPHY_RS18525	2,119	2,58E-05		Bphy_3666	DUF427 domain-containing protein
BPHY_RS18540	2,687	6,07E-09	wrbA	Bphy_3669	NAD(P)H:quinone oxidoreductase
BPHY_RS18570	4,518	1,9E-31		Bphy_3675	hypothetical protein
BPHY_RS18575	4,236	6,94E-16		Bphy_3676	hypothetical protein
BPHY_RS18580	3,586	1,1E-06		Bphy_3677	DUF2964 family protein
BPHY_RS18615	4,949	2,27E-40		Bphy_3684	DUF3141 domain-containing protein
BPHY_RS18620	5,383	3,6E-61		Bphy_3685	phosphate acetyltransferase
BPHY_RS18625	5,603	8,81E-64		Bphy_3686	acetate/propionate family kinase
BPHY_RS18630	5,602	5,81E-73	fabI	Bphy_3687	enoyl-ACP reductase FabI
BPHY_RS18640	-2,352	8,62E-07	rpoD	Bphy_3690	RNA polymerase sigma factor RpoD
BPHY_RS18645	1,723	1,17E-06		Bphy_3691	PRC-barrel domain-containing protein
BPHY_RS18680	1,337	0,004015		Bphy_3698	CBS domain-containing protein
BPHY_RS18750	1,706	0,000112		Bphy_3712	efflux RND transporter periplasmic adaptorsubunit
BPHY_RS18770	1,556	4,43E-05		Bphy_3716	S8 family serine peptidase
BPHY_RS18825	-4,284	0,000551		Bphy_3727	phosphatase PAP2 family protein
BPHY_RS18835	-2,533	0,000358		Bphy_3729	lipopolysaccharide biosynthesis protein
BPHY_RS18870	-3,539	3,95E-10		Bphy_3736	hypothetical protein
BPHY_RS18895	2,001	1,31E-07		Bphy_3740	NAD(P)/FAD-dependent oxidoreductase
BPHY_RS18900	1,218	0,00021		Bphy_3741	leucine-rich repeat domain-containing protein
BPHY_RS18905	3,543	7,95E-18		Bphy_3742	hypothetical protein
BPHY_RS18920	1,737	3,94E-07		Bphy_3746	VOC family protein
BPHY_RS18975	-2,892	0,000373		Bphy_3756	c-type cytochrome
BPHY_RS19000	-7,221	2,73E-06		Bphy_3761	NAD(+)/NADH kinase
BPHY_RS19005	1,295	0,009752		Bphy_3762	sigma-54-dependent Fis family transcriptionalregulator
BPHY_RS19060	-7,688	1,29E-06	phnG	Bphy_3773	phosphonate C-P lyase system protein PhnG
BPHY_RS19105	-2,103	0,001662		Bphy_3782	porin
BPHY_RS19130	-2,083	0,001295		Bphy_3787	DUF6232 family protein
BPHY_RS19145	2,292	5,63E-13		Bphy_3790	glycogen/starch/alpha-glucan phosphorylase
BPHY_RS19175	-2,446	0,001436		Bphy_3796	CPBP family intramembrane metalloprotease
BPHY_RS19190	-3,902	0,001009	oxlT	Bphy_3800	oxalate/formate MFS antiporter
BPHY_RS19195	-2,863	0,001199		Bphy_3801	MoxR family ATPase
BPHY_RS19210	-3,330	0,001675		Bphy_3804	VWA domain-containing protein
BPHY_RS19265	-2,780	0,00788		Bphy_3815	aldehyde dehydrogenase family protein
BPHY_RS19270	-7,493	1,98E-05		Bphy_3816	porin
BPHY_RS19305	-2,023	0,001719		Bphy_3823	methyl-accepting chemotaxis protein

BPHY_RS19325	-6,756	0,000108		Bphy_3827	hypothetical protein
BPHY_RS19340	-7,091	0,000602		Bphy_3830	lecithin retinol acyltransferase family protein
BPHY_RS19385	2,469	7,99E-06		Bphy_3839	hypothetical protein
BPHY_RS19390	2,250	0,008487		Bphy_3840	DUF4399 domain-containing protein
BPHY_RS19395	2,486	2,28E-08		Bphy_3841	DUF4399 domain-containing protein
BPHY_RS19410	4,913	7,82E-43		Bphy_3844	hypothetical protein
BPHY_RS19415	3,297	6,64E-15		Bphy_3845	hypothetical protein
BPHY_RS19420	1,800	0,002889		Bphy_3846	hypothetical protein
BPHY_RS19435	1,303	0,000699		Bphy_3850	H-NS histone family protein
BPHY_RS19460	1,102	0,004461	ppk2	Bphy_3855	polyphosphate kinase 2
BPHY_RS19490	-1,232	0,009734	doeB	Bphy_3861	N(2)-acetyl-L-2,4-diaminobutanoate deacetylaseDoeB
BPHY_RS19505	-3,574	0,002044	eutB	Bphy_3864	hydroxyectoine utilization dehydratase EutB
BPHY_RS19540	-1,426	0,003515		Bphy_3871	phosphocholine cytidylyltransferase familyprotein
BPHY_RS19545	-1,132	0,004404		Bphy_3872	flippase-like domain-containing protein
BPHY_RS19645	1,908	1,98E-05		Bphy_3894	hypothetical protein
BPHY_RS19655	1,187	0,003739		Bphy_3897	hypothetical protein
BPHY_RS19665	-2,039	2E-08		Bphy_3899	transglycosylase domain-containing protein
BPHY_RS19670	-1,163	0,00642		Bphy_3900	sodium:solute symporter
BPHY_RS19685	1,537	0,004665		Bphy_3903	pirin family protein
BPHY_RS19690	1,978	1,32E-05		Bphy_3904	DoxX family protein
BPHY_RS19695	-3,207	8,81E-09		Bphy_3905	porin
BPHY_RS19740	2,061	5,86E-09		Bphy_3915	phasin family protein
BPHY_RS19770	2,191	5,51E-06		Bphy_3921	alpha/beta hydrolase
BPHY_RS19775	-2,452	0,004605		Bphy_3922	MFS transporter
BPHY_RS19790	-2,452	0,007002		Bphy_3925	ADP-heptose--LPS heptosyltransferase
BPHY_RS19820	-1,354	0,001782		Bphy_3932	hypothetical protein
BPHY_RS19830	1,974	6,3E-06		Bphy_3934	acyl-CoA dehydrogenase
BPHY_RS19880	1,395	0,000352		Bphy_3944	30S ribosomal protein S21
BPHY_RS19885	-1,291	0,001009		Bphy_3945	NAD(P)/FAD-dependent oxidoreductase
BPHY_RS19890	-2,172	2,13E-06	tsaD	Bphy_3946	yltransferase complextransferase subunit TsaD
BPHY_RS19935	-2,580	2,46E-06		Bphy_3955	EAL domain-containing protein
BPHY_RS19945	-1,601	0,004777		Bphy_3957	sensor histidine kinase
BPHY_RS19960	-1,567	0,006556		Bphy_3960	ABC transporter ATP-binding protein
BPHY_RS19965	-2,060	0,003558		Bphy_3961	ABC transporter permease
BPHY_RS19970	1,064	0,003645		Bphy_3962	heavy metal response regulator transcriptionfactor
BPHY_RS19980	-2,128	0,009239		Bphy_3964	two-component sensor histidine kinase
BPHY_RS20000	-3,580	1,68E-12	cyoA	Bphy_3968	ubiquinol oxidase subunit II
BPHY_RS20005	-5,298	7,28E-23	cyoB	Bphy_3969	cytochrome o ubiquinol oxidase subunit I
BPHY_RS20015	2,512	1,55E-07	cyoD	Bphy_3971	cytochrome o ubiquinol oxidase subunit IV
BPHY_RS20020	3,453	3,95E-12		Bphy_3972	hypothetical protein
BPHY_RS20025	-2,719	1,56E-09		Bphy_3973	NADH-quinone oxidoreductase subunit M
BPHY_RS20055	-2,037	0,005866	uca	Bphy_3979	urea carboxylase
BPHY_RS20065	1,834	2,65E-05		Bphy_3981	antibiotic biosynthesis monooxygenase

BPHY_RS20110	1,541	2,75E-05		Bphy_3990	hypothetical protein
BPHY_RS20160	1,193	0,000258		Bphy_4001	catalase
BPHY_RS20165	1,349	0,003565		Bphy_4002	MurR/RpiR family transcriptional regulator
BPHY_RS20170	1,694	0,00106	ddpX	Bphy_4003	D-alanyl-D-alanine dipeptidase
BPHY_RS20175	2,086	8,25E-08		Bphy_4004	ABC transporter substrate-binding protein
BPHY_RS20215	-7,881	1,93E-06		Bphy_4012	SDR family oxidoreductase
BPHY_RS20245	-3,319	3,47E-05		Bphy_4018	virulence factor family protein
BPHY_RS20250	-2,704	0,000167	mprF	Bphy_4019	bifunctional lysylphosphatidylglycerolflippase/synthetase MprF
BPHY_RS20265	-2,823	0,004135		Bphy_4022	efflux transporter outer membrane subunit
BPHY_RS20270	-6,314	0,006213		Bphy_4023	MarR family transcriptional regulator
BPHY_RS20310	1,915	1,12E-07		Bphy_4031	DUF3331 domain-containing protein
BPHY_RS20330	-4,133	1,43E-07		Bphy_4035	acetyltransferase
BPHY_RS20335	-3,709	7,62E-07		Bphy_4036	lysine N(6)-hydroxylase/L-ornithineN(5)-oxygenase family protein
BPHY_RS20340	-3,775	1,59E-05		Bphy_4037	acetyltransferase
BPHY_RS20345	-3,157	2,61E-06		Bphy_4038	non-ribosomal peptide synthetase
BPHY_RS20350	-4,077	7,39E-09		Bphy_4039	non-ribosomal peptide synthetase
BPHY_RS20355	-5,528	4,03E-14		Bphy_4040	cyclic peptide export ABC transporter
BPHY_RS20360	-3,518	3,97E-07		Bphy_4041	iron-siderophore ABC transportersubstrate-binding protein
BPHY_RS20365	-4,125	2,45E-06	fhuF	Bphy_4042	siderophore-iron reductase FhuF
BPHY_RS20370	-2,970	1,6E-09	fhuB	Bphy_4043	Fe(3+)-hydroxamate ABC transporter permeaseFhuB
BPHY_RS20375	-4,715	2,77E-11		Bphy_4044	ABC transporter ATP-binding protein
BPHY_RS20380	-5,441	6,93E-16		Bphy_4045	TauD/TfdA family dioxygenase
BPHY_RS20385	-5,238	1,18E-11		Bphy_4046	MbtH family NRPS accessory protein
BPHY_RS20390	-5,832	6,71E-18		Bphy_4047	RNA polymerase factor sigma-70
BPHY_RS20395	-3,730	1,86E-05		Bphy_4048	MFS transporter
BPHY_RS20415	-2,250	0,00245		Bphy_4052	IclR family transcriptional regulator
BPHY_RS20440	1,643	9,78E-06		Bphy_4057	sugar ABC transporter substrate-binding protein
BPHY_RS20465	-1,370	0,005358		Bphy_4062	hypothetical protein
BPHY_RS20550	1,856	4,5E-07		Bphy_4079	hypothetical protein
BPHY_RS20580	1,241	0,005211		Bphy_4084	VOC family protein
BPHY_RS20660	1,726	3,61E-05		Bphy_4100	YciI family protein
BPHY_RS20670	1,431	0,001068		Bphy_4102	SRPBCC family protein
BPHY_RS20675	1,571	1,6E-06		Bphy_4103	aromatic ring-hydroxylating dioxygenase subunitalpha
BPHY_RS20690	2,512	1,66E-12		Bphy_4107	glutathione S-transferase family protein
BPHY_RS20710	1,208	0,009996		Bphy_4111	SRPBCC family protein
BPHY_RS20735	1,678	0,000449		Bphy_4116	rubrerythrin family protein
BPHY_RS20740	1,495	0,000493		Bphy_4117	DUF3564 domain-containing protein
BPHY_RS20745	-1,883	0,001711		Bphy_4118	DNA/RNA non-specific endonuclease
BPHY_RS20765	1,481	7,19E-07	egtD	Bphy_4124	L-histidine N(alpha)-methyltransferase
BPHY_RS20775	-2,337	8,73E-06		Bphy_4126	NAD-dependent epimerase/dehydratase familyprotein
BPHY_RS20830	-2,078	0,001091		Bphy_4136	phosphorylase
BPHY_RS20835	-1,508	0,001227	shc	Bphy_4137	squalene--hopene cyclase
BPHY_RS20880	1,147	0,005249		Bphy_4146	NAD(P)H-dependent oxidoreductase

BPHY_RS20990	-5,762	0,007923		Bphy_4171	hypothetical protein
BPHY_RS21000	2,385	9,68E-05	hpnR	Bphy_4173	hopanoid C-3 methylase HpnR
BPHY_RS21065	-5,148	0,003643		Bphy_4185	cytochrome b
BPHY_RS21085	1,945	2,2E-06		Bphy_4189	selenium-binding family protein
BPHY_RS21145	1,519	0,00025		Bphy_4201	GlxA family transcriptional regulator
BPHY_RS21150	3,921	4,43E-24		Bphy_4203	hypothetical protein
BPHY_RS21190	1,950	0,000299		Bphy_4211	hypothetical protein
BPHY_RS21205	1,451	1,2E-05		Bphy_4214	FadR family transcriptional regulator
BPHY_RS21230	-2,514	0,002416	araH	Bphy_4219	L-arabinose ABC transporter permease AraH
BPHY_RS21265	-1,511	0,002952		Bphy_4226	dipeptide ABC transporter ATP-binding protein
BPHY_RS21275	-1,182	0,005285		Bphy_4228	MurR/RpiR family transcriptional regulator
BPHY_RS21295	-7,249	7,83E-06		Bphy_4232	hypothetical protein
BPHY_RS21325	-2,011	0,007375		Bphy_4238	SDR family oxidoreductase
BPHY_RS21335	-1,943	0,009713		Bphy_4240	aspartate dehydrogenase
BPHY_RS21345	-1,905	0,008658		Bphy_4242	VOC family protein
BPHY_RS21350	-3,763	4,33E-05		Bphy_4243	FAD-dependent oxidoreductase
BPHY_RS21380	4,706	1,73E-28		Bphy_4249	CoA transferase
BPHY_RS21385	4,679	1,64E-13		Bphy_4250	hydroxymethylglutaryl-CoA lyase
BPHY_RS21415	-4,039	0,002015		Bphy_4255	2-keto-4-pentenoate hydratase
BPHY_RS21485	-1,575	0,001264		Bphy_4269	methyl-accepting chemotaxis protein
BPHY_RS21500	-1,941	0,008783		Bphy_4272	EAL domain-containing protein
BPHY_RS21565	-2,093	1,13E-06		Bphy_4287	peptide chain release factor 3
BPHY_RS21580	-2,635	4,18E-05		Bphy_4290	hypothetical protein
BPHY_RS21590	-4,209	1,91E-05		Bphy_4292	ShlB/FhaC/HecB family hemolysinsecretion/activation protein
BPHY_RS21595	1,217	0,009803		Bphy_4293	hypothetical protein
BPHY_RS21655	-2,432	0,000273		Bphy_4305	Tar ligand binding domain-containing protein
BPHY_RS21670	-1,196	0,007772		Bphy_4308	hypothetical protein
BPHY_RS21690	-2,254	0,005956	phaZ	Bphy_4313	polyhydroxyalkanoate depolymerase
BPHY_RS21770	-1,650	0,005588		Bphy_4329	HlyD family secretion protein
BPHY_RS21790	-1,594	0,009141		Bphy_4333	response regulator
BPHY_RS21800	-6,073	0,001898		Bphy_4335	response regulator transcription factor
BPHY_RS21820	-3,186	9E-05		Bphy_4338	YadA-like family protein
BPHY_RS21845	-5,698	0,009821		Bphy_4343	hypothetical protein
BPHY_RS21855	-3,125	0,000185		Bphy_4345	pilus assembly protein N-terminaldomain-containing protein
BPHY_RS21860	-1,363	0,006861	tadA	Bphy_4346	Flp pilus assembly complex ATPase componentTadA
BPHY_RS21865	-4,536	0,002175		Bphy_4347	type II secretion system F family protein
BPHY_RS21885	-7,630	9,58E-07		Bphy_4351	lytic transglycosylase domain-containingprotein
BPHY_RS21980	-1,485	0,001158	asd	Bphy_4370	aspartate-semialdehyde dehydrogenase
BPHY_RS22005	-1,360	0,00454		Bphy_4375	site-specific DNA-methyltransferase
BPHY_RS22065	1,656	1,77E-05	treY	Bphy_4387	malto-oligosyltrehalose synthase
BPHY_RS22075	2,085	1,21E-12	treZ	Bphy_4389	malto-oligosyltrehalose trehalohydrolase
BPHY_RS22080	1,794	1,07E-05	glgX	Bphy_4390	glycogen debranching protein GlgX
BPHY_RS22085	1,413	0,00037	glgB	Bphy_4391	1,4-alpha-glucan branching protein GlgB

BPHY_RS22090	1,801	7,94E-07	treS	Bphy_4392	maltose alpha-D-glucosyltransferase
BPHY_RS22095	1,567	4,61E-06		Bphy_4393	DUF3416 domain-containing protein
BPHY_RS22135	-1,371	0,009806		Bphy_4399	hypothetical protein
BPHY_RS22145	1,344	0,000201		Bphy_4401	tautomerase family protein
BPHY_RS22175	1,354	0,001908		Bphy_4407	PHB depolymerase family esterase
BPHY_RS22205	1,362	0,00012		Bphy_4414	AMP nucleosidase
BPHY_RS22250	-2,270	8,5E-05		Bphy_4423	NAD(P)-dependent oxidoreductase
BPHY_RS22255	-2,124	0,00011		Bphy_4424	four-carbon acid sugar kinase family protein
BPHY_RS22260	-1,935	0,004821		Bphy_4425	aldolase
BPHY_RS22305	1,744	2,27E-05		Bphy_4434	universal stress protein
BPHY_RS22310	1,207	0,003374		Bphy_4435	FadR family transcriptional regulator
BPHY_RS22320	2,058	1,61E-07		Bphy_4437	GNAT family N-acetyltransferase
BPHY_RS22325	1,352	0,000211		Bphy_4438	helix-turn-helix transcriptional regulator
BPHY_RS22330	1,149	0,004967		Bphy_4439	LuxR family transcriptional regulator
BPHY_RS22360	-7,740	3,79E-06		Bphy_4445	sugar ABC transporter permease
BPHY_RS22380	1,938	1,27E-06		Bphy_4449	DUF3005 domain-containing protein
BPHY_RS22390	1,192	0,008483		Bphy_4451	serine acetyltransferase
BPHY_RS22405	1,027	0,00691	folE	Bphy_4454	GTP cyclohydrolase I FolE
BPHY_RS22465	2,217	2,89E-05		Bphy_4466	hypothetical protein
BPHY_RS22475	2,432	2,38E-06		Bphy_4468	aspartate aminotransferase family protein
BPHY_RS22570	2,449	3,79E-14		Bphy_4487	phasin family protein
BPHY_RS22575	1,346	0,000679		Bphy_4488	putative zinc-binding metallopeptidase
BPHY_RS22600	-5,984	0,002504		Bphy_4493	MarR family winged helix-turn-helixtranscriptional regulator
BPHY_RS22605	-6,157	6,82E-05		Bphy_4494	HlyD family secretion protein
BPHY_RS22615	-2,654	0,003578		Bphy_4496	efflux transporter outer membrane subunit
BPHY_RS22645	-3,923	0,001116		Bphy_4502	ABC transporter ATP-binding protein
BPHY_RS22660	-2,306	0,002571		Bphy_4505	peptide ABC transporter substrate-bindingprotein
BPHY_RS22670	-2,190	0,0002		Bphy_4508	oligopeptide:H+ symporter
BPHY_RS22685	2,372	2,09E-11		Bphy_4511	circularly permuted type 2 ATP-grasp protein
BPHY_RS22690	2,478	6,22E-10		Bphy_4512	alpha-E domain-containing protein
BPHY_RS22720	2,308	1,15E-14	fdnG	Bphy_4518	formate dehydrogenase-N subunit alpha
BPHY_RS22730	2,388	3,74E-05		Bphy_4520	formate dehydrogenase subunit gamma
BPHY_RS22735	1,335	0,000954	fdhE	Bphy_4521	formate dehydrogenase accessory protein FdhE
BPHY_RS22755	1,874	0,000115		Bphy_4525	cupin domain-containing protein
BPHY_RS22810	-5,796	0,000561		Bphy_4536	3-carboxyethylcatechol 2,3-dioxygenase
BPHY_RS22820	-3,128	0,007664		Bphy_4538	acetaldehyde dehydrogenase (acetylating)
BPHY_RS22825	-3,452	0,00074	dmpG	Bphy_4539	4-hydroxy-2-oxovalerate aldolase
BPHY_RS22830	-3,660	0,002185	mhpT	Bphy_4540	3-(3-hydroxy-phenyl)propionate transporter MhpT
BPHY_RS22865	-2,785	0,007523		Bphy_4547	xanthine dehydrogenase family proteinmolybdopterin-binding subunit
BPHY_RS22880	-5,055	0,000794		Bphy_4550	SRPBCC family protein
BPHY_RS22890	-6,689	0,002684	hpxZ	Bphy_4552	oxalurate catabolism protein HpxZ
BPHY_RS22895	-3,235	0,004512		Bphy_4553	amidase
BPHY_RS22910	-6,310	7,89E-05		Bphy_4556	amino acid ABC transporter permease

BPHY_RS22930	-4,690	0,003347		Bphy_4560	MSMEG_0572 family nitrogen starvation responseprotein
BPHY_RS22940	-2,758	0,009314		Bphy_4562	MSMEG_0568 family radical SAM protein
BPHY_RS22955	-2,988	0,00047		Bphy_4565	MSMEG_0565 family glycosyltransferase
BPHY_RS22970	-3,078	0,003509		Bphy_4568	hypothetical protein
BPHY_RS23010	-2,017	0,009881		Bphy_4576	FAD-dependent oxidoreductase
BPHY_RS23020	2,604	8,14E-05		Bphy_4578	amino acid ABC transporter substrate-bindingprotein
BPHY_RS23040	1,437	5,6E-05		Bphy_4582	DUF4382 domain-containing protein
BPHY_RS23045	1,510	0,002426		Bphy_4585	mechanosensitive ion channel family protein
BPHY_RS23050	1,287	0,009422		Bphy_4586	hypothetical protein
BPHY_RS23055	1,878	4,08E-05		Bphy_4587	H-NS histone family protein
BPHY_RS23080	-5,241	0,000279		Bphy_4592	MFS transporter
BPHY_RS23085	-2,418	0,007758		Bphy_4593	dihydrodipicolinate synthase family protein
BPHY_RS23145	-1,651	0,006233		Bphy_4605	hypothetical protein
BPHY_RS23240	-7,264	7,65E-05	phnC	Bphy_4623	phosphonate ABC transporter ATP-binding protein
BPHY_RS23255	1,734	2,48E-05		Bphy_4626	NAD-glutamate dehydrogenase
BPHY_RS23270	1,581	1,74E-05		Bphy_4629	purine nucleoside permease
BPHY_RS23280	-5,391	0,002475		Bphy_4631	DUF4148 domain-containing protein
BPHY_RS23295	-1,926	0,000962		Bphy_4634	efflux RND transporter periplasmic adaptorsubunit
BPHY_RS23300	-2,337	9,41E-09		Bphy_4635	multidrug efflux RND transporter permeasesubunit
BPHY_RS23305	-1,836	0,003929		Bphy_4636	efflux transporter outer membrane subunit
BPHY_RS23325	1,274	0,000323		Bphy_4641	2OG-Fe(II) oxygenase
BPHY_RS23335	1,261	0,00071		Bphy_4643	helix-turn-helix transcriptional regulator
BPHY_RS23340	2,678	0,00085		Bphy_4644	hypothetical protein
BPHY_RS23370	3,231	1,05E-14		Bphy_4650	Hsp20/alpha crystallin family protein
BPHY_RS23410	-1,112	0,009206		Bphy_4658	sugar ABC transporter ATP-binding protein
BPHY_RS23440	-3,168	4,44E-06		Bphy_4664	MFS transporter
BPHY_RS23450	-3,760	0,001049		Bphy_4666	dihydrodipicolinate synthase family protein
BPHY_RS23455	-4,112	0,005482		Bphy_4667	4-hydroxyproline epimerase
BPHY_RS23505	2,840	3,56E-17		Bphy_4677	hypothetical protein
BPHY_RS23550	1,101	0,005655		Bphy_4687	sensor histidine kinase
BPHY_RS23560	1,096	0,001804		Bphy_4689	OFA family MFS transporter
BPHY_RS23605	-1,890	0,009012	oxlT	Bphy_4698	oxalate/formate MFS antiporter
BPHY_RS23630	1,557	2,74E-05		Bphy_4704	DUF2252 domain-containing protein
BPHY_RS23635	1,878	0,000425		Bphy_4705	GlsB/YeaQ/YmgE family stress response membraneprotein
BPHY_RS23640	2,164	4,48E-06		Bphy_4706	polysaccharide deacetylase family protein
BPHY_RS23650	-2,014	5,16E-06		Bphy_4708	D-amino acid dehydrogenase
BPHY_RS23660	1,796	0,002956		Bphy_4710	SDR family oxidoreductase
BPHY_RS23685	1,382	0,000977	gndA	Bphy_4715	NADP-dependent phosphogluconate dehydrogenase
BPHY_RS23690	1,696	0,000181		Bphy_4716	DUF4148 domain-containing protein
BPHY_RS23695	2,468	7,23E-12		Bphy_4717	DUF2968 domain-containing protein
BPHY_RS23730	1,401	0,000193		Bphy_4725	PIG-L family deacetylase
BPHY_RS23735	1,641	6,83E-06		Bphy_4726	nucleotidyltransferase family protein
BPHY_RS23755	-1,596	0,002396		Bphy_4730	aspartate carbamoyltransferase

BPHY_RS23765	-3,570	7,93E-05		Bphy_4732	M23 family metallopeptidase
BPHY_RS23780	-1,696	0,002964		Bphy_4735	bifunctional diguanylatecyclase/phosphodiesterase
BPHY_RS23795	-1,787	0,00065		Bphy_4738	recombination-associated protein RdgC
BPHY_RS23805	-3,403	1,93E-05		Bphy_4740	glutamate/aspartate ABC transportersubstrate-binding protein
BPHY_RS23810	-6,669	0,002247		Bphy_4741	hypothetical protein
BPHY_RS23845	-2,867	1,83E-05		Bphy_4748	ABC transporter ATP-binding protein/permease
BPHY_RS23865	-3,054	0,009858		Bphy_4752	sigma-54 dependent transcriptional regulator
BPHY_RS23870	-1,895	0,006869		Bphy_4753	glutathione-dependent formaldehydedehydrogenase
BPHY_RS23895	-2,985	1,5E-05		Bphy_4758	Na/Pi cotransporter family protein
BPHY_RS23955	-6,458	0,004176		Bphy_4769	hypothetical protein
BPHY_RS24000	-6,195	0,008807		Bphy_4779	DUF883 family protein
BPHY_RS24020	1,024	0,001112		Bphy_4783	AAA family ATPase
BPHY_RS24055	-5,488	2,06E-05	coxB	Bphy_4792	cytochrome c oxidase subunit II
BPHY_RS24075	-1,959	0,003172		Bphy_4796	cytochrome c
BPHY_RS24080	-3,921	0,005979		Bphy_4797	ATP-dependent DNA helicase
BPHY_RS24135	-1,566	0,000474		Bphy_4808	alkene reductase
BPHY_RS24175	1,091	0,006175		Bphy_4817	i familyantitoxin domain-containing protein
BPHY_RS24215	-3,268	0,000117		Bphy_4826	crityl-CoA lyase
BPHY_RS24235	-2,256	0,002129		Bphy_4830	electron transfer flavoprotein subunit beta/FixAfamily protein
BPHY_RS24245	-1,481	0,00889		Bphy_4832	FAD-dependent oxidoreductase
BPHY_RS24325	-3,613	0,00925		Bphy_4849	tartrate dehydrogenase
BPHY_RS24380	-6,173	0,009242	istB	Bphy_4858	IS21-like element helper ATPase IstB
BPHY_RS24410	-4,126	0,000623		Bphy_4864	DUF1302 domain-containing protein
BPHY_RS24415	-2,095	0,0025		Bphy_4865	MMPL family transporter
BPHY_RS24425	-2,037	0,000506		Bphy_4867	choline dehydrogenase
BPHY_RS24470	-2,117	0,000306		Bphy_4876	carboxylesterase family protein
BPHY_RS24490	-4,993	4,25E-05		Bphy_4880	PDR/VanB family oxidoreductase
BPHY_RS24495	-2,201	0,000304		Bphy_4881	Rieske 2Fe-2S domain-containing protein
BPHY_RS24560	-2,123	0,005754		Bphy_4895	helix-turn-helix domain-containing protein
BPHY_RS24670	1,589	0,000237		Bphy_4919	IS66 family transposase
BPHY_RS24700	-6,219	0,002795		Bphy_4925	gamma carbonic anhydrase family protein
BPHY_RS24715	1,075	0,008733		Bphy_4928	LysR family transcriptional regulator
BPHY_RS24835	1,528	0,000299		Bphy_4956	IS110 family transposase
BPHY_RS24900	1,308	0,001849		Bphy_4972	ATP-binding protein
BPHY_RS24960	3,052	2,33E-07		Bphy_4984	BON domain-containing protein
BPHY_RS24990	1,312	0,001452		Bphy_4990	MFS transporter
BPHY_RS25005	1,447	0,000279		Bphy_4993	aspartate/glutamate racemase family protein
BPHY_RS25040	-3,734	1,43E-05		Bphy_5000	MFS transporter
BPHY_RS25145	1,528	0,001308		Bphy_5021	pentapeptide MXKDX repeat protein
BPHY_RS25150	1,281	0,009884		Bphy_5022	class I SAM-dependent methyltransferase
BPHY_RS25175	-2,650	0,00036		Bphy_5027	sensor domain-containing diguanylate cyclase
BPHY_RS25180	-3,496	0,00014		Bphy_5028	EAL domain-containing protein
BPHY_RS25190	-2,505	0,004834		Bphy_5030	TonB-dependent receptor

BPHY_RS25205	-2,942	0,000594		Bphy_5033	cytochrome c biogenesis protein DipZ
BPHY_RS25215	1,529	1,81E-05		Bphy_5035	alpha/beta hydrolase
BPHY_RS25230	-1,692	0,000139	ilvD	Bphy_5037	dihydroxy-acid dehydratase
BPHY_RS25260	-3,018	0,000323		Bphy_5042	ATP-binding protein
BPHY_RS25315	1,760	0,000269		Bphy_5053	DUF1993 domain-containing protein
BPHY_RS25320	2,016	2,41E-08		Bphy_5054	S53 family peptidase
BPHY_RS25325	3,093	7,46E-19		Bphy_5055	peptidase S1
BPHY_RS25330	1,026	0,004395		Bphy_5056	hypothetical protein
BPHY_RS25460	3,209	9,19E-15		Bphy_5077	acetyl-CoA hydrolase/transferase family protein
BPHY_RS25535	1,548	0,000112		Bphy_5092	hypothetical protein
BPHY_RS25550	2,406	0,000103	poxB	Bphy_5095	ubiquinone-dependent pyruvate dehydrogenase
BPHY_RS25615	1,976	0,000671		Bphy_5109	amino acid ABC transporter permease
BPHY_RS25620	3,255	5,41E-19		Bphy_5110	transporter substrate-binding domain-containingprotein
BPHY_RS25625	1,785	6,81E-05		Bphy_5111	isopenicillin N synthase family oxygenase
BPHY_RS25635	1,271	0,001585		Bphy_5113	GMC family oxidoreductase N-terminaldomain-containing protein
BPHY_RS25660	1,088	0,002348	cydB	Bphy_5119	cytochrome d ubiquinol oxidase subunit II
BPHY_RS25670	-1,215	0,00548		Bphy_5121	NCS2 family permease
BPHY_RS25710	1,772	2,15E-05	mmsB	Bphy_5129	3-hydroxyisobutyrate dehydrogenase
BPHY_RS25725	2,165	0,000801		Bphy_5132	2Fe-2S iron-sulfur cluster bindingdomain-containing protein
BPHY_RS25730	2,153	0,000274		Bphy_5133	xanthine dehydrogenase family protein subunit M
BPHY_RS25745	-1,505	0,005171		Bphy_5136	siderophore-interacting protein
BPHY_RS25835	-6,923	0,000147		Bphy_5154	MFS transporter
BPHY_RS25890	1,637	0,000313		Bphy_5165	SDR family oxidoreductase
BPHY_RS25895	-2,017	0,000509		Bphy_5166	LysR family transcriptional regulator
BPHY_RS25920	1,470	0,000983		Bphy_5171	response regulator
BPHY_RS25995	-2,258	0,005141		Bphy_5188	DMT family transporter
BPHY_RS26045	1,522	8,6E-06		Bphy_5198	hypothetical protein
BPHY_RS26070	2,044	6,98E-06		Bphy_5204	hypothetical protein
BPHY_RS26080	2,081	8,08E-07		Bphy_5206	amylo-alpha-1,6-glucosidase
BPHY_RS26085	1,819	0,000855		Bphy_5207	YukJ family protein
BPHY_RS26090	2,005	5,45E-06		Bphy_5208	Ku protein
BPHY_RS26100	2,436	1,63E-10		Bphy_5210	hypothetical protein
BPHY_RS26120	2,274	3,69E-05		Bphy_5214	hypothetical protein
BPHY_RS26160	1,606	4,48E-06		Bphy_5222	DUF4148 domain-containing protein
BPHY_RS26165	2,985	1,73E-14		Bphy_5223	OsmC family protein
BPHY_RS26170	1,741	6,47E-05		Bphy_5224	isocitrate lyase/phosphoenolpyruvate mutasefamily protein
BPHY_RS26285	-2,615	0,003713		Bphy_5247	carbamoyltransferase
BPHY_RS26350	2,755	2,37E-05		Bphy_5261	Hsp20/alpha crystallin family protein
BPHY_RS26355	3,182	2,22E-15		Bphy_5262	Hsp20/alpha crystallin family protein
BPHY_RS26400	1,699	0,009567		Bphy_5272	YodC family protein
BPHY_RS26405	2,143	1,23E-06		Bphy_5273	LysE family translocator
BPHY_RS26410	1,863	1,32E-06		Bphy_5274	epoxide hydrolase
BPHY_RS26415	1,968	0,001266		Bphy_5275	superinfection immunity protein

BPHY_RS26435	1,449	9,57E-06	pdeR	Bphy_5279	cyclic di-GMP phosphodiesterase
BPHY_RS26440	2,504	8,54E-15	treA	Bphy_5280	alpha,alpha-trehalase TreA
BPHY_RS26445	1,805	2,12E-05		Bphy_5281	DEAD/DEAH box helicase
BPHY_RS26470	2,067	3,8E-07		Bphy_5288	hypothetical protein
BPHY_RS26480	1,457	1,29E-06		Bphy_5290	4/polysaccharide deacetylase family protein
BPHY_RS26485	1,510	6,16E-06		Bphy_5291	cation:proton antiporter
BPHY_RS26500	1,987	3,29E-06		Bphy_5294	HAD family hydrolase
BPHY_RS26525	1,173	0,003828		Bphy_5299	DUF4142 domain-containing protein
BPHY_RS26545	3,797	1,52E-11		Bphy_5303	BON domain-containing protein
BPHY_RS26595	1,305	0,000306		Bphy_5314	sigma-54 dependent transcriptional regulator
BPHY_RS26640	-2,369	0,003707		Bphy_5323	AAA family ATPase
BPHY_RS26655	-5,459	0,003278		Bphy_5326	type II secretion system F family protein
BPHY_RS26725	1,462	0,001107		Bphy_5340	amidohydrolase family protein
BPHY_RS26730	1,374	0,002453		Bphy_5341	hypothetical protein
BPHY_RS26740	1,202	0,005338		Bphy_5343	alpha/beta fold hydrolase
BPHY_RS26815	1,874	0,000159		Bphy_5358	hypothetical protein
BPHY_RS26820	1,213	0,002703		Bphy_5359	acid phosphatase
BPHY_RS26880	-6,785	4,77E-34		Bphy_5373	TonB-dependent siderophore receptor
BPHY_RS26885	-2,844	5,78E-08		Bphy_5374	Fe2+-dependent dioxygenase
BPHY_RS26895	5,008	6,39E-43		Bphy_5376	iron-sulfur cluster-binding protein
BPHY_RS26900	5,246	1,07E-37		Bphy_5377	lactate utilization protein C
BPHY_RS26905	4,270	3,64E-28		Bphy_5378	(Fe-S)-binding protein
BPHY_RS27015	1,919	0,000257		Bphy_5400	antibiotic biosynthesis monooxygenase
BPHY_RS27025	1,411	0,001651		Bphy_5402	NAD(P)-dependent oxidoreductase
BPHY_RS27055	-4,886	0,002517		Bphy_5408	UdgX family uracil-DNA binding protein
BPHY_RS27085	1,950	0,004873		Bphy_5413	hypothetical protein
BPHY_RS27195	1,102	0,002761		Bphy_5438	ABC transporter ATP-binding protein
BPHY_RS27225	1,174	0,004207		Bphy_5444	aspartate/tyrosine/aromatic aminotransferase
BPHY_RS27340	1,419	0,000218		Bphy_5467	amidase
BPHY_RS27375	1,260	1,48E-05		Bphy_5473	Dyp-type peroxidase
BPHY_RS27380	2,435	2,2E-05	fae	Bphy_5474	formaldehyde-activating enzyme
BPHY_RS27395	1,255	0,00281		Bphy_5477	membrane protein
BPHY_RS27430	1,961	1,12E-06		Bphy_5484	catalase family peroxidase
BPHY_RS27450	-6,492	0,003861		Bphy_5488	hypothetical protein
BPHY_RS27485	1,526	4,43E-05		Bphy_5495	ATP-dependent Clp protease ATP-binding subunit
BPHY_RS27495	1,388	0,000261		Bphy_5497	phosphoketolase family protein
BPHY_RS27500	-7,972	6,46E-08		Bphy_5498	DUF421 domain-containing protein
BPHY_RS27520	2,077	0,000603		Bphy_5502	response regulator
BPHY_RS27545	4,507	9,8E-26		Bphy_5507	PqiC family protein
BPHY_RS27550	5,412	1,95E-34	ppk2	Bphy_5508	polyphosphate kinase 2
BPHY_RS27555	5,275	1,05E-28		Bphy_5509	RNA-binding protein
BPHY_RS27565	2,118	4,9E-08		Bphy_5510	hypothetical protein
BPHY_RS27575	3,302	2,16E-12		Bphy_5512	poly(3-hydroxybutyrate) depolymerase

BPHY_RS27580	1,579	0,009835		Bphy_5513	hypothetical protein
BPHY_RS27730	-1,696	0,002703		Bphy_5543	indolepyruvate ferredoxin oxidoreductase familyprotein
BPHY_RS27735	-2,207	0,001952		Bphy_5544	LysR family transcriptional regulator
BPHY_RS27775	-2,119	0,000283		Bphy_5551	hypothetical protein
BPHY_RS27785	4,763	9,83E-30		Bphy_5552	universal stress protein
BPHY_RS27800	3,205	1,43E-06		Bphy_5555	arylsulfatase
BPHY_RS27805	1,973	0,001148		Bphy_5556	haloacid dehalogenase-like hydrolase
BPHY_RS27810	2,782	4,02E-05		Bphy_5557	anaerobic sulfatase maturase
BPHY_RS27845	3,640	1,93E-25		Bphy_5562	ıbrane-boundPQQ-dependent dehydrogenase
BPHY_RS27940	-1,302	0,00733		Bphy_5579	SpoIIE family protein phosphatase
BPHY_RS27975	-1,388	0,002643	sdhD	Bphy_5587	succinate dehydrogenase, hydrophobic membraneanchor protein
BPHY_RS27980	-1,479	0,000541		Bphy_5588	succinate dehydrogenase flavoprotein subunit
BPHY_RS27985	-2,062	0,005984		Bphy_5589	succinate dehydrogenase iron-sulfur subunit
BPHY_RS28000	-2,134	0,000265		Bphy_5592	methyl-accepting chemotaxis protein
BPHY_RS28030	1,802	0,000147		Bphy_5598	GntR family transcriptional regulator
BPHY_RS28035	2,385	1,37E-09		Bphy_5599	L-fuconate dehydratase
BPHY_RS28040	1,962	0,000402		Bphy_5600	haloacid dehalogenase type II
BPHY_RS28060	-2,521	8,23E-06		Bphy_5604	hypothetical protein
BPHY_RS28065	-1,328	0,008384		Bphy_5606	sensor histidine kinase
BPHY_RS28125	-4,226	0,006761		Bphy_5616	chemotaxis protein
BPHY_RS28245	2,924	3,58E-10		Bphy_5640	BON domain-containing protein
BPHY_RS28250	5,180	1,52E-34	glcF	Bphy_5641	glycolate oxidase subunit GlcF
BPHY_RS28255	4,055	2,63E-13	glcE	Bphy_5642	glycolate oxidase subunit GlcE
BPHY_RS28260	4,213	1,84E-25	glcD	Bphy_5643	glycolate oxidase subunit GlcD
BPHY_RS28270	-2,009	0,009132		Bphy_5645	alpha/beta hydrolase
BPHY_RS28280	-2,821	6,77E-08		Bphy_5647	hypothetical protein
BPHY_RS28285	-2,016	0,009788		Bphy_5648	transposase
BPHY_RS28295	1,760	6,05E-06		Bphy_5650	3-hydroxybutyryl-CoA dehydrogenase
BPHY_RS28300	1,281	0,000783		Bphy_5651	GNAT family N-acetyltransferase
BPHY_RS28315	-2,102	0,002929		Bphy_5654	MFS transporter
BPHY_RS28325	1,464	0,004611		Bphy_5656	LysR family transcriptional regulator
BPHY_RS28330	-3,408	0,003108		Bphy_5658	MFS transporter
BPHY_RS28335	-2,182	0,002779	nirB	Bphy_5659	nitrite reductase large subunit NirB
BPHY_RS28345	-2,250	0,000778		Bphy_5661	molybdopterin-dependent oxidoreductase
BPHY_RS28355	1,473	0,004213		Bphy_5663	hypothetical protein
BPHY_RS28410	2,016	1,04E-05			hypothetical protein
BPHY_RS28430	2,458	8,01E-11		Bphy_5676	hypothetical protein
BPHY_RS28435	1,760	0,00849		Bphy_5678	hypothetical protein
BPHY_RS28440	1,589	2,54E-06		Bphy_5679	PAS domain S-box protein
BPHY_RS28460	1,851	0,000899		Bphy_5683	organic hydroperoxide resistance protein
BPHY_RS28465	2,336	3,31E-10		Bphy_5684	4-oxalocrotonate tautomerase family protein
BPHY_RS28490	1,516	0,000311		Bphy_5689	VOC family protein
BPHY_RS28590	-1,671	0,00094		Bphy_5711	winged helix-turn-helix domain-containingprotein

BPHY_RS28600	-2,118	0,003371		Bphy_5713	FUSC family protein
BPHY_RS28620	-3,612	0,004348		Bphy_5718	alpha/beta hydrolase
BPHY_RS28630	-1,566	0,000963		Bphy_5720	hydrolase
BPHY_RS28655	-4,060	0,000122		Bphy_5725	DUF1275 domain-containing protein
BPHY_RS28660	-1,857	0,005036		Bphy_5726	hypothetical protein
BPHY_RS28665	-2,632	0,000107		Bphy_5727	alginate export family protein
BPHY_RS28690	-1,280	0,006518		Bphy_5732	hemagglutinin repeat-containing protein
BPHY_RS28785	-5,826	0,009338		Bphy_5752	hypothetical protein
BPHY_RS28870	-1,431	0,00042		Bphy_5769	AAA family ATPase
BPHY_RS28890	-1,622	0,002483		Bphy_5773	glutaminase
BPHY_RS28920	-2,625	0,002266		Bphy_5779	tetratricopeptide repeat protein
BPHY_RS28925	-3,130	0,000141		Bphy_5780	phosphatase PAP2 family protein
BPHY_RS28940	1,512	5,75E-06		Bphy_5783	M14 family metallopeptidase
BPHY_RS28955	-5,810	0,008927		Bphy_5786	DUF3331 domain-containing protein
BPHY_RS29050	-2,355	0,001043		Bphy_5806	AMP-binding protein
BPHY_RS29065	1,722	3,35E-08		Bphy_5809	PAS domain S-box protein
BPHY_RS29070	5,381	1,25E-46		Bphy_5810	DUF3564 domain-containing protein
BPHY_RS29075	2,438	1,7E-09	fnr	Bphy_5811	fumarate/nitrate reduction transcriptionalregulator Fnr
BPHY_RS29080	1,835	4,19E-07		Bphy_5812	response regulator
BPHY_RS29090	3,361	1,72E-10		Bphy_5814	universal stress protein
BPHY_RS29095	4,420	1,76E-25		Bphy_5815	universal stress protein
BPHY_RS29100	5,034	9,29E-44		Bphy_5816	zinc-dependent alcohol dehydrogenase familyprotein
BPHY_RS29105	4,583	1,07E-31		Bphy_5817	flavodoxin
BPHY_RS29110	5,209	3,3E-20		Bphy_5818	universal stress protein
BPHY_RS29115	3,640	2,56E-22		Bphy_5819	nitroreductase family protein
BPHY_RS29120	3,044	2,2E-07		Bphy_5820	universal stress protein
BPHY_RS29125	5,029	4,03E-21		Bphy_5821	CBS domain-containing protein
BPHY_RS29130	3,579	2,5E-19		Bphy_5822	glucose 1-dehydrogenase
BPHY_RS29135	1,165	0,001479		Bphy_5823	PLP-dependent aminotransferase family protein
BPHY_RS29180	-1,500	0,001312		Bphy_5832	DUF2322 family protein
BPHY_RS29225	3,542	5,44E-14		Bphy_5841	aldolase
BPHY_RS29230	3,350	2,89E-12		Bphy_5842	haloacid dehalogenase type II
BPHY_RS29250	1,322	0,006		Bphy_5848	hypothetical protein
BPHY_RS29255	1,490	3,7E-05		Bphy_5849	MFS transporter
BPHY_RS29275	1,371	0,005795		Bphy_5853	CHAD domain-containing protein
BPHY_RS29285	-3,439	7,36E-22	acnB	Bphy_5855	bifunctional aconitate hydratase2/2-methylisocitrate dehydratase
BPHY_RS29340	3,321	2,67E-14		Bphy_5869	universal stress protein
BPHY_RS29345	4,186	2,33E-35	adhP	Bphy_5870	alcohol dehydrogenase AdhP
BPHY_RS29350	2,126	0,000163		Bphy_5871	hypothetical protein
BPHY_RS29445	1,334	0,000102		Bphy_5889	C transportersubstrate-binding protein
BPHY_RS29475	1,772	0,004811		Bphy_5897	ribonuclease
BPHY_RS29490	-2,357	0,000607		Bphy_5899	GGDEF domain-containing phosphodiesterase
BPHY_RS29545	-6,109	0,002841		Bphy_5910	hypothetical protein

BPHY_RS29560	-1,820	0,008977	purU	Bphy_5913	formyltetrahydrofolate deformylase
BPHY_RS29565	-2,559	0,006522		Bphy_5914	FAD-dependent oxidoreductase
BPHY_RS29575	-1,769	0,005916		Bphy_5916	FAD-dependent oxidoreductase
BPHY_RS29615	-3,434	0,008502		Bphy_5924	CoA ester lyase
BPHY_RS29665	-3,504	0,003705		Bphy_5934	formylmethanofuran dehydrogenase subunit A
BPHY_RS29670	-3,908	0,009658		Bphy_5935	formylmethanofuran dehydrogenase
BPHY_RS29745	-4,998	0,000435		Bphy_5950	LysR family transcriptional regulator
BPHY_RS29790	-5,142	1,12E-05		Bphy_5959	4Fe-4S dicluster domain-containing protein
BPHY_RS29795	-2,599	0,003623		Bphy_5960	FAD-dependent oxidoreductase
BPHY_RS29830	1,633	3,22E-07		Bphy_5967	histidine kinase
BPHY_RS29845	-2,573	0,000951		Bphy_5970	APC family permease
BPHY_RS29865	1,246	0,007037		Bphy_5974	AAA family ATPase
BPHY_RS29885	-3,020	2,21E-07	tssB	Bphy_5978	type VI secretion system contractile sheathsmall subunit
BPHY_RS29890	-2,512	7,09E-08	tssC	Bphy_5979	type VI secretion system contractile sheathlarge subunit
BPHY_RS29895	-3,181	5,93E-09		Bphy_5980	type VI secretion system tube protein Hcp
BPHY_RS29900	-2,648	0,000127	tssE	Bphy_5981	type VI secretion system baseplate subunit TssE
BPHY_RS29905	-2,632	9,37E-08	tssF	Bphy_5982	type VI secretion system baseplate subunit TssF
BPHY_RS29910	-2,474	1,11E-06	tssG	Bphy_5983	type VI secretion system baseplate subunit TssG
BPHY_RS29915	-2,491	1,15E-09	tssH	Bphy_5984	type VI secretion system ATPase TssH
BPHY_RS29920	-2,090	9,57E-06	tssI	Bphy_5985	type VI secretion system tip protein VgrG
BPHY_RS29925	-2,323	0,007098		Bphy_5986	toxin-antitoxin system YwqK family antitoxin
BPHY_RS29935	-4,137	0,003283		Bphy_5988	hypothetical protein
BPHY_RS29940	-2,410	6,62E-06		Bphy_5989	ABC transporter substrate-binding protein
BPHY_RS29945	-2,764	5,12E-08		Bphy_5990	cation:dicarboxylase symporter familytransporter
BPHY_RS29950	-1,889	0,000362		Bphy_5991	amino acid racemase
BPHY_RS29955	-2,356	2,37E-06		Bphy_5992	cation:dicarboxylase symporter familytransporter
BPHY_RS29965	-1,593	0,001026	tssK	Bphy_5994	type VI secretion system baseplate subunit TssK
BPHY_RS29970	-4,118	0,00081		Bphy_5995	DotU family type IV/VI secretion system protein
BPHY_RS29975	-1,531	0,0003		Bphy_5996	type VI secretion system protein
BPHY_RS29990	1,721	0,000117		Bphy_5999	helix-turn-helix transcriptional regulator
BPHY_RS30000	2,172	9,36E-05		Bphy_6003	hypothetical protein
BPHY_RS30010	3,179	7,56E-06		Bphy_6005	DUF2933 domain-containing protein
BPHY_RS30015	2,984	1,64E-09		Bphy_6006	isoprenylcysteine carboxylmethyltransferasefamily protein
BPHY_RS30020	2,928	4,38E-10	ftsH	Bphy_6007	ATP-dependent zinc metalloprotease FtsH
BPHY_RS30030	1,448	0,000475		Bphy_6008	heavy metal translocating P-type ATPase
BPHY_RS30125	-6,729	0,000103		Bphy_6027	diguanylate cyclase
BPHY_RS30135	-4,834	0,000816		Bphy_6029	SMP-30/gluconolactonase/LRE family protein
BPHY_RS30140	-3,493	0,004923		Bphy_6030	MFS transporter
BPHY_RS30150	-3,955	0,005022		Bphy_6032	amidohydrolase family protein
BPHY_RS30155	-3,242	0,000426		Bphy_6033	MFS transporter
BPHY_RS30160	-3,011	0,005946		Bphy_6034	MFS transporter
BPHY_RS30260	2,753	0,00039		Bphy_6054	CBS domain-containing protein
BPHY_RS30265	2,987	1,38E-05		Bphy_6055	flavodoxin

BPHY_RS30270	1,127	0,00758		Bphy_6060	hypothetical protein
BPHY_RS30290	1,036	0,002748	hyfB	Bphy_6065	hydrogenase 4 subunit B
BPHY_RS30295	1,309	0,001174		Bphy_6066	NADH-quinone oxidoreductase subunit H
BPHY_RS30310	1,480	7,09E-06		Bphy_6069	NADH-quinone oxidoreductase subunit C
BPHY_RS30315	1,326	0,000366		Bphy_6070	NADH-quinone oxidoreductase subunit B familyprotein
BPHY_RS30350	-2,114	0,002098	tauA	Bphy_6080	taurine ABC transporter substrate-bindingprotein
BPHY_RS30355	-3,821	0,005304		Bphy_6081	ATP-binding cassette domain-containing protein
BPHY_RS30420	-2,937	6,04E-05		Bphy_6094	FAD-dependent oxidoreductase
BPHY_RS30445	-6,645	0,000459		Bphy_6099	ABC transporter substrate-binding protein
BPHY_RS30460	-1,748	5,58E-05		Bphy_6102	FAD-dependent oxidoreductase
BPHY_RS30475	-3,822	0,005098		Bphy_6105	MFS transporter
BPHY_RS30500	1,175	0,004031	tssK	Bphy_6110	type VI secretion system baseplate subunit TssK
BPHY_RS30505	2,109	4,31E-05	tssJ	Bphy_6111	type VI secretion system lipoprotein TssJ
BPHY_RS30510	1,515	0,001161		Bphy_6112	Hcp family type VI secretion system effector
BPHY_RS30515	2,039	3,4E-10	tssC	Bphy_6113	type VI secretion system contractile sheathlarge subunit
BPHY_RS30520	2,179	5,52E-09	tssB	Bphy_6114	type VI secretion system contractile sheathsmall subunit
BPHY_RS30525	1,527	0,000312	tssH	Bphy_6115	type VI secretion system ATPase TssH
BPHY_RS30535	1,112	0,006326		Bphy_6117	virulence protein SciE type
BPHY_RS30540	1,675	0,000235	tssE	Bphy_6118	type VI secretion system baseplate subunit TssE
BPHY_RS30545	1,493	1,46E-05	tssF	Bphy_6119	type VI secretion system baseplate subunit TssF
BPHY_RS30550	1,639	6,63E-06	tssG	Bphy_6120	type VI secretion system baseplate subunit TssG
BPHY_RS30565	1,961	1,13E-09	tssI	Bphy_6124	type VI secretion system tip protein VgrG
BPHY_RS30595	1,733	7,23E-06		Bphy_6134	IS5-like element ISBph2 family transposase
BPHY_RS30660	1,520	0,002278		Bphy_6148	helix-turn-helix domain-containing protein
BPHY_RS30705	-6,683	0,002299		Bphy_6159	2-dehydropantoate 2-reductase
BPHY_RS30830	1,969	0,001197	narH	Bphy_6185	nitrate reductase subunit beta
BPHY_RS30835	2,168	0,000181	narJ	Bphy_6186	nitrate reductase molybdenum cofactor assemblychaperone
BPHY_RS30855	1,979	6,51E-05		Bphy_6190	hemerythrin domain-containing protein
BPHY_RS30860	2,842	8,93E-20		Bphy_6191	transducer N-terminal domain-containing protein
BPHY_RS30865	2,521	1,15E-11		Bphy_6192	response regulator
BPHY_RS30870	2,521	2,65E-07		Bphy_6193	2Fe-2S iron-sulfur cluster bindingdomain-containing protein
BPHY_RS30875	3,974	1,45E-30	hcp	Bphy_6194	hydroxylamine reductase
BPHY_RS30935	1,270	0,00131		Bphy_6209	AAA family ATPase
BPHY_RS30950	-2,227	0,005887		Bphy_6212	dimethyl sulfoxide reductase anchor subunit
BPHY_RS30995	-4,062	0,000467		Bphy_6221	choline dehydrogenase
BPHY_RS31035	2,072	2,05E-06		Bphy_6230	GNAT family N-acetyltransferase
BPHY_RS31075	2,659	1,38E-17		Bphy_6238	TIGR01244 family phosphatase
BPHY_RS31080	1,626	0,000132		Bphy_6239	MBL fold metallo-hydrolase
BPHY_RS31105	4,544	1,43E-43		Bphy_6244	Spy/CpxP family protein refolding chaperone
BPHY_RS31110	2,731	6,77E-09		Bphy_6245	sulfurtransferase
BPHY_RS31115	3,329	2,43E-23		Bphy_6246	hypothetical protein
BPHY_RS31120	3,673	1,03E-20		Bphy_6247	universal stress protein
BPHY_RS31130	2,419	0,000267		Bphy_6249	hypothetical protein

BPHY_RS31140	1,355	0,000907	Bphy_6251	ABC transporter permease
BPHY_RS31150	2,317	6,31E-09	Bphy_6254	substrate-binding protein
BPHY_RS31255	-2,020	0,007537	Bphy_6277	FAD-dependent monooxygenase
BPHY_RS31290	1,210	0,003706	Bphy_6284	M24 family metallopeptidase
BPHY_RS31315	1,903	7,78E-05	Bphy_6291	porin
BPHY_RS31345	1,570	0,000206	Bphy_6298	PQQ-dependent sugar dehydrogenase
BPHY_RS31375	4,533	1,17E-36	Bphy_6305	pyridoxalphosphate-dependent enzyme
BPHY_RS31380	5,910	2,47E-72	Bphy_6306	fatty acyl-AMP ligase
BPHY_RS31385	6,278	5,01E-48	Bphy_6307	cytochrome P450
BPHY_RS31390	4,267	8,23E-20	Bphy_6308	isoprenylcysteine carboxylmethyltransferasefamily protein
BPHY_RS31395	4,423	1,91E-09	Bphy_6309	glutathione S-transferase family protein
BPHY_RS31400	4,436	5,67E-21	Bphy_6310	membrane protein
BPHY_RS31405	3,696	1,59E-21	Bphy_6311	DUF2156 domain-containing protein
BPHY_RS31410	4,456	1,9E-51	Bphy_6312	FAD-binding oxidoreductase
BPHY_RS31415	1,293	0,000441	Bphy_6313	acyl-CoA/acyl-ACP dehydrogenase
BPHY_RS31445	2,038	8,77E-09	Bphy_6320	excinuclease ABC subunit UvrA
BPHY_RS31450	1,993	2,46E-05	Bphy_6321	Uma2 family endonuclease
BPHY_RS31475	-4,920	0,009442	Bphy_6326	hypothetical protein
BPHY_RS31495	1,997	3,45E-09	Bphy_6330	helix-turn-helix domain-containing protein
BPHY_RS31500	5,000	6,87E-19	Bphy_6331	hypothetical protein
BPHY_RS31515	1,643	0,001609	Bphy_6334	cytochrome b/b6 domain-containing protein
BPHY_RS31600	3,505	3,37E-08	Bphy_6350	DUF3313 domain-containing protein
BPHY_RS31620	1,848	0,004499	Bphy_6354	DUF4148 domain-containing protein
BPHY_RS31625	-5,744	6,82E-05	Bphy_6355	porin
BPHY_RS31645	1,131	0,007642	Bphy_6359	DUF3047 domain-containing protein
BPHY_RS31740	-7,043	9,04E-05	Bphy_6378	hypothetical protein
BPHY_RS31765	1,508	0,003423	Bphy_6383	FAD-binding monooxygenase
BPHY_RS31845	1,176	0,000409	Bphy_6399	PAS domain-containing protein
BPHY_RS31850	1,378	0,002569	Bphy_6400	hypothetical protein
BPHY_RS31860	-2,654	0,002736	Bphy_6402	hypothetical protein
BPHY_RS31940	-5,137	0,001787	Bphy_6416	enoyl-CoA hydratase/isomerase family protein
BPHY_RS31975	-2,154	0,000851	Bphy_6422	molybdopterin-dependent oxidoreductase
BPHY_RS31980	-2,303	0,000772	Bphy_6423	cytochrome b/b6 domain-containing protein
BPHY_RS31990	2,135	1,12E-08	Bphy_6425	elongation factor G
BPHY_RS32000	-1,735	0,005743	Bphy_6427	OmpA family protein
BPHY_RS32005	-1,660	2,8E-05	Bphy_6428	DUF802 domain-containing protein
BPHY_RS32020	-1,855	0,002212	Bphy_6431	DMT family transporter
BPHY_RS32055	2,918	5,5E-08	Bphy_6438	YMGG-like glycine zipper-containing protein
BPHY_RS32085	1,643	0,002188	Bphy_6444	hypothetical protein
BPHY_RS32105	2,969	1,67E-05	Bphy_6448	hypothetical protein
BPHY_RS32110	3,043	4,26E-05	Bphy_6449	hypothetical protein
BPHY_RS32145	2,245	0,000112	Bphy_6456	AI-2E family transporter
BPHY_RS32150	2,573	5,6E-10	Bphy_6457	hypothetical protein

BPHY_RS32195	1,391	0,00579		Bphy_6466	PQQ-binding-like beta-propeller repeat protein
BPHY_RS32230	-2,022	0,000275		Bphy_6473	alkylphosphonate utilization protein
BPHY_RS32245	1,434	0,000164		Bphy_6476	substrate-binding domain-containing protein
BPHY_RS32265	2,159	0,000288		Bphy_6480	4a-hydroxytetrahydrobiopterin dehydratase
BPHY_RS32280	1,439	0,00282		Bphy_6483	ATP-grasp domain-containing protein
BPHY_RS32355	1,839	1,68E-07		Bphy_6498	DUF447 family protein
BPHY_RS32360	1,663	0,000212		Bphy_6499	(5-formylfuran-3-yl)methyl phosphate synthase
BPHY_RS32415	2,340	7,29E-13	pqqC	Bphy_6510	pyrroloquinoline-quinone synthase PqqC
BPHY_RS32420	2,146	9,94E-05	pqqD	Bphy_6511	pyrroloquinoline quinone biosynthesis peptidechaperone PqqD
BPHY_RS32425	2,063	6,05E-09	pqqE	Bphy_6512	pyrroloquinoline quinone biosynthesis proteinPqqE
BPHY_RS32440	-1,571	0,008723		Bphy_6515	beta-propeller fold lactonase family protein
BPHY_RS32560	1,615	0,000191		Bphy_6540	antibiotic biosynthesis monooxygenase
BPHY_RS32565	1,421	1,09E-05		Bphy_6541	enterotoxin
BPHY_RS32570	2,270	1,73E-13		Bphy_6542	beta-galactosidase
BPHY_RS32575	1,750	1,67E-06		Bphy_6543	hypothetical protein
BPHY_RS32580	1,836	0,000463		Bphy_6544	hypothetical protein
BPHY_RS32590	2,832	2,07E-08		Bphy_6546	hypothetical protein
BPHY_RS32615	1,232	0,001767	surE	Bphy_6551	5'/3'-nucleotidase SurE
BPHY_RS32715	1,814	0,00049		Bphy_6571	tartrate dehydrogenase
BPHY_RS32720	1,353	0,001841		Bphy_6572	NAD-dependent succinate-semialdehydedehydrogenase
BPHY_RS32725	1,619	1,25E-05		Bphy_6573	M14 family metallopeptidase
BPHY_RS32735	1,655	1,72E-05		Bphy_6575	ABC transporter substrate-binding protein
BPHY_RS32765	2,013	1,19E-05		Bphy_6581	HAD-IA family hydrolase
BPHY_RS32790	-2,481	0,005041		Bphy_6586	FAD-dependent oxidoreductase
BPHY_RS32800	-2,139	0,005781		Bphy_6588	ABC transporter ATP-binding protein
BPHY_RS32835	1,571	0,000642		Bphy_6595	GNAT family N-acetyltransferase
BPHY_RS32860	2,115	7,37E-05		Bphy_6600	cupin domain-containing protein
BPHY_RS32875	2,340	4,3E-15		Bphy_6603	glucosidase
BPHY_RS32910	1,330	0,000227		Bphy_6610	malate synthase G
BPHY_RS32930	-2,424	0,001077		Bphy_6614	LysR family transcriptional regulator
BPHY_RS32945	2,654	1,34E-09		Bphy_6617	HAD family hydrolase
BPHY_RS32975	1,707	0,000851		Bphy_6622	MBL fold metallo-hydrolase
BPHY_RS32980	3,514	1,99E-26		Bphy_6623	peroxiredoxin
BPHY_RS33080	2,118	2,58E-05		Bphy_6643	response regulator
BPHY_RS33085	2,272	5,56E-09		Bphy_6644	aldo/keto reductase
BPHY_RS33150	1,389	0,007824		Bphy_6656	patatin-like phospholipase family protein
BPHY_RS33180	-3,719	0,000377		Bphy_6664	sugar ABC transporter permease
BPHY_RS33285	1,599	8,02E-08		Bphy_6685	aminotransferase class V-fold PLP-dependentenzyme
BPHY_RS33320	-2,963	3,84E-06	cysS	Bphy_6692	cysteine--tRNA ligase
BPHY_RS33325	-7,512	2,31E-33		Bphy_6693	TonB-dependent receptor
BPHY_RS33395	1,422	0,000271	acnD	Bphy_6708	Fe/S-dependent 2-methylisocitrate dehydrataseAcnD
BPHY_RS33455	-2,301	0,004377		Bphy_6720	hypothetical protein
BPHY_RS33465	-6,639	0,002457		Bphy_6722	hypothetical protein

BPHY_RS33470	-3,014	0,001979		Bphy_6723	porin
BPHY_RS33595	1,741	3,85E-06		Bphy_6748	type 1 glutamine amidotransferasedomain-containing protein
BPHY_RS33675	-4,203	0,004818		Bphy_6766	AbrB family transcriptional regulator
BPHY_RS33865	-3,016	0,006666		Bphy_6805	methyl-accepting chemotaxis protein
BPHY_RS33900	-4,026	0,009688		Bphy_6812	amidohydrolase family protein
BPHY_RS33970	1,680	7,63E-05		Bphy_6827	LysR family transcriptional regulator
BPHY_RS33995	-6,172	0,000219		Bphy_6833	MFS transporter
BPHY_RS34000	1,160	0,0094		Bphy_6834	NAD(P)-dependent oxidoreductase
BPHY_RS34020	-6,962	0,000898		Bphy_6838	porin
BPHY_RS34145	-2,793	0,006307		Bphy_6863	NAD(P)-binding domain-containing protein
BPHY_RS34165	-2,910	0,000354		Bphy_6867	serine hydroxymethyltransferase
BPHY_RS34180	-4,912	8,68E-05		Bphy_6870	glycine betaine/L-proline ABC transporterATP-binding protein
BPHY_RS34190	1,425	1,2E-05		Bphy_6872	hypothetical protein
BPHY_RS34210	-4,658	0,000594		Bphy_6876	MFS transporter
BPHY_RS34235	-1,547	0,004771		Bphy_6882	hypothetical protein
BPHY_RS34275	1,866	0,000823		Bphy_6892	Hsp20/alpha crystallin family protein
BPHY_RS34285	1,696	0,008452		Bphy_6894	PRC-barrel domain-containing protein
BPHY_RS34300	-6,902	0,001093		Bphy_6897	BON domain-containing protein
BPHY_RS34335	1,076	0,004191		Bphy_6904	PLP-dependent aminotransferase family protein
BPHY_RS34425	-3,973	0,005277		Bphy_6925	abortive infection family protein
BPHY_RS34440	-4,017	0,002051		Bphy_6929	hypothetical protein
BPHY_RS34490	2,180	1,28E-11		Bphy_6939	alkaline phosphatase family protein
BPHY_RS34515	1,066	0,001766		Bphy_6946	PAS domain S-box protein
BPHY_RS34630	1,627	9,08E-05		Bphy_6969	TniQ family protein
BPHY_RS34640	-3,493	0,003926		Bphy_6973	porin
BPHY_RS34665	-1,994	0,004427		Bphy_6979	FAD-dependent oxidoreductase
BPHY_RS34725	1,470	5,38E-05		Bphy_6991	sensor histidine kinase
BPHY_RS34740	1,147	0,000793		Bphy_6994	LacI family transcriptional regulator
BPHY_RS34795	2,738	2,02E-08		Bphy_7005	AMP-binding protein
BPHY_RS34800	3,748	2,93E-37		Bphy_7006	AMP-binding protein
BPHY_RS34820	2,138	4,83E-06		Bphy_7011	ABC transporter permease
BPHY_RS34825	3,002	5,14E-16		Bphy_7012	universal stress protein
BPHY_RS34850	1,141	0,000973	tssL	Bphy_7017	type VI secretion system protein TssL, longform
BPHY_RS34860	1,342	6,25E-06		Bphy_7020	hypothetical protein
BPHY_RS34870	1,689	5,88E-09	tssI	Bphy_7022	type VI secretion system tip protein VgrG
BPHY_RS34875	1,951	3,36E-11	tssF	Bphy_7023	type VI secretion system baseplate subunit TssF
BPHY_RS34900	2,314	1,67E-15	acsA	Bphy_7028	acetate--CoA ligase
BPHY_RS34905	1,640	2,57E-05	pdhA	Bphy_7029	transferring) E1component subunit alpha
BPHY_RS34915	2,502	1,95E-12		Bphy_7031	2-oxo acid dehydrogenase subunit E2
BPHY_RS34920	4,640	4,66E-24		Bphy_7032	acyl carrier protein
BPHY_RS34925	6,474	1,06E-98		Bphy_7033	CBS domain-containing protein
BPHY_RS34930	4,619	1,91E-18		Bphy_7035	CBS domain-containing protein
BPHY_RS34935	1,588	0,007038		Bphy_7036	hypothetical protein

BPHY_RS34945	2,016	0,001759		Bphy_7038	erythromycin esterase family protein
BPHY_RS34950	2,719	1,87E-06		Bphy_7039	phosphoribosyltransferase
BPHY_RS34955	3,140	7,74E-05		Bphy_7040	dienelactone hydrolase family protein
BPHY_RS34960	3,609	1,92E-22		Bphy_7041	hypothetical protein
BPHY_RS34965	2,503	0,000348		Bphy_7042	ribose-phosphate diphosphokinase
BPHY_RS34990	1,753	0,000497		Bphy_7047	response regulator transcription factor
BPHY_RS35045	-2,614	0,00379		Bphy_7058	haloacid dehalogenase-like hydrolase
BPHY_RS35080	1,221	0,00234		Bphy_7067	phospholipase C
BPHY_RS35120	5,247	7,57E-39		Bphy_7075	hemerythrin domain-containing protein
BPHY_RS35150	1,388	0,004089	flhC	Bphy_7082	flagellar transcriptional regulator FlhC
BPHY_RS35155	2,470	1,34E-07	flhD	Bphy_7083	flagellar transcriptional regulator FlhD
BPHY_RS35180	1,429	0,000102		Bphy_7088	LysR family transcriptional regulator
BPHY_RS35220	1,045	0,008202		Bphy_7096	MOSC domain-containing protein
BPHY_RS35225	1,855	2,75E-05		Bphy_7097	nitronate monooxygenase
BPHY_RS35230	1,306	0,001227		Bphy_7098	creatininase family protein
BPHY_RS35250	-1,719	0,003592		Bphy_7102	hypothetical protein
BPHY_RS35270	1,487	0,000218	treF	Bphy_7106	alpha,alpha-trehalase TreF
BPHY_RS35290	-2,880	0,001101		Bphy_7110	bifunctional diguanylatecyclase/phosphodiesterase
BPHY_RS35370	-6,786	9,53E-05		Bphy_7129	hypothetical protein
BPHY_RS35420	-6,487	0,000456		Bphy_7138	phage terminase small subunit P27 family
BPHY_RS35440	-4,378	0,004121		Bphy_7143	S49 family peptidase
BPHY_RS35445	-2,946	0,001253		Bphy_7144	phage major capsid protein
BPHY_RS35465	-6,792	0,000264		Bphy_7148	HK97 gp10 family phage protein
BPHY_RS35490	-3,313	0,000365		Bphy_7153	phage tail length tape measure family protein
BPHY_RS35515	-1,774	0,00991		Bphy_7158	phage tail protein
BPHY_RS35570	1,236	0,00035		Bphy_7169	SDR family NAD(P)-dependent oxidoreductase
BPHY_RS35615	-2,769	0,000985		Bphy_7178	MFS transporter
BPHY_RS35780	-2,001	0,000278		Bphy_7212	efflux RND transporter permease subunit
BPHY_RS35840	-2,152	0,000951		Bphy_7224	enoyl-CoA hydratase/isomerase family protein
BPHY_RS35855	-8,083	1,69E-07		Bphy_7227	ABC transporter substrate-binding protein
BPHY_RS35875	-4,466	0,000278		Bphy_7231	cytochrome c
BPHY_RS35890	-3,455	0,001852		Bphy_7234	ABC transporter permease
BPHY_RS35900	-3,183	0,001013		Bphy_7236	ABC transporter substrate-binding protein
BPHY_RS35905	-4,830	0,004693		Bphy_7237	methyltransferase domain-containing protein
BPHY_RS35920	-6,010	0,002878		Bphy_7241	hypothetical protein
BPHY_RS35940	-2,211	0,007229		Bphy_7245	hypothetical protein
BPHY_RS35945	-2,265	0,006479		Bphy_7246	mandelate racemase
BPHY_RS35965	1,723	0,00013	hypD	Bphy_7252	hydrogenase formation protein HypD
BPHY_RS35975	1,735	6,41E-08	hypF	Bphy_7254	carbamoyltransferase HypF
BPHY_RS35980	2,884	1,21E-18	hypB	Bphy_7255	hydrogenase nickel incorporation protein HypB
BPHY_RS35985	1,872	0,000189		Bphy_7256	hydrogenase maturation nickel metallochaperoneHypA
BPHY_RS35990	1,326	0,00282		Bphy_7257	HupK protein
BPHY_RS35995	2,701	9,1E-09	hybE	Bphy_7258	[NiFe]-hydrogenase assembly chaperone HybE

BPHY_RS36000	4,132	7,96E-38		Bphy_7259	hydrogenase expression/formation protein
BPHY_RS36005	3,945	2,85E-21		Bphy_7260	hydrogenase
BPHY_RS36010	2,677	2,23E-05		Bphy_7261	HypC/HybG/HupF family hydrogenase formationchaperone
BPHY_RS36015	2,076	0,000306		Bphy_7262	HyaD/HybD family hydrogenase maturationendopeptidase
BPHY_RS36020	2,843	1,02E-11	cybH	Bphy_7263	Ni/Fe-hydrogenase, b-type cytochrome subunit
BPHY_RS36025	5,099	1,2E-57		Bphy_7264	nickel-dependent hydrogenase large subunit
BPHY_RS36030	6,102	1E-52		Bphy_7265	hydrogenase small subunit
BPHY_RS36040	4,856	9,31E-54	ectB	Bphy_7266	diaminobutyrate--2-oxoglutarate transaminase
BPHY_RS36045	5,895	1,43E-65		Bphy_7268	GNAT family N-acetyltransferase
BPHY_RS36055	6,332	1,2E-99		Bphy_7270	cysteine hydrolase
BPHY_RS36060	5,825	8,21E-65	hypE	Bphy_7271	hydrogenase expression/formation protein HypE
BPHY_RS36065	5,141	3,56E-61	hypF	Bphy_7272	carbamoyltransferase HypF
BPHY_RS36100	1,216	0,002024		Bphy_7278	zinc finger, SWIM-type
BPHY_RS36130	1,131	0,000931		Bphy_7287	Tn3 family transposase
BPHY_RS36230	1,919	5,65E-08		Bphy_7306	Tn3 family transposase
BPHY_RS36285	1,411	8,94E-05		Bphy_7318	FRG domain-containing protein
BPHY_RS36290	1,713	0,000923		Bphy_7319	AAA family ATPase
BPHY_RS36295	2,278	7,85E-08		Bphy_7320	S8 family peptidase
BPHY_RS36360	-2,367	0,000396		Bphy_7334	ShlB/FhaC/HecB family hemolysinsecretion/activation protein
BPHY_RS36430	1,387	0,000152		Bphy_7349	hypothetical protein
BPHY_RS36490	1,698	0,003482		Bphy_7363	2,4-dihydroxyhept-2-ene-1,7-dioic acid aldolase
BPHY_RS36525	2,233	0,005312			hypothetical protein
BPHY_RS36545	2,364	0,00396		Bphy_7376	hypothetical protein
BPHY_RS36560	3,461	1,62E-15		Bphy_7379	hypothetical protein
BPHY_RS36565	2,792	8,5E-07		Bphy_7380	DUF1488 domain-containing protein
BPHY_RS36615	1,583	7,12E-05		Bphy_7390	tyrosine-type recombinase/integrase
BPHY_RS36645	2,821	2,34E-17		Bphy_7397	alpha/beta fold hydrolase
BPHY_RS36650	2,833	7,64E-05		Bphy_7398	IS5 family transposase
BPHY_RS36655	4,007	5,28E-22	ltrA	Bphy_7399	group II intron reverse transcriptase/maturase
BPHY_RS36660	5,224	1,08E-39		Bphy_7401	transposase
BPHY_RS36670	5,461	1,67E-77		Bphy_7406	aldehyde dehydrogenase family protein
BPHY_RS36675	6,060	1,46E-63	treS	Bphy_7407	maltose alpha-D-glucosyltransferase
BPHY_RS36680	3,221	3,67E-06		Bphy_7408	ABC transporter ATP-binding protein
BPHY_RS36710	2,478	3,73E-05		Bphy_7415	hypothetical protein
BPHY_RS36750	1,343	0,000465		Bphy_7423	LacI family transcriptional regulator
BPHY_RS36765	-3,604	0,002975		Bphy_7426	ATP-binding protein
BPHY_RS36800	2,453	6,64E-10		Bphy_7433	IS110 family transposase
BPHY_RS36905	2,065	1,77E-06	glcD	Bphy_7454	glycolate oxidase subunit GlcD
BPHY_RS36915	2,920	7,04E-09	glcF	Bphy_7456	glycolate oxidase subunit GlcF
BPHY_RS36920	2,035	2,78E-10		Bphy_7458	site-specific integrase
BPHY_RS36925	2,571	1,78E-09		Bphy_7459	tyrosine-type recombinase/integrase
BPHY_RS36985	2,827	0,009325		Bphy_7474	MaoC family dehydratase
BPHY_RS36990	4,269	9,83E-28		Bphy_7478	GHMP kinase

BPHY_RS37000	2,632	1,54E-19		Bphy_7480	ISL3 family transposase
BPHY_RS37005	1,727	0,00055		Bphy_7481	GntR family transcriptional regulator
BPHY_RS37025	1,319	0,002307		Bphy_7485	DMT family transporter
BPHY_RS37030	3,688	2,38E-25		Bphy_7486	ISL3 family transposase
BPHY_RS37180	1,438	2,67E-07		Bphy_7516	hypothetical protein
BPHY_RS37205	2,392	1,02E-08		Bphy_7521	hypothetical protein
BPHY_RS37210	1,354	0,000316		Bphy_7522	type IV secretory system conjugative DNAtransfer family protein
BPHY_RS37240	2,315	7,2E-06	virB11	Bphy_7526	P-type DNA transfer ATPase VirB11
BPHY_RS37255	2,118	7,35E-07		Bphy_7529	TrbG/VirB9 family P-type conjugative transferprotein
BPHY_RS37260	1,968	4,31E-05		Bphy_7530	type IV secretion system protein
BPHY_RS37265	2,596	1,05E-07		Bphy_7531	type IV secretion system protein
BPHY_RS37270	3,097	1,27E-09		Bphy_7532	hypothetical protein
BPHY_RS37275	2,403	4,21E-08		Bphy_7533	haloacid dehalogenase
BPHY_RS37280	2,103	1,28E-08		Bphy_7534	VirB4 family type IV secretion system protein
BPHY_RS37285	1,977	0,003478		Bphy_7535	VirB3 family type IV secretion system protein
BPHY_RS37290	2,504	1,78E-07		Bphy_7536	hypothetical protein
BPHY_RS37305	1,100	0,006518		Bphy_7539	PAS domain S-box protein
BPHY_RS37365	1,521	0,000909		Bphy_7560	hypothetical protein
BPHY_RS37370	1,343	0,001767		Bphy_7561	hypothetical protein
BPHY_RS37380	1,463	0,003243		Bphy_7563	hypothetical protein
BPHY_RS37385	2,061	3,36E-07		Bphy_7564	relaxase/mobilization nuclease domain-containingprotein
BPHY_RS37405	2,130	2,43E-05		Bphy_7568	hypothetical protein
BPHY_RS37425	1,607	0,000585		Bphy_7572	winged helix-turn-helix transcriptionalregulator
BPHY_RS37445	1,314	0,001497		Bphy_7577	SOS response-associated peptidase familyprotein
BPHY_RS37485	-1,940	0,000584		Bphy_7585	sensor domain-containing diguanylate cyclase
BPHY_RS37525	1,422	0,000451		Bphy_7593	site-specific integrase
BPHY_RS37530	2,022	3,28E-06		Bphy_7594	tyrosine-type recombinase/integrase
BPHY_RS37565	-2,869	0,005678		Bphy_7601	tyrosine-type recombinase/integrase
BPHY_RS37595	1,578	0,000686		Bphy_7607	integrase
BPHY_RS37725	5,450	4,68E-59		Bphy_7633	D-alanine--D-alanine ligase
BPHY_RS37730	4,095	4,05E-29		Bphy_7634	multi anti extrusion protein MatE
BPHY_RS37735	3,178	2,47E-14		Bphy_7636	AMP-binding protein
BPHY_RS37740	3,651	1,24E-28		Bphy_7637	SDR family oxidoreductase
BPHY_RS37755	1,614	1,2E-06		Bphy_7641	hypothetical protein
BPHY_RS37840	-3,174	0,006525	istA	Bphy_7663	IS21 family transposase
BPHY_RS37920	2,876	1,48E-13		Bphy_7681	transposase
BPHY_RS37925	3,244	1,41E-08		Bphy_7682	hypothetical protein
BPHY_RS37950	1,610	0,002863		Bphy_7685	IS6 family transposase
BPHY_RS37965	1,810	0,000153		Bphy_7688	class I SAM-dependent methyltransferase
BPHY_RS37970	1,028	0,003876	queE	Bphy_7690	7-carboxy-7-deazaguanine synthase QueE
BPHY_RS38010	2,395	2,06E-12		Bphy_7703	amidohydrolase family protein
BPHY_RS38020	4,271	5,2E-23		Bphy_7706	radical SAM protein
BPHY_RS38025	5,537	1,63E-67		Bphy_7707	glycosyltransferase

BPHY_RS38030	2,215	3,93E-10		Bphy_7709	substrate-binding domain-containing protein
BPHY_RS38035	2,568	3,31E-05		Bphy_7710	hypothetical protein
BPHY_RS38040	3,243	2,12E-29		Bphy_7711	efflux RND transporter permease subunit
BPHY_RS38045	1,749	0,004572		Bphy_7712	efflux RND transporter periplasmic adaptorsubunit
BPHY_RS38050	2,062	8,43E-07		Bphy_7713	efflux transporter outer membrane subunit
BPHY_RS38120	1,216	0,000772		Bphy_7727	LysR family transcriptional regulator
BPHY_RS38125	1,561	0,000173	nifA	Bphy_7728	nif-specific transcriptional activator NifA
BPHY_RS38130	3,462	4,38E-26	nifE	Bphy_7729	nitrogenase iron-molybdenum cofactorbiosynthesis protein NifE
BPHY_RS38135	2,697	6,18E-18	nifN	Bphy_7730	nitrogenase iron-molybdenum cofactorbiosynthesis protein NifN
BPHY_RS38140	3,449	1,47E-23	nifX	Bphy_7731	nitrogen fixation protein NifX
BPHY_RS38145	4,152	6,58E-21		Bphy_7732	hypothetical protein
BPHY_RS38150	3,493	1,47E-15	fdxB	Bphy_7733	ferredoxin III, nif-specific
BPHY_RS38155	3,336	9,59E-21		Bphy_7734	nitrogen fixation protein NifQ
BPHY_RS38160	3,457	5,06E-20		Bphy_7735	hypothetical protein
BPHY_RS38165	4,521	6,57E-24		Bphy_7736	4Fe-4S dicluster domain-containing protein
BPHY_RS38170	4,270	1,75E-27		Bphy_7737	FAD-dependent monooxygenase
BPHY_RS38175	3,636	1,82E-25		Bphy_7738	electron transfer flavoprotein subunitalpha/FixB family protein
BPHY_RS38180	4,158	6,79E-29		Bphy_7739	electron transfer flavoprotein subunit beta/FixAfamily protein
BPHY_RS38185	4,362	1,31E-25	nifW	Bphy_7740	nitrogenase stabilizing/protective protein NifW
BPHY_RS38190	3,730	6,75E-36	nifV	Bphy_7741	homocitrate synthase
BPHY_RS38195	4,277	3,33E-28	nifB	Bphy_7742	nitrogenase cofactor biosynthesis protein NifB
BPHY_RS38220	4,106	9,55E-29	nifT	Bphy_7747	putative nitrogen fixation protein NifT
BPHY_RS38225	3,365	7,24E-14		Bphy_7748	hypothetical protein
BPHY_RS38230	3,151	1,59E-17		Bphy_7749	winged helix-turn-helix domain-containingprotein
BPHY_RS38235	2,244	3,73E-05			hypothetical protein
BPHY_RS38240	4,566	8,48E-27		Bphy_7750	zinc ribbon domain-containing protein
BPHY_RS38245	5,292	8,03E-31		Bphy_7751	ankyrin repeat domain-containing protein
BPHY_RS38250	6,251	2,26E-48		Bphy_7752	hypothetical protein
BPHY_RS38255	5,299	7,47E-23	nifH	Bphy_7753	nitrogenase iron protein
BPHY_RS38260	5,498	8,33E-49	nifD	Bphy_7754	nitrogenase molybdenum-iron protein alpha chain
BPHY_RS38265	5,509	1,16E-48	nifK	Bphy_7755	nitrogenase molybdenum-iron protein subunitbeta
BPHY_RS38275	1,887	1,06E-07		Bphy_7756	AAA family ATPase
BPHY_RS38310	4,718	1,76E-43		Bphy_7766	cytochrome P450
BPHY_RS38315	2,063	2,31E-07		Bphy_7767	propionyl-CoA carboxylase
BPHY_RS38320	1,527	8,76E-05		Bphy_7768	indoleacetamide hydrolase
BPHY_RS38325	1,906	8,26E-09		Bphy_7769	FAD-dependent oxidoreductase
BPHY_RS38340	2,019	1,41E-08		Bphy_7771	sterol desaturase family protein
BPHY_RS38345	2,166	3,14E-07		Bphy_7773	hypothetical protein
BPHY_RS38360	5,125	3E-16		Bphy_7776	nitrogen fixation protein NifZ
BPHY_RS38365	3,552	2,45E-07	nifT	Bphy_7777	putative nitrogen fixation protein NifT
BPHY_RS38370	2,583	8,35E-12		Bphy_7778	2-aminoethylphosphonate--pyruvate transaminase
BPHY_RS38375	2,569	1,49E-12		Bphy_7779	PLP-dependent transferase
BPHY_RS38380	2,424	5,86E-11		Bphy_7780	fatty acid desaturase family protein

BPHY_RS38385	2,150	3,24E-09		Bphy_7781	gamma-glutamyl-gamma-aminobutyrate hydrolasefamily protein
BPHY_RS38395	2,291	8,9E-10		Bphy_7783	homoserine O-succinyltransferase
BPHY_RS38400	2,012	6,25E-07		Bphy_7784	glutamine synthetase family protein
BPHY_RS38405	2,016	1,05E-07		Bphy_7786	IS5 family transposase
BPHY_RS38420	2,386	9,72E-11		Bphy_7789	GntR family transcriptional regulator
BPHY_RS38425	1,635	6,11E-05		Bphy_7790	class I SAM-dependent methyltransferase
BPHY_RS38430	1,314	0,001946		Bphy_7793	LysR family transcriptional regulator
BPHY_RS38460	1,029	0,0059		Bphy_7798	ABC transporter substrate-binding protein
BPHY_RS38505	6,927	1,08E-60		Bphy_7807	hypothetical protein
BPHY_RS38515	4,990	1,9E-26	nifH	Bphy_7808	nitrogenase iron protein
BPHY_RS38520	5,289	6,94E-12		Bphy_7809	nitrogenase molybdenum-iron protein alpha chain
BPHY_RS38525	4,410	1,69E-35		Bphy_7810	LysR family transcriptional regulator
BPHY_RS38530	1,240	0,000351		Bphy_7811	EamA family transporter
BPHY_RS38540	1,184	0,004188		Bphy_7813	chorismate mutase
BPHY_RS38545	3,010	1,52E-08		Bphy_7815	iron-sulfur cluster assembly accessory protein
BPHY_RS38565	3,542	3,33E-25		Bphy_7821	1-aminocyclopropane-1-carboxylate deaminase
BPHY_RS38605	4,509	3,63E-21		Bphy_1118	hypothetical protein
BPHY_RS38645	1,216	0,005467		Bphy_2335	GAF domain-containing protein
BPHY_RS38685	-5,766	0,004644		Bphy_3391	HK97 gp10 family phage protein
BPHY_RS38745	1,689	8,22E-06		Bphy_3636	immunity 71 family protein
BPHY_RS38750	1,374	0,001009		Bphy_3638	immunity 71 family protein
BPHY_RS38795	-4,147	0,000196		Bphy_4337	OmpA family protein
BPHY_RS38875	1,438	6,93E-05		Bphy_6116	TagK domain-containing protein
BPHY_RS38880	1,895	2,62E-07		Bphy_6125	peptidoglycan DD-metalloendopeptidase familyprotein
BPHY_RS38930	-2,463	0,001228		Bphy_6764	hypothetical protein
BPHY_RS38960	2,306	3,45E-08		Bphy_6907	FRG domain-containing protein
BPHY_RS38980	2,294	1,7E-10		Bphy_6938	S8 family serine peptidase
BPHY_RS38995	-4,588	0,001146		Bphy_7128	hypothetical protein
BPHY_RS39040	3,959	4,46E-30		Bphy_7479	pyridoxal-phosphate dependent enzyme
BPHY_RS39060	-8,254	1,75E-08			APC family permease
BPHY_RS39085	4,623	1,92E-08	cydX	Bphy_0297	cytochrome bd-I oxidase subunit CydX
BPHY_RS39130	-3,808	0,003811		Bphy_2925	aldehyde dehydrogenase family protein
BPHY_RS39140	-4,211	1,52E-08			DUF2817 domain-containing protein
BPHY_RS39150	2,861	0,000435		Bphy_4985	Rrf2 family transcriptional regulator
BPHY_RS39245	1,436	0,000937		Bphy_0643	hypothetical protein
BPHY_RS39255	-2,965	0,00838		Bphy_0681	Plug domain-containing protein
BPHY_RS39315	2,357	1,23E-06			DNA-binding protein
BPHY_RS39350	2,096	0,001279		Bphy_1842	DUF1328 domain-containing protein
BPHY_RS39425	-1,480	0,000566		Bphy_2566	DUF3025 domain-containing protein
BPHY_RS39440	-2,190	3,85E-05	yidD	Bphy_3099	membrane protein insertion efficiency factorYidD
BPHY_RS39650	-4,327	0,001853	tssF	Bphy_3877	type VI secretion system baseplate subunit TssF
BPHY_RS39680	2,937	0,000597		Bphy_4202	hypothetical protein
BPHY_RS40040	2,226	1,31E-06		Bphy_6056	BON domain-containing protein

BPHY_RS40095	1,485	0,000766	Bphy_6123	hypothetical protein
BPHY_RS40195	3,892	3,62E-10	Bphy_6253	hypothetical protein
BPHY_RS40470	1,746	1,92E-07	Bphy_6963	hypothetical protein
BPHY_RS40510	6,171	1,95E-50	Bphy_7034	hypothetical protein
BPHY_RS40530	5,318	3,54E-21	Bphy_7076	hypothetical protein
BPHY_RS40600	1,756	0,009125		pirin family protein
BPHY_RS40650	1,305	0,001728	Bphy_7308	suppressor of fused domain protein
BPHY_RS40820	3,835	4,15E-38		IS5 family transposase
BPHY_RS40825	2,600	1,64E-13	Bphy_7471	glutamine amidotransferase class-II
BPHY_RS40875	2,294	4,91E-06		hypothetical protein
BPHY_RS40945	1,465	0,002945	Bphy_7595	tyrosine-type recombinase/integrase
BPHY_RS40995	3,119	2,38E-13	Bphy_7638	transposase
BPHY_RS41080	4,702	1,34E-60	Bphy_7704	hemerythrin domain-containing protein
BPHY_RS41140	3,271	1,07E-08	Bphy_7806	4Fe-4S dicluster domain-containing protein
BPHY_RS41210	2,312	3,38E-06		hypothetical protein
BPHY_RS41285	2,657	0,004636	Bphy_5260	paraquat-inducible protein A
BPHY_RS41385	-1,684	0,003795	Bphy_3149	YadA-like family protein
BPHY_RS41485	1,433	0,003867		IS5 family transposase
BPHY_RS41510	-3,205	0,004456		IS5 family transposase
BPHY_RS41535	2,791	0,000487		IS3 family transposase
BPHY_RS41545	1,958	0,000114		FAD-dependent oxidoreductase
BPHY_RS41630	2,802	0,000815	Bphy_1096	hypothetical protein
BPHY_RS41655	2,479	4,98E-07		hypothetical protein
BPHY_RS41660	-6,004	0,003793		hypothetical protein
BPHY_RS41685	1,924	2,87E-07		hypothetical protein
BPHY_RS41700	-1,122	0,009104	Bphy_2137	hypothetical protein
BPHY_RS41725	3,809	1,99E-26		hypothetical protein
BPHY_RS41740	-1,378	0,005425		hypothetical protein
BPHY_RS41770	-6,343	0,005882		hypothetical protein
BPHY_RS41790	2,208	2,32E-08		hypothetical protein
BPHY_RS41830	2,695	4,33E-10		hypothetical protein
BPHY_RS41875	2,316	1,53E-06		hypothetical protein
BPHY_RS41885	-6,484	0,000408	Bphy_4787	hypothetical protein
BPHY_RS41930	-6,988	0,000846	Bphy_5032	BON domain-containing protein
BPHY_RS41950	2,061	0,003202		hypothetical protein
BPHY_RS42010	-6,069	0,004819		hypothetical protein
BPHY_RS42165	1,621	0,008396		hypothetical protein
BPHY_RS42255	5,468	7,11E-75		hypothetical protein
BPHY_RS42335	2,217	0,000103	Bphy_7477	transposase
BPHY_RS42340	-6,371	0,005327		DUF1016 family protein
BPHY_RS42385	3,044	1,86E-06		hypothetical protein
BPHY_RS42390	4,531	2,22E-14		nitrogen fixation protein NifZ
BPHY_RS42430	6,086	3,39E-28		ankyrin repeat domain-containing protein

BPHY_RS42485	2,038	0,001492	Bphy_1580	hypothetical protein
BPHY_RS42500	2,425	2,36E-06	Bphy_2073	hypothetical protein
BPHY_RS42610	1,764	0,000729	Bphy_4309	hypothetical protein
BPHY_RS42715	1,737	0,002132		hypothetical protein
BPHY_RS42775	-5,855	0,004981	Bphy_6761	hypothetical protein
BPHY_RS42810	3,773	2,92E-06		hypothetical protein
BPHY_RS42820	5,464	5,63E-22		aminotransferase class III-fold pyridoxalphosphate-dependent enzyme
BPHY_RS42910	3,972	1,28E-17	Bphy_3688	hypothetical protein
BPHY_RS42930	1,589	5,56E-06		hypothetical protein
BPHY_RS42965	2,826	1,68E-14	Bphy_7822	IS6 family transposase
BPHY_RS43010	3,303	2,64E-12		hypothetical protein

[1] Locus identifier and description are given according to the Burkholderia database

[2] Old locus tag is given according to the data in GenBank (GCA_000020045.1)
